# Targeted and Untargeted Approaches Unravel Novel Candidate Genes and Diagnostic SNPs for Quantitative Resistance of the Potato (*Solanum tuberosum* L.) to *Phytophthora infestans* Causing the Late Blight Disease

**DOI:** 10.1371/journal.pone.0156254

**Published:** 2016-06-09

**Authors:** Teresa Mosquera, Maria Fernanda Alvarez, José M. Jiménez-Gómez, Meki Shehabu Muktar, Maria João Paulo, Sebastian Steinemann, Jinquan Li, Astrid Draffehn, Andrea Hofmann, Jens Lübeck, Josef Strahwald, Eckhard Tacke, Hans-Reinhardt Hofferbert, Birgit Walkemeier, Christiane Gebhardt

**Affiliations:** 1 Department of Plant Breeding and Genetics, Max-Planck Institute for Plant Breeding Research, Cologne, Germany; 2 Faculty of Agricultural Sciences, Universidad Nacional de Colombia, Bogotá, Colombia; 3 Biometris, Wageningen University, Wageningen, The Netherlands; 4 Department of Genomics, Life & Brain Center, Institute of Human Genetics, University of Bonn, Bonn, Germany; 5 SaKa-Pflanzenzucht GmbH & Co. KG, 24340, Windeby, Germany; 6 Bioplant GmbH, Ebstorf, Germany; 7 Böhm-Nordkartoffel Agrarproduktion GbR, Ebstorf, Germany; 8 Institute Jean-Pierre Bourgin, INRA, AgroParis Tech, CNRS, Université Paris-Saclay, Versailles, France; Nanjing Forestry University, CHINA

## Abstract

The oomycete *Phytophthora infestans* causes late blight of potato, which can completely destroy the crop. Therefore, for the past 160 years, late blight has been the most important potato disease worldwide. The identification of cultivars with high and durable field resistance to *P*. *infestans* is an objective of most potato breeding programs. This type of resistance is polygenic and therefore quantitative. Its evaluation requires multi-year and location trials. Furthermore, quantitative resistance to late blight correlates with late plant maturity, a negative agricultural trait. Knowledge of the molecular genetic basis of quantitative resistance to late blight not compromised by late maturity is very limited. It is however essential for developing diagnostic DNA markers that facilitate the efficient combination of superior resistance alleles in improved cultivars. We used association genetics in a population of 184 tetraploid potato cultivars in order to identify single nucleotide polymorphisms (SNPs) that are associated with maturity corrected resistance (MCR) to late blight. The population was genotyped for almost 9000 SNPs from three different sources. The first source was candidate genes specifically selected for their function in the jasmonate pathway. The second source was novel candidate genes selected based on comparative transcript profiling (RNA-Seq) of groups of genotypes with contrasting levels of quantitative resistance to *P*. *infestans*. The third source was the first generation 8.3k SolCAP SNP genotyping array available in potato for genome wide association studies (GWAS). Twenty seven SNPs from all three sources showed robust association with MCR. Some of those were located in genes that are strong candidates for directly controlling quantitative resistance, based on functional annotation. Most important were: a lipoxygenase (jasmonate pathway), a 3-hydroxy-3-methylglutaryl coenzyme A reductase (mevalonate pathway), a P450 protein (terpene biosynthesis), a transcription factor and a homolog of a major gene for resistance to *P*. *infestans* from the wild potato species *Solanum venturii*. The candidate gene approach and GWAS complemented each other as they identified different genes. The results of this study provide new insight in the molecular genetic basis of quantitative resistance in potato and a toolbox of diagnostic SNP markers for breeding applications.

## Introduction

Since the potato famine in Ireland and central Europe in the middle of the 19^th^ century, the oomycete *Phytophthora infestans* causing this catastrophe remained the worldwide most important pathogen in potato cultivation [1]. Accordingly, the improvement of genetic resistance of the cultivated potato was and still is an important objective in most potato breeding programs during the last hundred years. The difficulty in achieving this objective in a sustainable way lies in the genetic flexibility of *P*. *infestans*, which so far defeated every single gene for resistance (*R* gene) that was introgressed in the cultivated potato from wild relatives [1]. Besides *R* gene mediated resistance there is quantitative or field resistance to *P*. *infestans* [2]. Quantitative resistance is polygenic and partial. These properties increase the number of mutations required in the pathogen to defeat host resistance and decrease the selection pressure on pathogen populations. Quantitative resistance is therefore considered more durable than *R* gene mediated resistance [3]. However, high quantitative resistance to *P*. *infestans* is correlated with late plant maturity, a negative trait at least in zones with a temperate climate [3–5]. Plant maturity is a complex character influenced by day length. It defines the duration of the plant’s annual life cycle from sprouting, shoot growth, tuber initiation and flowering to tuber maturation and senescence. The difficulty in developing improved cultivars with high levels of quantitative resistance lies in breaking the correlation between plant resistance and late maturity, and in the combination of a yet unknown number of resistance factors with other agronomic qualities such as high yield, nutritional qualities and culinary traits. Phenotypic selection of such cultivars requires multi-year and location trials and is hampered by the lack of knowledge of the genes that underlie quantitative resistance. Breeding for resistance could be greatly facilitated by diagnostic DNA markers. Diagnostic DNA markers are either directly derived from allelic variation in genes that control quantitative resistance or are in strong linkage disequilibrium (LD) with those genes. The identification and validation of diagnostic markers requires association analysis [6] in populations of elite cultivars.

Numerous linkage studies between DNA markers and resistance to *P*. *infestans* have been conducted in experimental, bi-parental and mostly diploid mapping populations, which identified at least twenty four quantitative resistance loci (QRL) on the twelve potato chromosomes [7]. Thanks to these genetic studies there is no shortage of DNA markers linked with QRL in specific genetic backgrounds. However, very few markers have been demonstrated by association analysis to be diagnostic in populations of tetraploid potato varieties and advanced breeding materials, which originate from multiple parents. DNA markers specific for the *R1* gene for resistance to *P*. *infestans* [8], which is linked with a major QRL on potato chromosome V [9, 10] were diagnostic for increased quantitative resistance to late blight in three different variety panels in different geographical regions. This association was not independent from the maturity phenotype though [11–13]. Recently, *StCDF1* which is physically closely linked with *R1*, was identified as the gene that controls day length dependent tuberization. *StCDF1* is therefore a strong candidate for underlying a major QTL for plant maturity on potato chromosome V [14–18]. Two further markers derived from unknown genes with structural similarity to *R* genes showed association with field resistance to late blight in a panel of Dutch varieties [19]. The genomic position and the relationship of these markers with plant maturity has not been reported. The diagnostic markers with the so far strongest effect on quantitative resistance to *P*. *infestans* that was not confounded by late plant maturity, were identified by association mapping in a population of 184 tetraploid breeding clones [5]. In particular, two single nucleotide polymorphisms (SNPs) in the coding sequence of allene oxide synthase 2 (*StAOS2*) on potato chromosome XI explained approximately one third of the genetic variance of maturity corrected resistance (MCR) to late blight. AOS is a key enzyme in the biosynthesis of jasmonates, phytohormones that play important roles as signaling molecules in the plant defense response against pests and pathogens [20, 21]. Functional analyses of natural *StAOS2* alleles suggested that the *StAOS2* locus is one of the genetic factors controlling quantitative resistance to *P*. *infestans* [22, 23]. A SNP in a second allene oxide synthase gene, *StAOS1* on chromosome IV, also showed association with MCR, although with much smaller effect than *StAOS2* [5]. Recently, five additional genes were identified which were associated with MCR. Based on their annotation they function in the biosynthesis of phytosterols, lipids and chlorophyll, modify the cell wall or are involved in the response to fungal elicitors [24]. In view of the number of QRL against *P*. *infestans* mapped in potato, additional loci must contribute to quantitative resistance.

The first diagnostic markers for quantitative resistance to *P*. *infestans* were identified via the candidate gene approach. The basis was the assumption that natural DNA variation in genes functional in pathogen recognition (e. g. *R* genes), signal transduction (e. g. metabolism of signaling molecules such as jasmonates, ethylene and salicylic acid) and defense responses (e. g. pathogenesis-related genes such as glucanases, chitinases, proteases and protease inhibitors) is responsible for the quantitative variation of resistance. The candidate gene approach targets specific genes or gene classes and is biased toward existing knowledge of gene function, which is mostly obtained from functional studies in model organisms. Untargeted approaches such as comparative transcript, protein and metabolite profiling of resistant versus susceptible plants have the potential to discover novel candidate genes and pathways that were previously not considered in the context of pathogen resistance [23–27]. The most unbiased and comprehensive approach to discover diagnostic DNA markers are genome wide association studies (GWAS), which are based on thousands of SNPs distributed over the whole genome [28]. GWAS in potato became possible with the advent of a first generation 8.3 k SNP genotyping chip [29–32].

Here we report the results of three approaches to identify novel functional candidate genes for controlling quantitative resistance to *P*. *infestans*, which is not compromised by late plant maturity: first, a candidate gene approach targeted specifically at the jasmonate pathway and second, comparative transcript profiling as an unbiased source of functional candidate genes. SNPs in genes identified by these two approaches were evaluated for association with resistance to *P*. *infestans*. The third approach was untargeted GWAS using the 8.3k SolCAP SNP genotyping array. All three approaches were complementary and identified SNPs associated with MCR. The associated SNPs were located in genes that might have a functional role in quantitative resistance to *P*. *infestans* and possibly other plant pathogens.

## Results

### Targeted approach: Candidate genes from the jasmonate pathway

**Fig 1:**
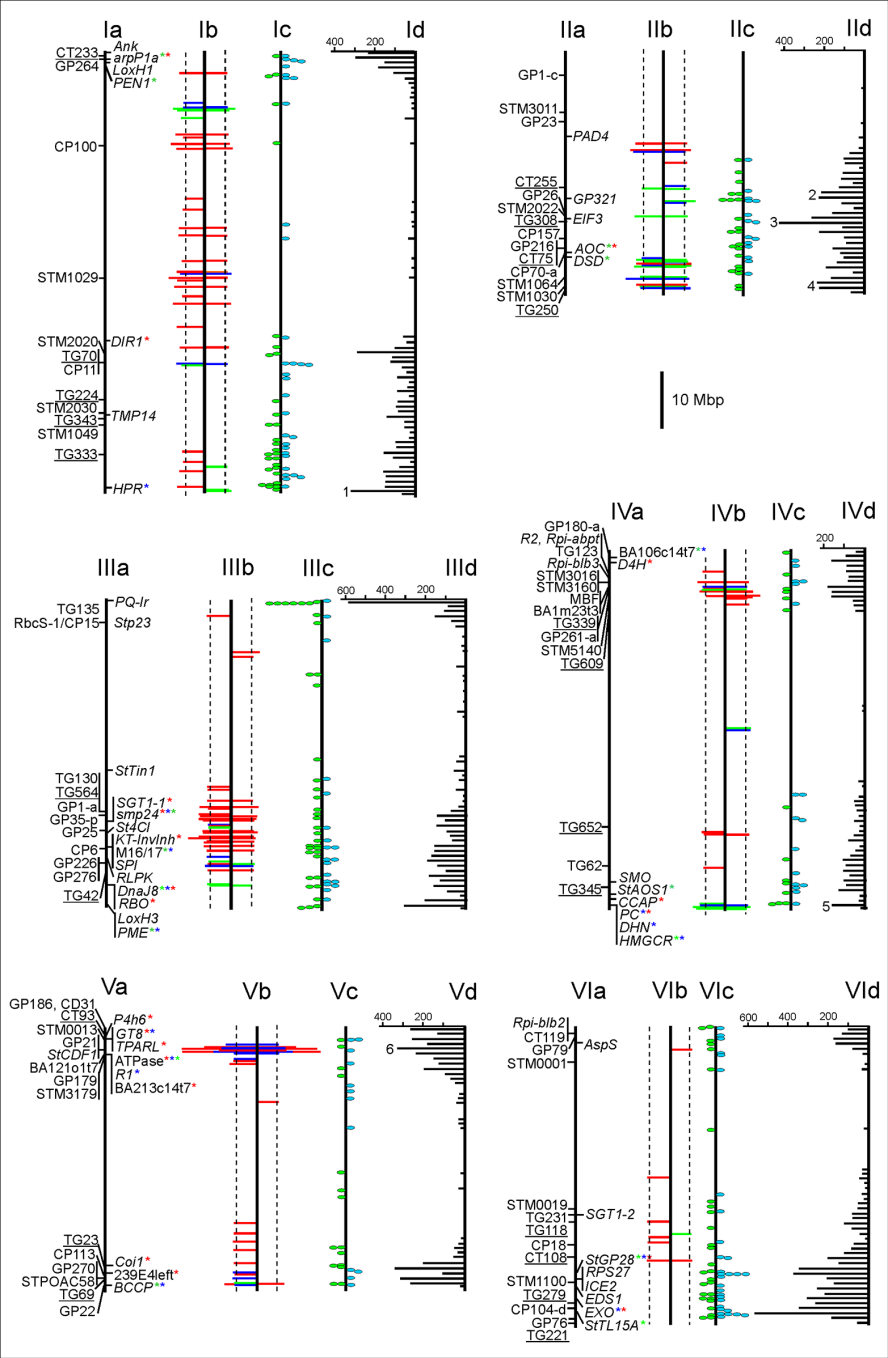
Physical maps of the potato chromosomes I to VI (pseudomolecules v4.03). Maps ‘a’ show to the left of the central bar (representing the chromosome) the positions of markers that were previously shown to be linked with *P*. *infestans* QRL in potato and the highly syntenic tomato (*Solanum lycopersicum*). Markers linked to *P*. *infestans* QRL in tomato are underlined (for physical positions and references see S8 File). The candidate loci tested for association with MCR, rAUDPC and PM in the PIN184 population previously [5, 11, 24, 34] and in this paper (S3 File) are shown to the right of maps ‘a’. Nine major genes for resistance to *P*. *infestans* (*R1*, *R2*, *R3*, *Rpi-blb1/RB*, *Rpi-blb2*, *Rpi-blb3*, *Rpi-vnt1*, *Rpi-abpt*, *Ph-3*) and the *StCDF1* locus controlling day length dependent tuberization are also included in maps ‘a’ (physical positions and references in S8 File). Loci harboring DNA variants associated with MCR, rAUDPC and/or PM are labelled with green, blue and red stars, respectively. Maps ‘b’ show the positions of the SolCAP SNPs that were associated with MCR (green bars), rAUDPC (blue bars) and PM (red bars) in the PIN184 population, on the left according to the association model without correction for population structure (model S) and on the right according to the association models correcting for population structure with different methods (K_1_, K_2_ and K_2_Q, see Materials and Methods). SolCAP SNPs associated with at least one trait with p < 10^−4^ were included. The length of the horizontal bars is proportional to the p-value. The dotted vertical lines indicate–Log10(P) = 4. Maps ‘c’ show on the right (blue dots) the positions of candidate genes that (i) harbor SNPs with different allele frequencies in the R8 and S8 genotype pools with high (R) and low (S) resistance to late blight (Materials and Methods), and (ii) had differential transcript levels between three genotype pools with different MCR levels in SuperSAGE analysis [23]. The green dots on the left of maps ‘c’ show the positions of candidate genes that (i) harbor SNPs with different allele frequencies in the R8 and S8 genotype pools, (ii) had differential transcript levels between the three genotype pools with different MCR levels in SuperSAGE, and (iii) were up or down regulated upon infection with *P*. *infestans* in SuperSAGE (for details see S7 File). Maps ‘d’ show the distribution of 42,688 SNPs with different allele frequencies in the R8 and S8 genotype pools (q < 0.01) on the potato pseudomolecules. The scale [number of SNPs per Mbp] is shown horizontally on top of each chromosome. SNP density peaks which overlapped with genomic segments harboring QTL for MCR or PM based on GWAS are indicated by numbers 1 to 8.

**Fig 2:**
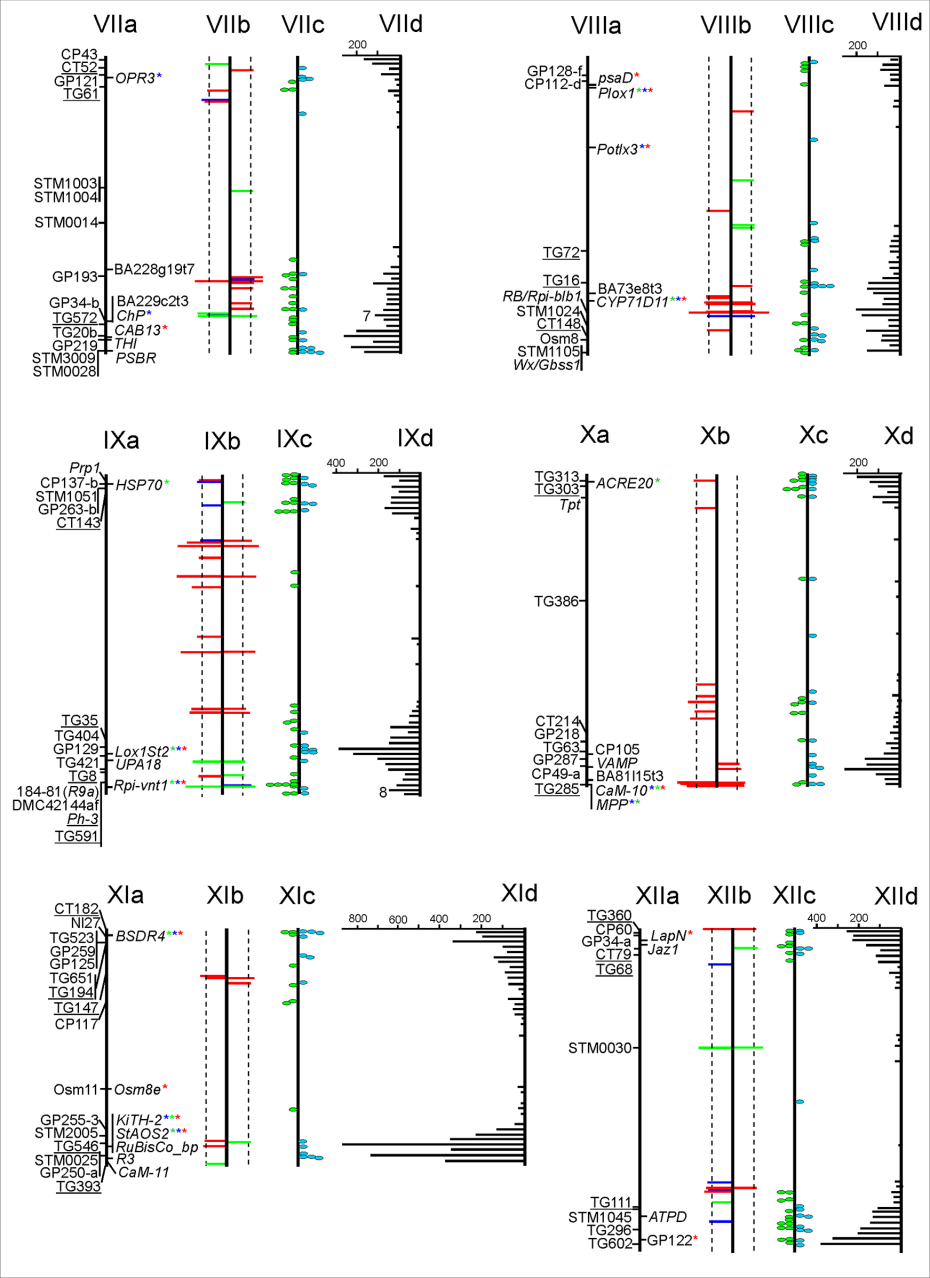
Physical maps of the potato chromosomes VII to XII (pseudomolecules v4.03). Legend as in Fig 1.

We have shown previously that SNPs in two genes encoding allene oxide synthase, a key enzyme in the biosynthesis of jasmonic acid and other oxylipins, were associated with MCR [5, 22]. To test whether other genes in the same pathway could also contribute to quantitative resistance, we selected for association analysis seven additional, cloned and characterized genes with functions in jasmonate biosynthesis: five lipoxygenases (*LOX*), allene oxide cyclase (*AOC)* and 12-oxophytodienoate reductase (*OPR*). Furthermore two genes involved in jasmonate signaling (*Coi1* and *Jaz1*) were selected according to the literature [20, 21] (Table 1). The genomic positions of these genes are included in Figs 1 and 2 (maps ‘a’). Locus identifier, physical position and annotation are provided in S3 File. Three of the nine genes were differentially expressed in a transcript profiling experiment performed previously by serial analysis of gene expression (SuperSAGE), either in response to infection with *P*. *infestans* (*Jaz1*) or between genotype pools with contrasting mean levels of MCR (*LoxH1*) or both (*Potlx3)* [23] (Table 1). The association panel consisted of 184 tetraploid potato cultivars (referred to as PIN184 population), for which the relative area under disease progress curve (rAUDPC), plant maturity (PM) and maturity corrected resistance (MCR) have been quantified based on replicated field trials [5]. Sequences of the corresponding potato genes were retrieved from GeneBank entries and the potato genome sequence [33]. The numerous sequences available for potato lipoxygenases were grouped according to sequence similarity and five representative *LOX* genes were selected. Amplicons comprising mainly exons of the nine genes were generated from the 184 individuals in the PIN184 population and sequenced (S1 File). One hundred and seventy one SNPs and two insertion/deletion polymorphisms (indels) were scored in approximately 2000 amplicon sequences (Table 1). A posteriori analysis showed that six of these SNPs (for SNP identification see S1 File) in four genes had different allele frequencies in the genotype pools R8 and S8 in RNA-Seq analysis (see below). The genotype pools R8 and S8 consisted of eight genotypes each that were selected for high (R) versus low (S) field resistance to late blight (see Materials and Methods). Association analysis using four statistical models (K_1_, K_2_, K_2_Q and S, see Materials and Methods) resulted in 15, 11 and 23 SNPs that were associated with MCR, rAUDPC and PM, respectively, with an error probability p < 0.01 in any of the association models (S4–S6 Files). Discounting redundant associations due to strong linkage disequilibrium (LD) between associated SNPs (*r*^*2*^ > 0.64) reduced the numbers to eleven (MCR), seven (rAUDPC) and sixteen (PM) marker-trait associations in seven, six and seven genes, respectively. Most consistent were the associations with late blight resistance (rAUDPC and/or MCR) in the loci *AOC*, *OPR3*, *Plox1* and *Lox1st2*, which were supported by three or four association models (Table 2). The most significant SNPs (p < 10^−4^) were Plox1_SNP8089 (in nearly complete LD with Plox1_SNP8344, S4 and S5 Files), which explained 15% to 16% of the phenotypic variation of MCR, and Lox1St2_SNP6571, which explained 5% to 6%. In both cases the minor frequency SNP alleles were associated with increased resistance. There was little overlap between marker associations with resistance and plant maturity. Consistent association with PM across three models was only observed for Coi1_SNP470 (Table 2, S6 File).

**Table 1:** Genes with functions in the jasmonate pathway that were tested for association with MCR, rAUDPC and PM.

Gene acronym	Chr.	Locus identifier PGSC0003	No. of SNPs/indels scored	Encoded protein	Locus selected in RNA-Seq ^e^	Locus selected in SuperSAGE	References
*LoxH1*	I	DMG400032155	18/1 (2) ^d^	13-lipoxygenase	Yes	Yes ^a^	[35]
*AOC*	II	DMG401012679	8 (0)	Allene oxide cyclase	No	No	[36]
*LoxH3*	III	DMG400022894	19 (2)	13-lipoxygenase	Yes	No	[35]
*Coi1*	V	Not annotated	36 (0)	Coronatine insensitive 1	-	-	[37]
*OPR3*	VII	DMG400030890	11 (1)	12-oxophytodienoate reductase 3	Yes	No	[38]
*Plox1*	VIII	DMG400020999	23 (0)	9-lipoxygenase	No	No	[39, 40]
*Potlx3*	VIII	DMG400010859	20 (0)	9-lipoxygenase	No	Yes ^a^^,^^b^	[40, 41]
*Lox1St2*	IX	DMG400031809	22 (1)	9-lipoxygenase	Yes	No	[40, 42]
*Jaz1*	XII	DMG400002930	14/1 (0)	Jasmonate-zim domain protein 1	Yes	Yes ^c^	[43]

^a^ Differential expression between quantitative resistant and susceptible genotype pools was found in SuperSAGE [23]

^b^ The transcript was up regulated upon infection with *P*. *infestans* in SuperSAGE [23]

^c^ The transcript was down regulated upon infection with *P*. *infestans* in SuperSAGE [23]

^d^ The number of SNPs scored that showed different allele frequencies in pools R8 and S8 in RNA-Seq analysis is shown in parenthesis (see S7 File for details).

^e^ Selection criterion: the locus contained SNPs with different allele frequencies (q < 0.01) in genotype pools R8 and S8.

**Table 2:** SNPs in candidate genes showing association with rAUDPC, MCR and/or PM in three or four association models with -Log10(P) > 2. Full data are available in S4–S6 Files.

Locus	Chr.	SNP ID	SNP alleles *phu/tbr*	Frequency (MFA) direction of effect ^c^	MCR—Log10(P) (R^2^) model ^d^	rAUDPC—Log10(P) (R^2^) model ^d^	PM—Log10(P) (R^2^) model ^d^
*arpP1a* ^a^	I	arpP1a_SNP791 ^b^	*A/C*	0.24 (C) ↑	2,57 (0.10) K_2_Q,K_2_,S	< 2.00	< 2.00
*arpP1a*	I	arpP1a_SNP781	*A/T*	0.35 (T) ↑	< 2.00	< 2.00	2.72 (0.11) K_2_,K_1_,S
*Pen1* ^a^	I	PEN1_SNP313	*T/A*	0.18 (A) **↓**	2.54 (0.10) K_2_Q,K_1_,K_2,_S	< 2.00	< 2.00
*AOC*	II	AOC_SNP152	*G/A*	0.06 (A) **↓**	< 2.00	2.18 (0.04) K_2_,K_2_Q,K_1_,S	< 2.00
*Smp24* ^a^	III	Smp24_SNP674 ^b^	*T/C*	0.30 (T) ↑	2.12 (0.07) K_2_,S	3.33 (0.10) K_2_,K_2_Q,S	3.69 (0.11) K_2_,S
*DnaJ8* ^a^	III	DnaJ8_SNP172 ^b^	*C/A*	0.20 (A) **↓**	< 2.00	2.92 (0.09) K_2_,K_2_Q,S	2.14 (0.06) K_2_
*DnaJ8*	III	DnaJ8_SNP277	*A/C*	0.13 (C) ↑	2.58 (0.09) K_2_Q,K_2_,S	< 2.00	< 2.00
*DH4* ^a^	IV	DH4_SNP5893 ^b^	*T/G*	0.06 (G) **↓**	< 2.00	< 2.00	3.06 (0.11) K_2_,K_1_,S
*DHN* ^a^	IV	DHN_SNP9817 ^b^	*A/T*	0.36 (T) **↓**	2.32 (0.09) K_2_,K_1_,S	< 2.00	< 2.00
*HMGCR*	IV	HMGCR_SNP455	*C/T*	0.38 (T) ↑	3.98 (0.13) K_2_Q,K_2_,K_1_,S	4.25 (0.14) K_2_Q,K_2_,K_1_,S	< 2.00
*HMGCR*	IV	HMGCR_SNP567	*C/G*	0.45 (G) **↓**	4.12 (0.14) K_2_,K_1_,K_2_Q,S	3.75 (0.13) K_2_,K_1_,K_2_Q,S	< 2.00
*HMGCR*	IV	HMGCR_SNP636 (solcap_c2_10566)	*C/T*	0.30 (T) **↓**	7.6 (0.19) K_1_,K_2_,K_2_Q,S	6.05 (0.15) K_1_,K_2_,K_2_Q,S	< 2.00
*GT8*	V	GT8_SNP266	*T/C*	0.44 (T) ↑	< 2.00	3.54 (0.15) K_2_,K_2_Q	4.70 (0.19) K_2_,K_2_Q,K_1_,S
*GT8*	V	GT8_SNP296 ^b^	*C/T*	0.05 (C) ↑	< 2.00	2.37 (0.07) K_1_,S	3.22 (0.12) K_2_,K_1_,K_2_Q,S
*ATPase*	V	ATPase_SNP7981 ^b^	*A/G*	0.37 (G) **↓**	< 2.00	3.24 (0.13) K_2_,K_2_Q,K_1_,S	8.20 (0.27) K_2_,K_2_Q,K_1_,S
*ATPase*	V	ATPase_SNP8102 ^b^	*C/T*	0.26 (T) **↓**	< 2.00	2.44 (0.11) K_2_,K_2_Q,K_1_,S	4.58 (0.18) K_2_,K_2_Q,K_1_,S
*ATPase*	V	ATPase_SNP8176 ^b^	*C/T*	0.23 (T) **↓**	< 2.00	2.68 (0.09) K_1_,K_2_,K_2_Q,S	4.13 (0.16) K_1_,K_2_,K_2_Q,S
*ATPase*	V	ATPase_SNP8218 ^b^	*C/T*	0.18 (T) **↓**	< 2.00	2.60 (0.11) K_2_,K_2_Q,S	5.60 (0.20) K_2_,K_2_Q,K_1_,S
*ATPase*	V	ATPase_SNP8491	*T/C*	0.11 (C) **↓**	2.47 (0.07) K_1_,K_2_Q,K_2_,S	4.64 (0.13) K_1_,K_2_Q,K_2_,S	3.41 (0.06) K_1_,K_2_Q,K_2_,S
*Coi1*	V	Coi1_SNP1690	*A/G*	0.10 (G) **↓**	< 2.00	2,29 (0.04) S	3.07 (0.09) K_2_,K_2_Q,S
*StGP28* ^a^	VI	StGP28_SNP957	*T/G*	0.37 (T) **↓**	3.24 (0.14) K_1_,K_2_Q,S	< 2.00	< 2.00
*StTL15A* ^a^	VI	StTL15A_SNP59972 ^b^	*T/G*	0.07 (T) **↓**	2.41 (0.06) K_2_,K_2_Q,S	< 2.00	< 2.00
*OPR3*	VII	OPR3_SNP713	*A/G*	0.24 (A) **↓**	< 2.00	3.38 (0.11) K_2_Q,K_2_,S	2.10 (0.06) K_2_Q
*CAB13* ^a^	VII	CAB13_SNP980	*C/A*	0.22 (A) ↑	< 2.00	< 2.00	2.40 (0.06) K_2_Q,K_2_,S
*psaD* ^a^	VIII	psaD_SNP2932	*G/A*	0.47 (A)	2.86 (0.06) S ↑ ^c^	< 2.00	3.40 (0.11) K_2_,K_1_,S ↓ ^c^
*Plox1*	VIII	Plox1_SNP8089	*A/C*	0.01 (C) **↓**	5.85 (15.3) K_2_Q,K_2_,K_1_,S	4.02 (11.2) K_2_Q,K_2_,K_1_,S	< 2.00
*CYP71D11* ^a^	VIII	CYP71D11_SNP346 ^b^	*C/T*	0.07 (T) **↓**	5.05 (0.14) K_2_,K_2_Q,K_1_,S	2.77 (0.08) K_2_,K_2_Q,K_1_,S	< 2.00
*CYP71D11*	VIII	CYP71D11_SNP706	*A/G*	0.05 (G) **↓**	4.56 (0.10) K_1_,K_2_Q,K_2_,S	3.47 (0.07) K_1_,K_2_Q,K_2_,S	< 2.00
*HSP70* ^a^	IX	HSP70_SNP8990	*C/T*	0.20 (T) **↓**	2.05 (0.08) K_2_Q,K_2_,K_1_,S	< 2.00	< 2.00
*Lox1St2*	IX	Lox1St2_SNP6571	*C/T*	0.12 (T) **↓**	3.37 (0.06) K_1_,K_2_Q,K_2_,S	4.22 (0.06) K_1_,K_2_Q,K_2_,S	< 2.00
*Lox1St2*	IX	Lox1St2_SNP6744	*A/T*	0.25 (T) ↑	2.89 (0.07) K_1_,K_2_,S	< 2.00	< 2.00
*Lox1St2*	IX	Lox1St2_SNP6762	*A/T*	0.08 (T)	2.28 (0.05) K_1_,K_2_,S ↑ ^c^	< 2.00	2.54 (0.05) S **↓** ^c^
*Rpi-vnt1*	IX	Rpi-vnt1_SNP440 (solcap_snp_c2_47952)	*T/C*	0.22 (C)	5.35 (0.16) K_2_,K_2_Q,K_1_,S **↓** ^c^	2.74 (0.09) K_2_,K_2_Q,K_1_ **↓** ^c^	2.10 (0.04) S ↑ ^c^
*Rpi-vnt1*	IX	Rpi-vnt1_SNP539	*A/G*	0.35 (G)	2.75 (0.05) K_1_,K_2_,K_2_Q,S **↓** ^c^	< 2.00	2.96 (0.10) K_2_,S ↑ ^c^
*Rpi-vnt1*	IX	Rpi-vnt1_SNP544	*C/T*	0.20 (T) **↓**	3.56 (0.09) K_2_Q,K_2_,S	3.00 (0.09) K_2_Q,K_2_,S	< 2.00
*CaM-10* ^a^	X	CaM-10_SNP7247	*G/T*	0.30 (G) **↓**	< 2.00	3.04 (0.10) K_2_,K_2_Q,S	5.21 (0.15) K_2_,S
*BSDR4*	XI	BSDR4_SNP339	*C/T*	0.36 (T) **↓**	2.74 (0.09) K_2_,K_2_Q,S	3.28 (0.11) K_2_,K_2_Q	6.10 (0.18) K_2_,K_1_,S
*BSDR4*	XI	BSDR4_SNP444	*A/T*	0.07 (T) **↓**	2.42 (0.08) K_2_,K_2_Q,S	2.67 (0.08) K_2_Q	< 2.00
*BSDR4*	XI	BSDR4_SNP469	*G/T*	0.22 (T) ↑	2.92 (0.07) K_2_Q,K_2_,S	2.15 (0.05) K_2_Q,K_2_,S	< 2.00
*BSDR4*	XI	BSDR4_SNP454 ^b^	*A/G*	0.35 (G) ↑	2.52 (0.09) K_2_,S	< 2.00	2.85 (0.09) K_2_,K_1_,S
*KiTH-2* ^a^	XI	KiTH-2_SNP3806	*T/A*	0.04 (A) **↓**	2.06 (0.08) K_2_,S	3.85 (0.13) K_2_Q,K_2_,S	2.79 (0.08) K_2_Q

^a^ Differential transcript levels were detected in SuperSAGE, see also Table 4.

^b^ The SNP showed differential allele frequency (q < 0.01) in RNA-Seq analysis

^c^ The arrows indicate the direction of effect of the minor frequency alleles (MFA): ↓ decreasing mean values for rAUDPC, MCR and PM, indicating greater resistance or later maturity; ↑ increasing mean values for rAUDPC, MCR and PM, indicating greater susceptibility or earlier maturity.

^d^ P and R^2^ values are shown for the first of the models listed.

### Untargeted approach: Novel candidate genes from comparative transcriptome sequencing (RNA-Seq)

**Fig 3:**
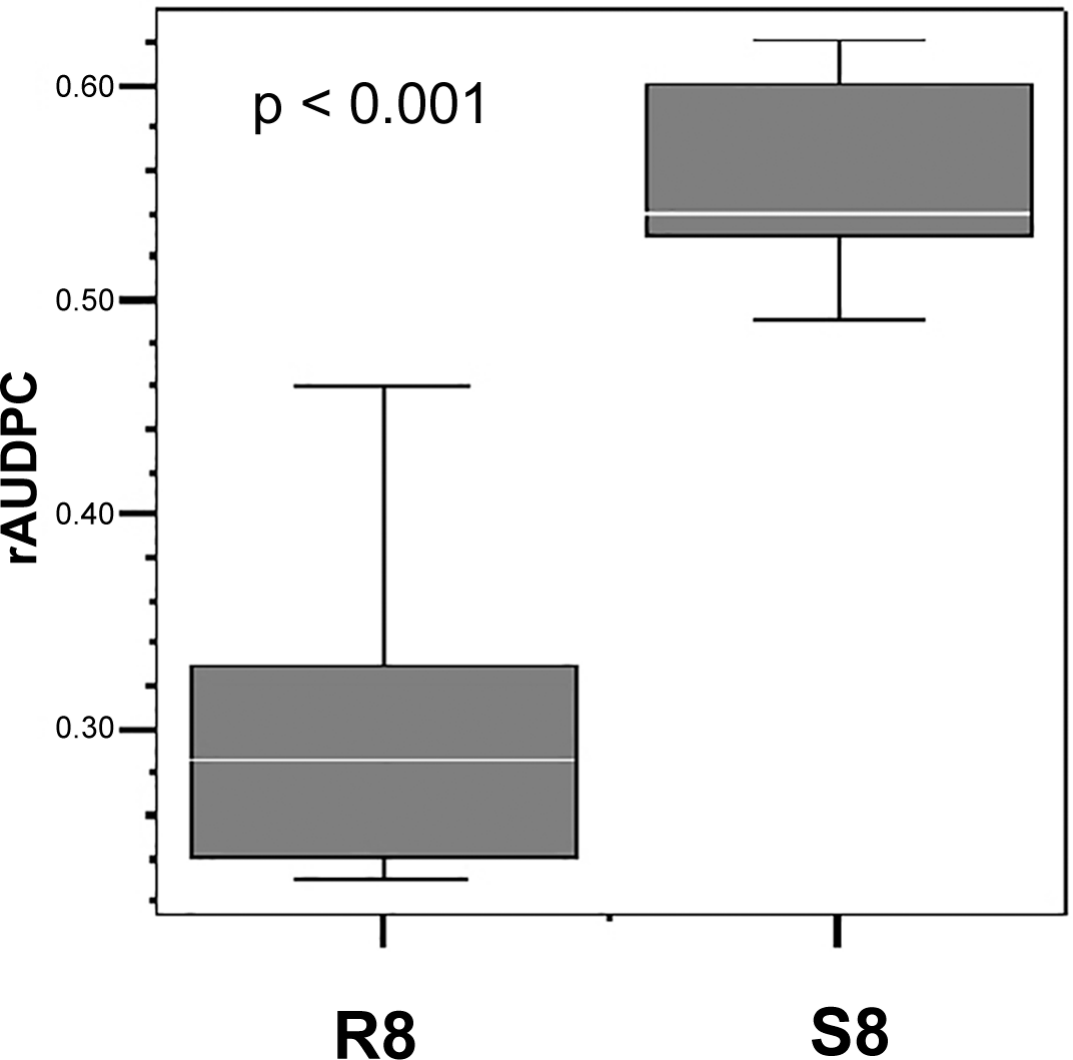
Boxplots of the rAUDPC values of eight quantitative resistant (R8) and susceptible (S8) tetraploid potato genotypes used to construct the samples for RNA-Seq analysis.

Four normalized cDNA libraries were constructed from the leaf RNA samples R8-T0, R8-T1T2, S8-T0 and S8-T1T2, and sequenced. Each sample was composed of eight tetraploid, heterozygous genotypes before (T0), one (T1) and two (T2) days after controlled infection with *P*. *infestans* (Materials and Methods). Sequencing yielded 69.8, 63.4, 60.6 and 55.6 million paired-end reads for sample R8-T0, R8-T1T2, S8-T0 and S8-T1T2, respectively. The raw sequences are available in GeneBank under the Bioproject number PRJNA294975 (accessions SAMN04044570, SAMN04044571, SAMN04044572, SAMN04044573). In order to maximize the chance to detect significantly different SNP allele frequencies between quantitative resistant (R8) and susceptible (S8) genotype pools (Fig 3) irrespective of transcriptional induction or repression by *P*. *infestans* infection, we combined the reads from samples R8-T0 (uninfected plants with lower mean rAUDPC) and R8-T1T2 (infected plants with lower mean rAUDPC), and from samples S8-T0 (uninfected plants with higher mean rAUDPC) and S8-T1T2 (infected plants with higher mean rAUDPC). Thus two data sets R8 and S8 were generated with 133 and 116 million paired-end reads, respectively, of which 48% and 43% mapped to the reference potato genome sequence DM [33, 44] under the chosen parameters (Materials and Methods).

**Fig 4:**
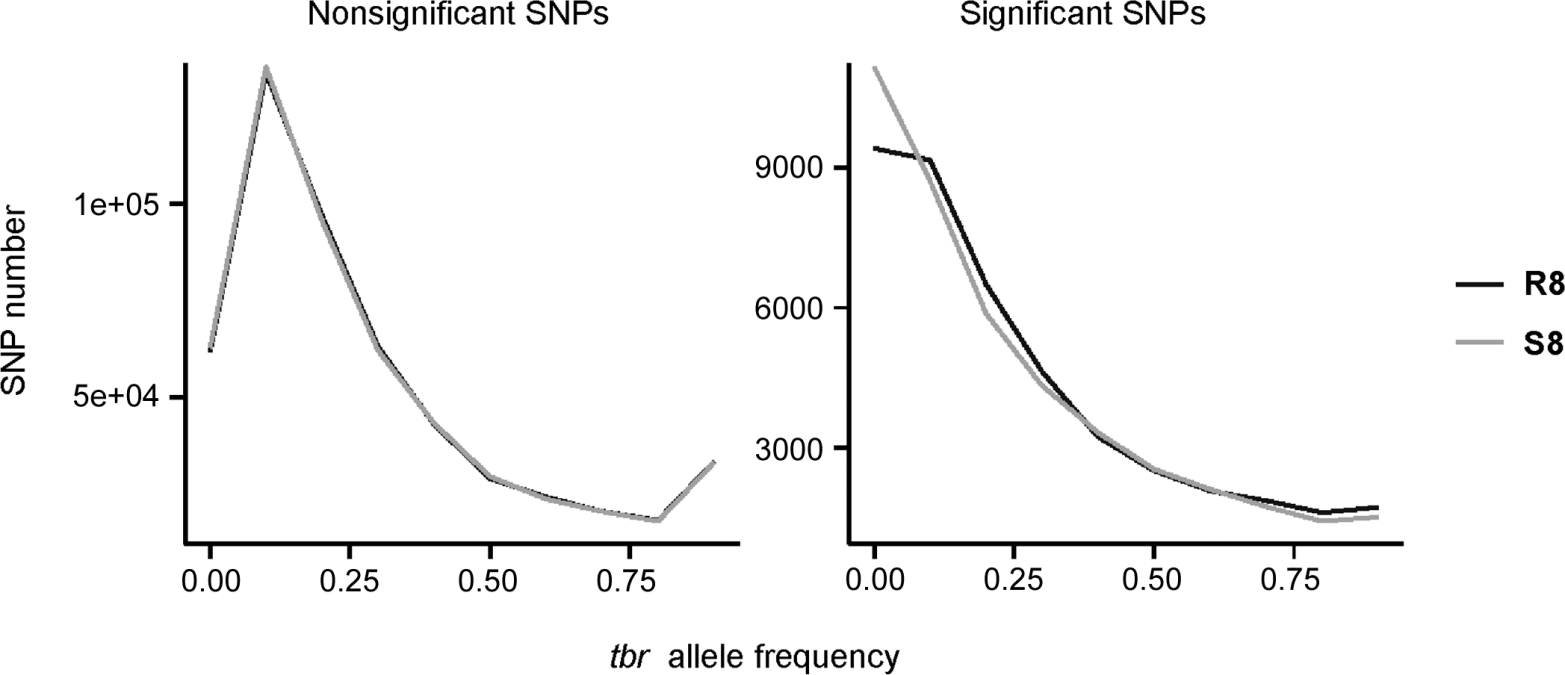
Frequency distribution of the *tbr* (*S*. *tuberosum*) SNP alleles with different allele frequencies (q < 0.01) in the R8 and S8 genotype pools (significant SNPs) and without significant differences (q > 0.01, non-significant SNPs).

The comparison of the SNP allele frequencies between R8 and S8 genotype pools resulted in 42 688 differential SNPs of total 566 805 SNPs (7.5%) when using q < 0.01 as cut-off value (S7 File). For simplicity we refer to the SNP allele of the reference *Solanum phureja* genome as the *phu* allele and to the alternative SNP allele as the *tbr* (*S*. *tuberosum*) allele, although the alternative allele could also originate from introgressions of *Solanum* species other than *S*. *tuberosum*. The majority of the *tbr* alleles of the significant SNPs were the minor frequency allele in both the R8 and S8 data sets, as shown by the frequency distributions skewed toward frequencies below 0.25. The frequency distribution of the non-significant *tbr* alleles was slightly different, with a maximum around 0.10 and a higher portion of alleles with frequencies above 0.8 (Fig 4).

The 42 688 differential SNPs were unequally distributed on the physical maps of the twelve potato chromosomes [44] (S13 File). The SNP density (number of SNPs per physical interval) increased toward the distal parts of the chromosome arms corresponding to gene rich euchromatic regions as compared to central, gene poor heterochromatic regions. On top of this general distribution distinct peaks were observed, most pronounced on chromosomes II, III, VI and XI (Figs 1 and 2, maps ‘d’). The SNPs StAOS2-SNP691 and StAOS2-SNP692 in the *StAOS2* coding sequence on chromosome XI, which showed the strongest association with MCR [5], have been used to assign plants to the R8 or S8 genotype pool (Materials and Methods). The genomic frequency of the *tbr* haplotype *StAOS2-G*_*691*_*G*_*692*_ in the R8 and S8 genotype pool was 0.12 and 0.78, respectively (Table 3). Differential allele frequencies for these two SNPs were therefore expected in the RNA-Seq analysis. Indeed, SNPs StAOS2-SNP691 and StAOS2-SNP692 were found as highly differential SNPs at position 41832747 and 41832748, respectively (S7 File), under the peak with the highest SNP density on the short arm of chromosome XI (873 differential SNPs in 55 genes between 41 and 42 Mbp, Fig 2, chromosome XId). The observed frequency of the *tbr* haplotype in the corresponding StAOS2 transcripts was 0.03 in genotype pool R8 and 0.81 in genotype pool S8 (S7 File). The second largest peaks (400–800 differential SNPs per Mbp) were on chromosomes II, III, VI and XI in the intervals 34–35 Mbp (58 genes), 0–1 Mbp (50 genes), 57–58 Mbp (66 genes) and 43–44 Mbp (49 genes), respectively (Figs 1 and 2, maps ‘d’). The peak on chromosome III could be largely attributed to a cluster of ten genes encoding chlorophyll a-b binding proteins (S7 File).

**Table 3:** *StAOS2* SNP genotypes and late blight resistance phenotypes of the potato plants used to compose the samples for RNA-Seq analysis.

Genotype	F1 family	*StAOS2 -SNP691*	*StAOS2 -SNP692*	rAUDPC 2009	rAUDPC 2010	Mean rAUDPC	Pool
BL114	1	*AAAA*	*CCCC*	0.22	0.28	0.25	R8
BL196	1	*AAAG*	*CCCG*	0.29	0.44	0.33	R8
BL201	1	*AAAG*	*CCCG*	0.28	0.36	0.32	R8
BL426	2	*AAAG*	*CCCG*	0.23	0.25	0.24	R8
BL499	2	*AAAG*	*CCCG*	0.22	0.27	0.24	R8
SL155	3	*AAAA*	*CCCC*	0.33	- ^a^	0.33	R8
SL312	4	*AAAA*	*CCCC*	0.32	0.14	0.23	R8
SL194	3	*AAAA*	*CCCC*	0.41	0.52	0.46	R8
	** **	* *	* *	μ = 0.29	μ = 0.32	μ = 0.31	
BL013	1	*GGGG*	*GGGG*	0.47	0.60	0.53	S8
BL024	1	*AGGG*	*CGGG*	0.48	0.58	0.53	S8
BL141	1	*AGGG*	*CGGG*	0.46	0.51	0.49	S8
BL238	1	*AGGG*	*CGGG*	0.52	0.59	0.55	S8
SL317	4	*AGGG*	*CGGG*	0.53	- ^a^	0.53	S8
SL316	4	*AGGG*	*CGGG*	0.57	0.66	0.61	S8
SL314	4	*AGGG*	*CGGG*	0.63	0.61	0.62	S8
SL433	4	*AGGG*	*CGGG*	0.55	0.64	0.59	S8
	** **			μ = 0.53	μ = 0.60	μ = 0.56	

^a^ Due to virus infection this clone had to be excluded from field evaluation in 2010

**Fig 5:**
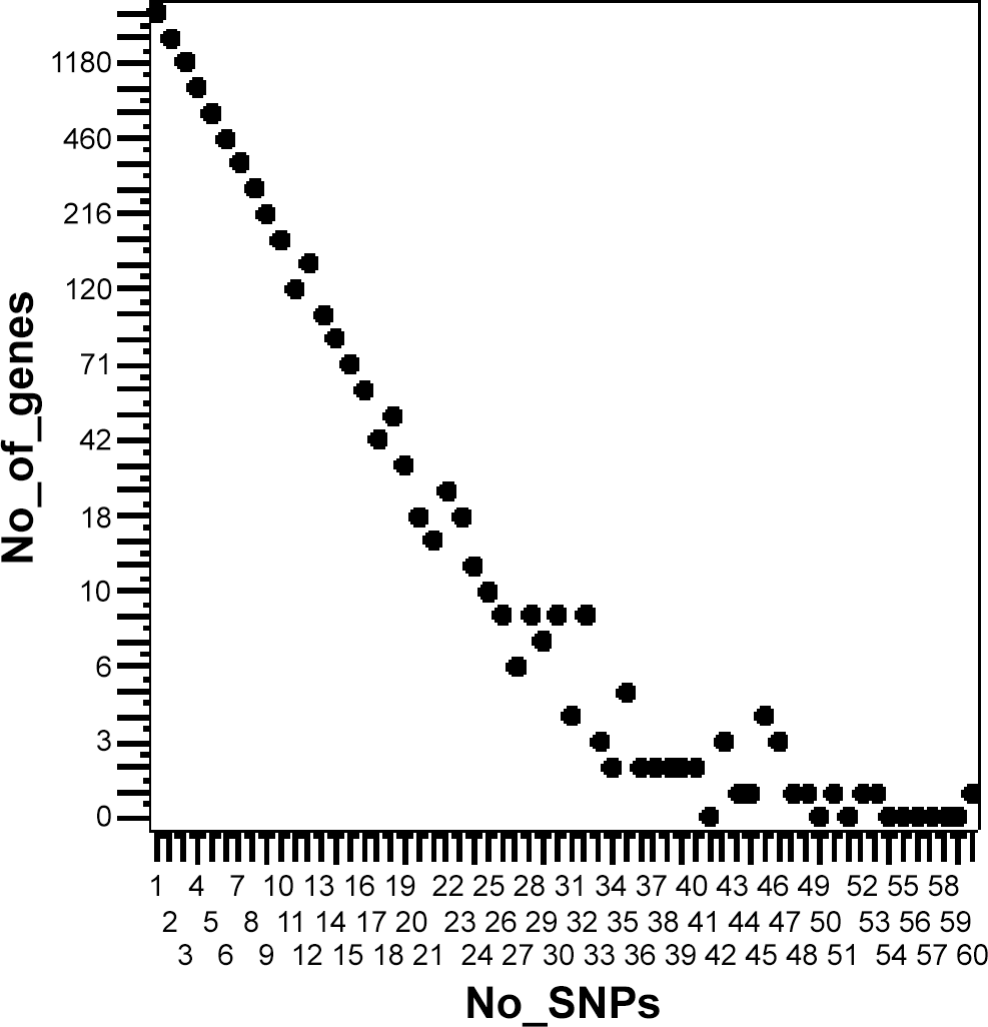
Number of genes containing from 1 to 60 SNPs with different allele frequency in the R8 and S8 genotype pools.

The differential SNPs were located in 9854 annotated genes, which represented approximately one quarter of all annotated potato genes [33]. The function of 18.3% of these genes is unknown. The number of differential SNPs per gene varied from 1 to 60. Forty nine percent (4829) of the genes contained only one or two differential SNPs (Fig 5). Twenty genes had 40 to 60 differential SNPs. Ten of these genes were annotated as chloroplastic proteins (six chlorophyll a-b binding proteins, RuBisCO large subunit-binding protein subunit alpha, pyruvate phosphate dikinase, 41 kD chloroplast nucleoid DNA binding protein, GcpE [45]). The remaining ten genes were annotated as a miraculin, an anthocyanidine rhamnosyl-transferase, a cytochrome P450 protein, a beta-galactosidase, a nucleoredoxin, myosin heavy chain, an ATP binding protein, a F-box and wd40 domain containing protein, a EF hand family protein and an unknown gene.

The large number of genes with differential SNPs, their genome wide distribution and the high SNP density particularly around the *StAOS2* locus suggested that factors other than contrasting levels of late blight resistance also contributed to the observed differential SNP allele frequencies, such as unbalanced genetic background between R8 and S8 genotype pools and linkage drag. We therefore used additional filtering criteria to select genes that might be genuinely involved in resistance to *P*. *infestans*. The 9854 genes detected in RNA-Seq analysis were compared via their locus identifier in the annotated potato genome (PGSC0003DMG40*******) with genes that were differentially expressed upon infection with *P*. *infestans* and/or in genotype pools with high and low levels of maturity corrected resistance (MCR) to late blight. These genes have been identified previously based on comparative transcript profiling by SuperSAGE. SuperSAGE was performed with 29 tetraploid genotypes that were combined in three genotype pools with contrasting levels of MCR [23]. The SuperSAGE experiment had six genotypes in common with the 16 genotypes used for the RNA-Seq analysis reported here. Controlled infection experiments with *P*. *infestans* and tissue sampling were performed under the same conditions in both types of transcriptome experiments. SuperSAGE had identified 2034 transcripts which were consistently up or down regulated in three genotype pools in response to infection with *P*. *infestans*. These transcripts matched to approximately 1830 potato loci, 1480 of which had a locus identifier. 1144 of the genes differentially expressed upon infection with *P*. *infestans* were identical with loci detected in RNA-Seq analysis (loci highlighted yellow in S7 File). This number was significantly higher than expected by chance (Fisher’s exact test for overrepresentation: p = 8.18^−20^). Furthermore, 806 transcripts derived from approximately 720 genes had shown differential transcript levels between the three genotype pools compared in the SuperSAGE experiment. Six hundred and thirty seven genes had a locus identifier, 513 of which showed SNPs with differential allele frequency in the RNA-Seq analysis (loci highlighted blue in S7 File) (Fisher’s exact test for overrepresentation: p = 9.79^−13^). Finally, 261 genes that had shown differential transcript levels in response to infection with *P*. *infestans* as well as between genotype pools in the SuperSAGE experiment, were also represented with differential SNPs in the RNA-Seq experiment (loci highlighted green in S7 File). The positions of the 261 ‘green’ and 252 ‘blue’ candidate genes on the physical map are shown in Figs 1 and 2 (maps ‘c’). The genomic distribution of these 513 candidate genes followed a similar general pattern as the differential SNPs, showing higher gene density in distal chromosomal regions. Particularly high densities of candidate genes were observed on the ‘South’ arms of chromosomes I, II, III, VI, VII, IX and XII, and on the ‘North’ arms of chromosomes III, IX, X and XII (Figs 1 and 2, maps ‘c’). In order to integrate the genomic distribution of candidate genes with the positions of genetically mapped quantitative resistance loci to *P*. *infestans*, we collected 149 DNA markers reported to be linked with *P*. *infestans* QRL in sixteen linkage mapping studies in potato [4, 9, 15, 16, 46–57] and two in the highly syntenic tomato [58, 59] (S8 File). These markers, as well as eight cloned potato *R* genes for resistance to *P*. *infestans* [8, 60–66], the recently cloned tomato late blight resistance gene *Ph-3* [67] and *StCDF1*, the gene proposed to underlie a major QTL for plant maturity [14] were anchored to the potato physical map (Figs 1 and 2, maps ‘a’). The distribution of QRL linked markers corresponded by and large to the distribution of candidate genes. Clustering of QRL linked markers within the distal 10–20 Mbp was observed on most chromosome arms.

Thirty five genes were selected for association analysis from the RNA-Seq experiment based on various criteria such as high number of differential SNPs per gene, very low q-values for differential SNP allele frequency, functional annotation and genomic position (overlap with QRL). Four additional genes (*PC*, *DHN*, *HMGCR* and *Rpi-vnt1*) were selected based on the results of GWAS (see below) (Table 4). Miraculin (PGSC0003DMG400010170), a member of a large family of protease inhibitors located in a prominent QRL region on the long arm of chromosome III (Fig 1), was one of the most outstanding candidate genes. It contained 47 SNPs with very low frequency of the *tbr* allele in the R8 genotype pool and a dramatic increase in the S8 pool (S7 File) plus differential transcript levels between genotype pools in SuperSAGE. Surprisingly, no DNA polymorphism was detected for this gene in the PIN184 population. For the remaining 38 genes we collected genotypic data of 483 SNPs and 11 indels by sequencing approximately 6500 amplicons in the PIN184 population (S1 File). One hundred and sixty seven of these SNPs (34%) showed different allele frequencies in the R8 and S8 genotype pools (Table 4). Association analysis using the models K_1_, K_2_, K_2_Q and S identified 62, 47 and 89 putative associations (p < 10^−2^) with MCR, rAUDPC and PM, respectively (S4–S6 Files). Discounting redundant associations of SNPs in strong LD with each other (*r*^*2*^ > 0.64) reduced the numbers to 49, 34 and 62 marker-trait associations at 21 (MCR), 18 (rAUDPC) and 26 (PM) candidate loci, respectively. Table 2 shows 34 SNP-trait associations at 19 loci that were supported by three or four association models. Thirteen of these (38%) were among the SNPs with different allele frequencies in R8 and S8 genotype pools in RNA-Seq analysis. The most robust associations with MCR (significant with all association models) were observed for ten SNPs at the loci *Pen1*, *HMGCR*, *ATPase*, *CYP71D11*, *HSP70* and *Rpi-vnt1*. Most significant (p < 10^−4^) were five SNPs at the loci *HMGCR*, *CYP71D11* and *Rpi-vnt1* on chromosome IV, VIII and IX, respectively (Table 2). The total variance of MCR explained by SNPs at these loci varied, depending on the association model and the SNP, between 10% and 21% (S4 File). SNPs at the loci *GT8* and *ATPase* on chromosome V showed the most robust and significant associations with plant maturity (Table 2, S6 File). One SNP in the *ATPase* gene, ATPase_SNP8491, was associated with MCR besides rAUDPC and PM (Table 2). The minor frequency allele of the associated SNPs was in 28 cases (82%) the *tuberosum* allele and in only six cases the *phureja* allele. In the majority of cases the minor frequency SNP allele was associated with increased resistance and/or later maturity (decreased mean values for rAUDPC, MCR and PM) (Table 2, S4–S6 Files). There were a few exceptions though, where the effects on resistance and maturity had the opposite direction. Most remarkable in this respect were the minor frequency SNP alleles *Rpi-vnt1_C*_*440*_ and *Rpi-vnt1_G*_*539*_, which were both associated with increased resistance and earlier plant maturity, although the effects on PM were small and only supported by one or two association models.

**Table 4:** Candidate genes with differential SNPs in RNA-Seq analysis that were selected for association analysis in the PIN184 population.

Gene ID	Chr.	Locus PGSC0003	No. of SNP/indelsscored	Annotation	Gene selected in SuperSAGE	Reference
*Ank*	I	DMG400019975	8 (4) ^a^	Ankyrin repeat-containing protein	No	[68]
*arpP1a*	I	DMG400032190	5 (3)	Acidic ribosomal protein P1a	Yes ^*b*^	[69]
*Pen1*	I	DMG400021331	23 (1)	Penetration1, syntaxin	Yes ^*d*^	[70]
*DIR1*	I	DMG400011323	11 (5)	Defective in induced resistance 1 protein	No	[71, 72]
*TMP14*	I	DMG400000204	4 (3)	Tylakoid membrane phosphoprotein 14 kDa	Yes ^*b*^^,^ ^*c*^	[73]
*EIF3*	II	DMG400029694	9 (8)	Eukaryotic translation initiation factor 3 subunit D-like	No	[74] http://www.uniprot.org/uniprot/O15371
*PQ-lr*	III	DMG400013431	10 (2)	PQ-loop repeat family protein; lysosomal cystine transporter family protein	No	[75]
*StTinI*	III	DMG400016749	16 (15)	TMV-induced protein I	Yes ^*d*^	[76]
*smp24*	III	DMG400019959	5 (2)	24 kDa seed maturation protein	Yes ^*d*^	[77]
*KT-InvInh*	III	DMG400010146	14/1 (1)	Kunitz-type tuber invertase inhibitor	No	[78, 79]
*SPI*	III	DMG400010170	0 (0)	Miraculin, serine protease inhibitor	Yes ^*b*^	[80, 81]
*DnaJ8*	III	DMG400014210	8/1 (2)	Heat shock protein binding protein 8	Yes ^*b*^	[82, 83]
*D4H*	IV	DMG400029517	14 (7)	Desacetoxyvindoline 4-hydroxylase	Yes ^*d*^	[84]
*PC*	IV	DMG400041620	20 (7)	Plastocyanin	Yes ^*b*^^,^ ^*c*^	[85]
*DHN*	IV	DMG400009968	11/2 (2)	25 kD dehydrin	Yes ^*b*^^,^ ^*c*^	[86]
*HMGCR*	IV	DMG400009924	10 (0)	3-hydroxy-3-methylglutaryl coenzyme A reductase	No	[87]
*GT8*	V	DMG400000827	14 (3)	Glycosyltransferase, CAZy family GT8	No	[88]
*TPARL*	V	DMG400000829	14 (5)	Transmembrane protein	No	[89] http://www.uniprot.org/uniprot/Q4V899
*ATPase*	V	DMG400031271	17/2 (15)	AAA-type ATPase	No	[90]
*StGP28*	VI	DMG402016495	15 (7)	Stem 28 kDa glycoprotein	Yes ^*b*^^,^ ^*c*^	[91]
*RPS27*	VI	DMG401028933	6 (4)	Ribosomal protein S27	Yes ^*b*^	[92]
*ICE2*	VI	DMG401028788	11 (4)	Inducer of CBF expression 2 protein (ICE), transcription factor	No	[93]
*EXO*	VI	DMG402005942	12 (3)	Endo-alpha-1,4-glucanase, beta-D-glucan exohydrolase	No	[94]
*StTL15A*	VI	DMG400034939	12 (8)	Thylakoid lumenal 15 kDa protein 1	Yes ^*b*^	[95]
*CAB13*	VII	DMG400019248	17 (7)	Chlorophyll a-b binding protein 13	Yes ^*b*^^,^ ^*c*^	[96]
*THI*	VII	DMG400019257	9 (3)	Chloroplast thiazole biosynthetic protein	Yes ^*b*^	[97]
*PSBR*	VII	DMG400022241	10 (0)	Photosystem II 10 kDa polypeptide	Yes ^*b*^	[98]
*psaD*	VIII	DMG400005805	18 (0)	Photosystem I reaction center subunit	Yes ^*b*^^,^ ^*c*^	http://www.uniprot.org/uniref/UniRef100_P49107
*CYP71D11*	VIII	DMG400020809	12 (4)	Cytochrome P450 71D11	Yes ^*d*^	[99]
*HSP70*	IX	DMG400008917	14 (2)	Heat shock protein 70kDa	Yes ^*b*^^,^^*d*^	[100]
*Rpi-vnt1*	IX	DMG400020587	16/1 (1)	Gene for resistance to *P*. *infestans* from *S*. *venturii*	No	[65]
*VAMP*	X	DMG400028151	13 (4)	Vesicle associated membrane protein SEC22	Yes ^*b*^	[101]
*CaM-10*	X	DMG400007205	9 (3)	Calmodulin	Yes ^*b*^	[102]
*BSDR4*	XI	DMG40003147	15 (4)	Bacterial spot disease resistance protein 4	No	[103]
*KiTH-2*	XI	DMG400008101	20/2 (0)	Kiwellin	Yes ^*d*^	[104]
*RuBisCo_bp*	XI	DMG400001148	20 (7)	RuBisCo large subunit-binding protein alpha subunit	Yes ^*c*^	[105]
*CaM-11*	XI	DMG400027384	12/1 (7)	Calmodulin	Yes ^*b*^	[102]
*LapN*	XII	DMG400007831	11/1 (1)	Leucine aminopeptidase N	No	[106]
*ATPD*	XII	DMG400016959	18 (13)	ATP synthase delta chain, chloroplastic	Yes ^*b*^	[107]

^a^ The number of SNPs with different allele frequencies in R8 and S8 genotype pools (RNA-Seq) is shown in parenthesis (details in S1 File).

^b^ Differential transcript levels between quantitative resistant and susceptible genotype pools in SuperSAGE [23].

^c^ The transcript was down regulated upon infection with *P*. *infestans* in SuperSAGE [23].

^d^ The transcript was up regulated upon infection with *P*. *infestans* in SuperSAGE [23].

### Untargeted approach: Genome wide association study (GWAS)

The PIN184 population was genotyped for 8303 SolCAP SNPs, which yielded 6286 polymorphic SNPs. LD and LD decay per chromosome were estimated using the physical map positions of 5600 SolCAP SNPs. The proportion of pair wise *r*^*2*^ values > 0.1 (loci in LD) and > 0.8 (loci in nearly complete LD) was 1.57 and 0.01 percent, respectively. The nonlinear regression curve in the plot of *r*^*2*^ values versus physical distance (not shown) reached the threshold of *r*^*2*^ = 0.1 between 270 and 280 base pairs, the same value as obtained previously with a small set of 36 potato varieties [32].

Association analysis using the four models K_1_, K_2_, K_2_Q and S (Materials and Methods) identified 281 SolCAP SNPs in 53, 53 and 172 loci (significant at p < 10^−4^) that were putatively associated with MCR, rAUDPC and PM, respectively (Table 5, S9–S11 Files). Twenty one of these loci were not annotated in the potato genome sequence. Annotations for most of those were retrieved from the orthologous loci in the highly syntenic tomato genome [108] (http://www.sgn.cornell.edu/). The majority of the annotated loci was represented in the RNA-Seq analysis by SNPs with different allele frequencies in R8 and S8 genotype pools. Some of these SNPs were even identical with associated SolCAP SNPs (Table 5, S9–S11 Files). Most conspicuous in this respect were three SNPs (solcap_snp_c1_11709, solcap_snp_c1_11710, solcap_snp_c2_39606) associated with MCR (S9 File), which are located in PGSC0003DMG400023590 encoding a 14-3-3 protein. Transcripts of this gene were upregulated upon infection with *P*. *infestans* [23]. Additional loci also showed differential expression in SuperSAGE (Table 5, S9–S11 Files).

**Table 5:** Number of SolCAP SNPs and corresponding loci showing putative associations with MCR, rAUDPC and PM based on different ranking criteria.

	MCR	rAUDPC	PM
No of SolCAP SNPs significant (p < 10^−4^) with 1 to 4 models	60	62	197
No of corresponding loci	53	53	172
No of loci with differential SNPs in RNA-Seq analysis	38	38	105
No of loci with differential expression in SuperSAGE	8	10	13
No of significant differential SolCAP SNPs in RNA-Seq analysis	7	9	20
No of SNPs significant (p < 10^−4^) with at least two association models	27	17	80
No of corresponding loci	24	16	67

Figs 1 and 2 (maps ‘b’) shows the positions of the SolCAP SNPs showing putative associations on the potato physical map (details in S9–S11 Files). As expected, the simple association model without correction for population substructure (model S) revealed the highest number of associations, particularly for PM (Figs 1 and 2, left side of maps ‘b’). Many of those were not detected with the models K_1_, K_2_ and K_2_Q, which corrected for substructure with different methods. The results of the K_1_, K_2_ and K_2_Q models (Figs 1 and 2, right side of maps ‘b’) differed with respect to p-values for the same SNP as well as number and identity of significant SNPs. The results of models K_2_ and S were similar, whereas models K_1_ and K_2_Q detected fewer associations, mostly with higher p values than models K_2_ and S. In many cases where different SNPs were significant with different association models, these SNPs mapped nevertheless to the same genomic segment (S9–S11 Files, Figs 1 and 2, maps ‘b’). Q-Q plots of the p-values obtained with models K_1_, K_2_, K_2_Q and S (S12 File) showed that K_1_ was the best fitting association model for all three traits, followed by the K_2_Q model for rAUDPC and PM. Q-Q plots for models K_2_ and S were similar for all traits.

One hundred and sixteen SolCAP SNPs in 98 loci were associated with MCR, rAUDPC and/or PM in at least two association models (S9–S12 Files). Five, five and seven SolCAP SNPs were associated with MCR, rAUDPC and PM, respectively, in three or four association models (Table 6). Taking into consideration the number of associated SNPs that mapped to a particular genomic segment, the significance and the robustness of the associations, we propose eleven genomic segments that harbor QRL for MCR based on GWAS (Table 7). Seven of those genomic segments overlapped with SNP density peaks from RNA-seq analysis (Table 7, Figs 1 and 2) The amount of variance explained by single SNPs in these regions varied between 8 and 18 percent (S9 File). The best candidate genes in these genomic segments were selected based on number and significance of the corresponding SolCAP SNPs, differential expression in SuperSAGE [23] and representation of the gene by differential SNPs in RNA-Seq analysis (Table 7). The two genes on chromosome I encoded an unknown gene (PGSC0003DMG400001190, 11.7 Mbp) and ‘Glycosyltransferase QUASIMODO1’ (PGSC0003DMG400014677, 88.3 Mbp). The four genes on chromosome II encoded ‘Cold regulated 314 thylakoid membrane 2’ (PGSC0003DMG400003083, 30.4 Mbp), ‘Transcription elongation factor 1 homolog’ (PGSC0003DMG400031773, 33.1 Mbp), ‘Xyloglucan endotransglucosylase-hydrolase XTH7’ (PGSC0003DMG400021398, 43.3 Mbp) and ‘Serine carboxypeptidase-like 25’ (PGSC0003DMG400001455, 45.6 Mbp). The candidate gene on chromosome III encoded ‘Hydroxycinnamoyl transferase’ (PGSC0003DMG400014152, 57.4 Mbp). The best candidate gene on chromosome IV was 3-hydroxy-3-methylglutaryl coenzyme A reductase *HMGCR* (PGSC0003DMG400009924, 71.95 Mbp). *HMGCR* and two additional candidate genes located at 71.25 Mbp (plastocyanin, *PC*) and 71.45 mbp (25 kDa protein dehydrin, *DHN*) were subsequently tested for association with MCR by amplicon sequencing (see above). The strongest association with MCR was observed for solcap_snp_c2_10566 in *HMGCR*, which was in nearly complete LD with solcap_snp_c1_3476 in the same gene. The latter SNP was represented with differential allele frequency in RNA-Seq (S9 File). Compared with the SNPs in *HMGCR*, the SNPs in the physical closely linked genes *PC* and *DHN* showed only minor associations with MCR (Table 2). The QRL on chromosome VII was detected with solcap_snp_c2_35100 by all four association models. This SNP is located in a C3HL domain class transcription factor (PGSC0003DMG400032829, 49.46 Mbp). The QRL on chromosome IX was identified by solcap_snp_c2_47952 in a homolog of *Rpi-vnt1*, a major gene for resistance to late blight from the wild species *S*. *venturii* [65]. The association with MCR was detected by all four models. Sequencing an amplicon including this SNP confirmed the association with MCR and detected additional associated SNPs (Table 2). Finally, the candidate gene on chromosome XII encoded a homolog of the polypyrimidine tract binding protein RBP50 that has been characterized in pumpkin [109] (PGSC0003DMG400018824, 22.90 Mbp).

**Table 6:** SolCAP SNPs associated with MCR, rAUDPC and/or PM in three or four association models (-Log10(P) > 4). Full data are available in S9–S11 Files.

Chr.	Position [Mbp]	SNP identifier	SNP alleles *phu/tbr*	Frequency (MFA) direction of effect ^d^	MCR—Lg10(P) (R^2^) model ^e^	rAUDPC—Log10(P) (R^2^), model ^e^	PM—Log10(P) (R^2^) model ^e^
I	45.8	solcap_snp_c2_40105 ^a^	*T/C*	0.20 (*C*) ↓	< 4.00	< 4.00	4.23 (0.11) K_1_, K_2,_ S
II	33.0	solcap_snp_c2_13051 ^a^	*A/C*	0.11 (*C*) ↓	4.79 (0.14) K_2,_ K_1_,S	< 4.00	< 4.00
II	45.6	solcap_snp_c1_4849 ^a^	*A/G*	0.16 (*G*) ↓	4.80 (0.10) K_1_,S	4.69 (0.15) K_2,_K_1_,S	< 4.00
III	48.0	solcap_snp_c2_53699 ^a^	*C/T*	0.34 (*T*) ↓	< 4.00	< 4.00	4.85 (0.19) K_1_,K_2_,S
IV	71.9	solcap_snp_c1_3476 ^a^^,^^b^	*T/C*	0.29 (*C*) ↓	5.12 (0.16) K_2_,K_1_,S	4.40 (0.09) S	< 4.00
V	3.7	solcap_snp_c2_11924 ^a^^,^^c^	*G/A*	0.17 (*A*) ↓	< 4.00	4.78 (0.14) K_2_,K_1_,S	< 4.00
V	4.0	solcap_snp_c2_11829 ^a^	*A/G*	0.20 (*A*) ↑	< 4.00	4.40 (0.09) S	8.22 (0.22) K_2_,K_2_Q,K_1_,S
V	4.6	solcap_snp_c2_22990 ^a^	*T/A*	0.17 (*T*) ↓	< 4.00	6.15 (0.15) K_2_,K_1_,K_2_Q,S	< 4.00
V	4.6	solcap_snp_c2_22989 ^a^	*C/T*	0.18 (*C*) ↑	< 4.00	4.74 (0.11) K_2_	11.87 (0.26) K_2_,K_1_,K_2_Q,S
V	5.0	solcap_snp_c2_50312	*A/G*	0.34 (*A*) ↑	< 4.00	4.20 (0.09) S	6.05 (0.17) K_2_,K_1_,S
V	5.0	solcap_snp_c2_50302 ^a^	*T/C*	0.19 (*T*) ↑	< 4.00	7.21 (0.18) K_2_,K_1_,K_2_Q,S	15.45 (0.34) K_2_,K_1_,K_2_Q,S
V	5.0	solcap_snp_c2_50298 ^a^	*T/A*	0.29 (*A*) ↓	< 4.00	7.00 (0.19) K_2_,K_1_,K_2_Q,S	4.35 (0.13) K_2_,S
VII	49.5	solcap_snp_c2_35100	*C/A*	0.08 (*A*) ↓	5.72 (0.13) K_2_Q,K_2_,K_1_,S	4.14 (0.08) S	< 4.00
IX	19.2	solcap_snp_c2_1918 ^a^	*T/A*	0.14 (*A*) ↓	< 4.00	< 4.00	8.08 (0.17) K_2_,K_1_,S
IX	59.6	solcap_snp_c2_47952 ^a^	*T/C*	0.18 (*C*) ↓	6.60 (0.18) K_1_,K_2_,K_2_Q,S	5.45 (0.10) K_1_	< 4.00
XII	22.9	solcap_snp_c1_3326 ^a^	*C/T*	0.06 (*T*) ↓	6.70 (0.14) K_2_,K_2_Q,S	< 4.00	< 4.00

^a^ The corresponding locus was present with differential SNPs in RNA-Seq analysis

^b^ The corresponding locus was down regulated upon infection with *P*. *infestans* in SuperSAGE [23]

^c^ The corresponding locus was up regulated upon infection with *P*. *infestans* in SuperSAGE [23]

^d^ The arrows indicate the direction of effect of the MFA: ↓ decreasing mean values for rAUDPC, MCR and PM, indicating greater resistance or later maturity; ↑ increasing mean values for rAUDPC, MCR and PM, indicating greater susceptibility or earlier maturity.

^e^ P and R^2^ values are shown for the first of the models listed.

**Table 7:** Genomic segments harboring QTL for MCR, rAUDPC and PM based on GWAS.

Genomic segment [Mbp]	Traits	Overlap with SNP density peak No. ^a^	Best candidate locus PGSC0003	Locus selected in RNA-Seq ^b^	Locus selected in SuperSAGE ^c^
Chr01: 11.5–12.5	MCR, rAUDPC	no	DMG400001190	yes	no
Chr01: 88.0–89.0	MCR	1	DMG400014677	yes	no
Chr02: 27.0–31.0	MCR, rAUDPC	2	DMG400003083	yes	yes ^d^
Chr02: 33.0–34.0	MCR	3	DMG400031773	yes	no
Chr02: 41.0–43.5	MCR, rAUDPC	no	DMG400021398	yes	yes ^d^
Chr02: 45.0–47.5	MCR, rAUDPC	4	DMG400001455	yes	no
Chr03: 56.5–57.5	MCR	no	DMG400014152	yes	no
Chr04: 71.0–72.5	MCR, rAUDPC	5	DMG400009924	yes	yes ^d^
Chr05: 3.0–5.5	rAUDPC	6	DMG400030565	yes	yes ^e^
	rAUDPC	6	DMG400018429	yes	no
	PM, rAUDPC	6	DMG400031262	yes	no
Chr07: 49.0–50.0	MCR	7	DMG400032829	no	no
Chr09: 59.0–60.0	MCR, AUDPC	8	DMG400020587	yes	no
Chr12: 22.0–23.0	MCR	no	DMG400018824	yes	no

^a^ The numbers identify the peaks in Fig 1, maps ‘d’

^b^ Selection criterion: the locus contained SNPs with different allele frequencies (q < 0.01) in genotype pools R8 and S8.

^c^ Selection criterion: the locus was consistently up or down regulated upon infection with *P*. *infestans* in three different genotype pools [23].

^d^ The transcript was down regulated upon infection with *P*. *infestans*.

^e^ The transcript was up regulated upon infection with *P*. *infestans***.**

Associations with rAUDPC and PM were detected mainly by twelve SolCAP SNPs in nine genes located between 3.0 and 5.5 Mbp on chromosome V (Fig 1, S10 and S11 Files). The best candidate genes for rAUDPC were ‘Fructose-bisphosphate aldolase’ (PGSC0003DMG400030565, 3.7 Mbp) and ‘Bacterial spot disease resistance protein 4’ (PGSC0003DMG400018429, 4.6 Mbp) (Table 7). The strongest association with both rAUDPC and PM was observed with solcap_snp_c2_50302 located in a methyltransferase gene (PGSC0003DMG400031262, 5.05 Mbp). This SNP explained, depending on the association model, up to 35 percent of the total variation of PM and up to 18 percent of the total variation of rAUDPC (Table 6). The methyltransferase gene and the *ATPase* candidate gene that was also strongly associated with PM and rAUDPC (Table 2), are separated by only 12 kbp. Both genes map approximately 510 kbp proximal to the *StCDF1* locus (at 4.54 Mbp). The gene pair annotated as ‘Bacterial spot disease resistance protein 4’ (PGSC0003DMG400018429 and PGSC0003DMG400018428, 4.6 Mbp) was physically most closely linked with *StCDF1* (distance 51 kbp). Solcap_snp_c2_22989 in PGSC0003DMG400018428 was associated with PM, whereas solcap_snp_c2_22990 in PGSC0003DMG400018429 was associated with rAUDPC (Table 6). Several additional genomic regions harbored QTL for PM with smaller effects and to less extent for rAUDPC (Table 6, Figs 1 and 2, maps ‘b’).

The highest density peaks of SNPs with differential allele frequency in RNA-Seq analysis on chromosomes III, VI and XI (Figs 1 and 2, maps ‘d’) did not overlap with the QTLfor late blight resistance identified by GWAS.

## Discussion

### Complementarity of approaches

The candidate gene approach for detecting marker-trait associations with quantitative resistance to late blight was first targeted at a specific pathway. After that, comparative transcript profiling was used for the unbiased discovery of novel candidate genes. A very small portion of those was subsequently tested for association with the traits MCR, rAUDPC and PM. The GWAS approach was untargeted and unbiased. All three approaches were complementary in that they identified different loci associated with maturity corrected resistance to late blight in the PIN184 population. However, none of the new SNP-MCR associations described here surpassed the effect of the two SNPs in the *StAOS2* gene, which were discovered previously with a candidate gene approach [5, 22, 110]. The QTL effect of *StAOS2* was neither detected by GWAS nor by twenty physically tightly linked SNPs in the *RuBisCo_bp* candidate gene from the RAD-Seq experiment, which is located right next to *StAOS2* within 14 kbp. This suggests that linkage disequilibrium in the PIN184 population did not extend much beyond the *StAOS2* locus, at least in this region of the potato genome. This is in line with the threshold of 270 to 280 base pairs for genome wide LD decay that was obtained by LD analysis of 5600 SolCAP SNPs in the PIN184 population, and provides further evidence that *StAOS2* is one of the genes that directly control MCR [22]. It further shows that the genome coverage of the 8.3 k SolCAP SNP array was insufficient for tagging all QTL for MCR in tetraploid potato. Nonetheless, GWAS based on this SNP array discovered several genes such as *HMGCR*, a C3HL domain class transcription factor and *Rpi-vnt1* that are considered as strong candidates for directly controlling MCR. These three genes were not detectable or not conspicuous in the RNA-Seq analysis.

### Assessment and ranking of marker-trait associations

Association analysis of 654 SNPs in 48 candidate genes and 6286 genome wide SolCAP SNPs was performed with four different statistical models. Models K_1_ and K_2_ corrected with different methods for kinship, K_2_Q corrected for kinship as well as population structure and the simple model S did not include any correction. As expected, model S resulted in the highest number of SNP-trait associations, many of which are likely false positives. However, several of those associations obtained with SolCAP SNPs were supported by different allele frequencies of the SNP in the R8 and S8 genotype pools (RNA-Seq analysis) or by differential expression of the corresponding gene in response to infection with *P*. *infestans* [23]. Association studies in the PIN184 and other, independent populations of tetraploid potato varieties and breeding clones have shown that there is no severe population structure in the central European germplasm pool of cultivated tetraploid potato [5, 32, 111–114], likely due to the breeding system. Potato breeding consists of intercrossing highly heterozygous, tetraploid parents and selecting superior genotypes in the segregating F1 generation, which are propagated vegetative. This system does not favor the formation of distinct heterotic groups as known in maize, for example. We therefore considered also the results of the simple model S when assessing the reliability of SNP-trait associations. Models K_1_ and K_2_ produced not the same results although the kinship matrices were calculated with the identical marker information. Based on Q-Q plots, model K_1_ provided the best fit of the four models. The results of model K_2_ were more similar to the simple model, indicating that the correction for population structure by model K_2_ was less stringent compared to model K_1_. The addition of population structure (K_2_Q model) improved the association model for the traits rAUDPC and PM but not for MCR. Despite the differences between the association models with respect to number and identity of associated SNPs and their p-values, these SNPs were frequently located in the same gene in the case of candidate genes, or in the case of GWAS, mapped to the same locus or the same physical region (Figs 1 and 2). As not all physically closely linked SNPs were in strong LD with each other and therefore redundant, it seems unlikely that multiple marker-trait associations tagging the same genomic region are false positives. SNP-trait associations were ranked, therefore, besides by error probability (p-value) according to the number of association models detecting the same SNP and the number of associated SNPs mapping to the same locus or genomic region. We considered as most reliable the SNP-trait associations detected by three or four models including model S (Tables 2 and 6) and the loci and physical regions that included several associated SNPs. Stand-alone SNP-trait associations were not considered reliable by whatever statistical model they were identified.

### Loci associated with quantitative resistance to *P*. *infestans* not compromised by late plant maturity

Association analysis of 171 SNPs in nine genes functional in the jasmonate pathway identified robust associations of SNPs with MCR and/or rAUDPC but not plant maturity, in the biosynthetic genes *Plox1*, *Lox1St2*, *AOC* and *OPR3* (Table 2, S4 and S5 Files). Most interesting for breeding applications are the rare haplotype *Plox1-C*_*8089*_*G*_*8344*_ (frequency 1.4%), which had the greatest positive effect on MCR and rAUDPC, and the minor frequency allele *Lox1St2_T*_*6571*_, which was also associated with increased resistance (Table 2). Both genes encode 9-lipoxygenases, which suggests that oxylipins other than jasmonic acid might have a role in MCR [21, 40]. The minor association with rAUDPC of a SNP in *OPR3* (OPR3_SNP713) is supported by an independent QTL linkage mapping experiment in diploid potato, where a restriction fragment length polymorphism in the *OPR3* coding sequence (not identical with OPR3_SNP713) was linked with a small effect QTL for late blight resistance [115].

Comparative transcript profiling of the genotype pools R8 and S8 having contrasting phenotypic means of resistance to *P*. *infestans* as well as contrasting genotypes at the *StAOS2* locus resulted in 42 688 differential SNPs in 9854 genes, corresponding to one quarter of all annotated potato genes [33]. Differential SNPs could theoretically arise only from different allele frequencies in pools R8 and S8, as the sequenced cDNA libraries were normalized. A portion of the differential SNPs likely resulted also from different expression levels due to incomplete transcript normalization. The sixteen F1 genotypes used for pool construction represented the genetic diversity of seven tetraploid, heterozygous parents, representing 28 genome haplotypes. This number was probably not sufficient for complete homogenization of the background genetic variation between the pools. Large haplotype blocks due to relatedness among the F1 genotypes causing linkage drag and the naturally very high DNA polymorphism in potato with one SNP every 20 to 30 base pairs [116] also generated random noise in the comparison between R8 and S8 pools. The assembly of the genotype pools was limited however by the fact that only 76 genotypes were available for selecting phenotypic and genotypic strongly contrasting genotypes. The large number of genes with differential SNPs made the rational selection of candidate genes for association analysis difficult. In retrospect, neither p-values nor number of differential SNPs per gene nor co-localization with genetically mapped QRL guarantied an efficient selection of our target genes, namely those which control quantitative resistance directly and would therefore show association of allelic DNA variation with the phenotypic variation of resistance to *P*. *infestans*. Despite the handicap associated with the genetic material used for RNA-Seq analysis, we identified by this approach genes such as *CYP71D11* that might be genuinely involved in quantitative resistance and provide novel diagnostic SNPs for breeding applications.

SNPs in twelve of 35 candidate genes selected based on RNA-Seq analysis showed robust associations with MCR and/or rAUDPC and none or only minor association with PM (Table 2, S4 and S5 Files). Except *ATPase* and *BSDR4*, these genes were differentially expressed either in response to infection with *P*. *infestans* (*PEN1*, *smp24*, *KiTH-2*, *HSP70*, *CYP71D11*) or between genotype pools with contrasting MCR levels (*arp1a*, *DnaJ8*, *StTL15A*, *Cam-10*) or both (*StGP28*) (Table 4) [23]. The functional annotation of *PEN1* and *BSDR4* links these genes directly with plant responses to pathogens triggered by *R* genes [70, 103]. The expression of *KiTH-2* (Kiwellin) was strongly up regulated upon infection with *P*. *infestans* and reached higher transcript levels in more resistant plants. *KiTH-2* is one of six clustered Kiwellin genes located 511 kbp proximal to the *StAOS2* locus on chromosome XI. LD was not observed between the haplotypes *KiTH-2-T*_*3689*_*A*_*3806*_*G*_*3987*_ and *StAOS2-A*_*691*_*C*_*692*_ both associated with greater resistance, which indicates that the effects on resistance observed at the two loci were independent. The role of *KiTH-2* in pathogen resistance is unknown. *DnaJ8*, *HSP70* and *smp24* have putative functions in general stress responses [77, 82, 100]. The closest Arabidopsis homolog of *DnaJ8* is chloroplastic *AT1G80920*, which plays a role in stabilizing photosynthetic complexes and in oxidative stress responses [83]. *StTL15A* encodes a 15kD protein of unknown function in the thylakoid lumen [95]. Two SNPs in the same gene, but different from StTL15A_SNP59972 identified in this study, were associated with resistance to *P*. *infestans* in a panel of diploid *S*. *phureja* clones evaluated in Colombia (Álvarez et al. submitted). Cam-10 (calmodulin) is a component of the Calcium signaling pathway, which plays important roles in plant development and pathogen interactions [102, 117]. *Arp1a* encodes one of the numerous ribosomal genes that were differentially expressed in plants with contrasting MCR levels [23]. The annotation of *StGP28*, *ATPase* and *CYP71D11* is highly unspecific and their possible role in host-pathogen interactions is unknown [90, 91, 118]. In the case of *CYP71D11* however, which encodes a cytochrome P450-dependent monooxygenase (CYP) on chromosome VIII, the genomic context suggests a function of this particular gene in terpene biosynthesis. The locus PGSC0003DMG400001948, which maps 36 kbp distal to *CYP71D11*, is annotated as 8-hydroxy-copalyl diphosphate synthase [119] and is a member of the terpenoid synthase (TS) superfamily. This gene was also represented with a single differential SNP in the RNA-Seq results (S7 File). TS’s and CYP’s are both encoded by multi gene families. They are key enzymes in terpene biosynthesis and determine the tremendous diversity of this largest class of plant secondary metabolites [99]. Both genes were up regulated upon infection with *P*. *infestans* [23]. Recently it was demonstrated that distinct pairs of TS and CYP genes are found together in several plant species within 50 kbp genomic sequence far more commonly than expected by chance, thus forming functional clusters. *CYP71D11* and copalyl diphosphate synthase were one of seven such CYP/TS gene pairs that were discovered in the potato genome [99]. Fourty eight SNPs with differential allele frequency were found in *CYP71D11*, which placed it among the top twenty genes with 40 to 60 differential SNPs per gene that were identified by RNA-Seq analysis. Three SNPs in strong LD with each other, one of those differential in RNA-Seq analysis, showed the most significant and robust associations with rAUDPC and MCR from all 35 genes tested. The SNPs explained between 6 and 21 percent of the phenotypic variation, depending on SNP and association model. The low frequency haplotype *CYP71D11-T*_*346*_*G*_*505*_*C*_*548*_ was associated with greater resistance. Allelic variation in this CYP and the neighboring TS gene could affect the quantity of specific secondary metabolites that inhibit *P*. *infestans* growth [120].

GWAS revealed 32 loci putatively associated with MCR and/or rAUDPC but not plant maturity, which were supported by at least two association models (S9 and S10 Files). The majority of these loci (72%) were supported in the RNA-Seq analysis by SNPs with different allele frequencies in genotype pools R8 and S8, which were genetically different from the PIN184 population. The genotype pools R8 and S8 represented the genetic diversity of 28 parental genome haplotypes, whereas the diversity of the PIN184 population representing 736 genome haplotypes (4 x 184) was much larger. SNPs in eight loci were identified by three to four association models (Table 6). In all cases the minor frequency allele was associated with greater resistance. Increasing the frequency of these alleles in breeding populations should improve the average resistance level. The putative function of these eight genes is discussed below.

The first locus PGSC0003DMG400031773 (solcap_snp_c2_13051) encodes a homolog of transcription elongation factor 1 (*TEF1*). Four SNPs in this gene were differential in the RNA-Seq analysis. TEF’s control the transcript elongation of subsets of genes in the chromatin context thus contributing to the control of gene expression [121]. The second locus PGSC0003DMG400001455 (solcap_snp_c1_4849) is annotated as ‘Serine carboxypeptidase-like 25’, a class of proteins with unclear biochemical function, not necessarily proteolysis [122]. Four SNPs, one identical with solcap_snp_c1_4849, showed different allele frequencies in R8 and S8 genotype pools. Both genes are located on the long arm of chromosome II, where QTL for late blight resistance have been repeatedly mapped in various genetic backgrounds (Fig 1, maps ‘a’).

PGSC0003DMG400009924 on chromosome IV encodes a 3-hydroxy-3-methylglutaryl coenzyme A reductase (HMGCR), the expression of which was down regulated after infection with *P*. *infestans* compared with uninfected plants [23]. One of two differential SNPs in the RNA-Seq analysis was identical with solcap_snp_c2_10566. The association of this SNP with MCR was confirmed by amplicon sequencing, which identified additional associated SNPs. The physically closely linked genes *PC* and *DHN* (within 100 kbp from *HMGCR*) were strong candidates based on differential expression in SuperSAGE as well as different SNP allele frequencies in R8 and S8 genotype pools (S7 File). Both genes showed nonetheless only minor associations compared with *HMGCR*. HMGCR is a key enzyme in the mevalonate pathway, which provides precursors for isoprenoid or triterpene biosynthesis. It might be part of the same metabolic network as *CYP71D11* for the synthesis of antifungal secondary metabolites. This particular gene is homologous but not identical with three HMGCR cDNA clones *hmg1*, *hmg2* and *hmg3* that have been isolated and characterized from potato tubers [123, 124]. The *HMGCR* on chromosome IV is therefore a novel, uncharacterized member of the potato *HMGCR* gene family. The expression of tuber *hmg*’s was differentially regulated by methyl jasmonate, the fungal elicitor arachidonic acid, by wounding and infection with *P*. *infestans* [123, 124], thus providing a link to the jasmonate signaling pathway.

The fourth locus PGSC0003DMG400030565 (solcap_snp_c2_11924) on chromosome V encodes one of seven fructose-bisphosphate aldolase (*FBA*) genes, functional in the primary metabolic pathways of glycolysis, gluconeogenesis and Calvin cycle. Arabidopsis FBA’s showed differential expression patterns in response to abiotic stress [125]. This particular *FBA* gene was represented with eleven differential SNPs in the RNA-Seq result. Its transcript level increased upon infection with *P*. *infestans* in SuperSAGE. The fifth locus PGSC0003DMG400018429 (solcap_snp_c2_22990) is one of two genes duplicated within 30 kbp on chromosome V. The loci PGSC0003DMG400018429 (*StBs4-1*) and PGSC0003DMG400018428 (*StBs4-2*) encode potato homologs of the tomato ‘bacterial spot disease resistance protein 4’ (*Bs4*) that is located in the syntenic genome segment on tomato chromosome 5 [103, 126]. Both loci were represented with differential SNPs in the RNA-Seq results, *StBs4-1* with 23 and *StBs4-2* with 16 SNPs (S7 File). Interestingly, solcap_snp_c2_22990 in *StBs4-1* was associated with rAUDPC, whereas solcap_snp_c2_22989 in *StBs4-2* was strongly associated with PM (Table 6). The two SNPs showed very little LD with each other (*r*^*2*^ = 0.13), whilst solcap_snp_c2_22990 was in LD with solcap_snp_c2_11924 in *FBA* (*r*^*2*^ = 0.35) which is located 900 kbp distal from *StBs4-1*. Both *FBA* and *StBs4* are included in the genome segment on chromosome V which contains the major QTL for plant maturity (see below). The fact that effects on resistance and maturity were detected by different, independent SNP markers suggests that both traits are controlled, at least in part, by different alleles of the same or physically closely linked genes.

The sixth locus PGSC0003DMG400032829 (solcap_snp_c2_35100) on chromosome VII encodes a C3HL domain class transcription factor (*C3HL-TF*) which was not detected by comparative transcript profiling, neither by SuperSAGE nor by RNA-Seq analysis. The potential role of this gene in resistance remains to be elucidated.

The seventh locus PGSC0003DMG400020587 (solcap_snp_c2_47952) on chromosome IX encodes a homolog of *Rpi-vnt1*, a major gene for resistance to *P*. *infestans* that has been cloned and characterized from the wild potato species *S*. *venturii* [65, 127]. This particular gene belongs to a huge cluster of putative resistance genes located between 59.3 and 61.0 Mbp on chromosome IX. The functionally characterized *Ph-3* gene for resistance to *P*. *infestans* from the wild tomato species *S*. *pimpinellifolium* is a member of the same gene family. It is located in the syntenic genomic region on tomato chromosome 9 [67] (Fig 2). The late blight resistance genes *R8* and *R9a* from *S*. *demissum* also map to the tip of chromosome IX [128, 129]. The *S*. *tuberosum* homolog of *Rpi-vnt1* was represented with three differential SNPs in the RNA-Seq results (S7 File). Amplicon sequencing confirmed the association of solcap_snp_c2_47952 with MCR and identified additional associated SNPs (Table 2). In an independent association study in a population of 103 Latin American tetraploid potato cultivars, solcap_snp_c2_56418 (chr09:60182931) which is physically tightly linked with solcap_snp_c2_47952 (chr09:59560440), was associated with quantitative resistance to *P*. *infestans* [130]. The most distal segment of the long arm of potato chromosome IX is evolving as one of several hot spots for pathogen resistance in the Solanaceae, which contains several loci conferring qualitative and quantitative resistance to different pathogens. Taken together, our data strongly suggest that one or more members of the resistance gene families located at the tip of the long arm of chromosome IX directly contribute to quantitative resistance to late blight not confounded by late maturity.

Finally, the eighth locus PGSC0003DMG400018824 on chromosome XII (solcap_snp_c1_3326) was annotated as RNA binding protein 50 (*RBP50*). RNA binding proteins are integral components of ribonucleoprotein complexes and play a central role in RNA processing [109]. This particular gene was detected in RNA-Seq analysis with three differential SNPs.

### Loci associated with plant maturity

Major effects on plant maturity and rAUDPC were detected by eighteen SNPs in the candidate genes *G8T*, *TPARL* and *ATPase* and twelve SolCAP SNPs in nine genes located on chromosome V in the genome segment between 3.0 and 5.5 Mbp (Fig 1, Tables 2 and 6, S5 File, S10 and S11 Files). Genes associated with PM were not exempt from detection in the RNA-Seq experiment because the genotype pools R8 and S8 were constructed based on the evaluation of rAUDPC which was not corrected for plant maturity. This genomic region contains the functionally characterized *StCDF1* locus which controls tuberization under long day conditions [14]. The *StCDF1* locus itself was neither represented on the SolCAP genotyping array nor in the RNA-Seq results. The highest significance and portion of the total variance of PM explained (22% to 35%) (Table 6) was obtained with solcap_snp_c2_50302 in the locus PGSC0003DMG400031262 annotated as methyltransferase or methylase, which mapped 510 kbp proximal to *StCDF1*. This and the other SNPs in this locus did not show strong LD with any other SolCAP SNP physically closer to *StCDF1* and also associated with PM. The effect on PM of this particular methylase gene cannot be explained therefore by LD with *StCDF1*. This suggests that *StCDF1* is not the only gene responsible for the major QTL for plant maturity on potato chromosome V [4, 15–17]. Interestingly, a single SNP in the ATPase candidate gene (ATPase_SNP8491) showed a minor but robust effect on MCR (Table 2), which is in line with earlier observations that the major QTL for resistance to late blight on chromosome V is not completely explained by the maturity QTL [4, 17]. *ATPase* was selected because it was with 30 differential SNPs one of the top sixty candidate genes resulting from RNA-Seq analysis. Other candidates for the observed effects on resistance are the *R1* gene for resistance to *P*. *infestans* [8] and other putative resistance genes located in the same genome segment, such as ‘Bacterial spot disease resistance protein 4’ (solcap_snp_22990) (Fig 1, S11 File) (see above).

Additional small to moderate effects on PM were observed for SNPs in 21 candidate genes and by SolCAP SNPs in at least 60 loci on virtually every chromosome. Most of those were detected by the S and K_2_ association models with the worst fit in the Q-Q plot for PM, indicating many of these loci could be false positives. The trait plant maturity itself might be a major determinant of population structure in European potato, where varieties are principally categorized according to plant maturity as very early, early, mid-early, mid-late and very late. The genomic regions most densely populated with possibly false positive associations with PM (between 40 and 51 Mbp on chromosome III, 6 and 10 Mbp on chromosome IV, 46 and 49 Mbp on chromosome VIII and between 59 and 60 Mbp on chromosome X) might be those that are under human selection for maturity type (Figs 1 and 2). The segments on chromosomes IV and VIII overlap with QTL for plant maturity that were detected in experimental F1 families of diploid and tetraploid potato [4, 15]. Except for the major QTL on chromosome V, the majority of SNPs associated with plant maturity were different from the SNPs associated with resistance, even when located in the same gene. Examples are *arpP1a*, *DNAJ8*, *Plox1* and *CYP71D11*, where the SNPs associated with PM did not show an effect on MCR and vice versa (Table 2). This suggests that the same or different, but physically tightly linked loci influence plant maturity and resistance but with different alleles. This is promising news for the possibility to break the correlation between resistance to *P*. *infestans* and late maturity by choosing the right SNPs for marker-assisted selection.

## Conclusions

Including this paper, the PIN184 population has been intensively genotyped for more than 1000 SNPs at 83 candidate loci and for more than 6000 genome wide SolCAP SNPs [5, 24, 34]. As a result, SNPs at ten loci, *StAOS2* [5, 34], *BCCP* [24], *HMGCR*, *StGP28*, *Plox1*, *CYP71D11*, *Rpi-vnt1*, *TEF1*, *C3HL-TF* and *RBP50* (this paper) showed strong associations (R^2^ > 10%) with MCR. These ten loci are considered most suitable for the application as diagnostic markers in breeding programs. The ten genes encode enzymes functional in the jasmonate and oxilipin pathway (*StAOS2*, *Plox1*), in the biosynthesis of lipids (*BCCP*, biotin carboxyl carrier protein) and secondary terpene metabolites (*HMGCR*, *CYP71D11*), have unknown functions (*StGP28)* or function in pathogen recognition (*Rpi-vnt1*) or transcriptional regulation (*TEF1*, *C3HL-TF*, *RBP50*). They are strong candidates for (i) being directly involved in the control of quantitative resistance to late blight that is not compromised by late plant maturity, (ii) for further functional characterization and (iii) for validation of diagnostic power in different breeding populations and environments.

## Materials and Methods

### Plant material and phenotypic assessment of late blight resistance

Sixteen tetraploid breeding clones (SL, BL) were used for comparative RNA-Seq analysis. SL and BL clones originated from the breeding programs of SaKa Pflanzenzucht (Windeby, Germany) and Böhm-Nordkartoffel Agrarproduktion (Ebstorf, Germany), respectively. They were selected from 76 F1 genotypes (39 SL, 37 BL clones) originated from four crosses between seven tetraploid, heterozygous parents (two were half sib families) based on two criteria: first, the genotype of the SNPs StAOS2-SNP691 and StAOS2-SNP692 at the *StAOS2* locus and second, resistance to late blight evaluated in the field (Table 3). Eight clones (R8) were homozygous or triplex for the haplotype *StAOS2-A*_*691*_*C*_*692*_ associated with increased resistance, whereas the other eight (S8) were homozygous or triplex for the haplotype *StAOS2-G*_*691*_*G*_*692*_ associated with increased susceptibility. In addition, the R8 and S8 plants were selected for contrasting levels of field resistance (Fig 3), which was evaluated in 2009 and 2010 as described previously [4, 5, 23]. The mean value of the relative area under the disease progress curve (rAUDPC) in two years was 0.31 and 0.56 for R8 and S8 plants, respectively. rAUDPC [131] was evaluated as described [5].

A population of 184 tetraploid breeding clones described previously [5] was used for the association analysis. We refer to this population as ‘PIN184’. The PIN184 population has been evaluated in replicated field trials for the ‘area under disease progress curve’ (AUDPC) and for plant maturity (PM). PM was scored from 1 to 9, where 1 indicates very late and 9 very early maturity. From the data for AUDPC and PM the traits rAUDPC and ‘maturity corrected resistance’ (MCR) were calculated as described [5]. As the results for AUDPC and rAUDPC were very similar, we report only results obtained with the rAUDPC data.

### Preparation of samples for RNA-Seq analysis

SL and BL plants were infected in a controlled environment (16h light, 22°C, 8h dark, 20°C) with a mixture of *P*. *infestans* isolates that overcame all known *R* genes. Leaflets of similar size were collected from the 4^th^ and 5^th^ compound leaf just before inoculation (T0), one (T1) and two (T2) days post inoculation, immediately frozen in liquid nitrogen and stored at– 70°C until use. Further details of growing the plants, inoculum preparation, infection procedure and tissue sampling were described in [23]. The infection experiment was repeated five times with different batches of plants and inoculum. Three experiments were selected for sample preparation. One leaflet each of eight quantitative resistant (R8) and susceptible (S8) genotypes (Table 3) were pooled for each infection time point and each of three infection experiments. Total RNA from 18 pooled tissue samples (two genotype pools, three time points, three infection experiments) was extracted, purified and quantified as described [23] and stored at -70°C. Then four RNA samples were generated by pooling equal amounts of total RNA:

1. Sample R8-T0: RNA of the R8 genotype pool before inoculation (T0), pooled from three infection experiments.
2. Sample R8-T1T2: RNA of the R8 genotype pool after inoculation (T1 and T2), pooled from three infection experiments.
3. Sample S8-T0: RNA of the S8 genotype pool before inoculation (T0), pooled from three infection experiments.
4. Sample S8-T1T2: RNA of the S8 genotype pool after inoculation (T1 and T2), pooled from three infection experiments.

### Construction of normalized cDNA libraries and sequencing

Four normalized cDNA libraries were custom synthesized from 2 μg total RNA each of the samples R8-T0, R8-T1T2, S8-T0 and S8-T1T2 by GenXPro GmbH (Frankfurt, Germany), pooled and custom sequenced by Solexa/Illumina technology on an Illumina GAII instrument (Illumina, Inc., USA). In brief, polyadenylated transcripts were captured using biotinylated Oligo-dT-primers (Dynal, Thermofisher). cDNA was generated by first- and second strand synthesis using Superscript II (Invitrogen) as reverse transcriptase. The cDNA was hereafter normalized [132] using double-strand specific nuclease (Evrogen). The normalized cDNA was fragmented to an average size of 350 bp and p5 and p7 adapters for Illumina sequencing were ligated, followed by PCR with 12 cycles.

### SNP genotyping using the 8.3k SolCAP potato SNP array

The PIN184 population was genotyped for 8303 SNPs using the 8.3k SolCAP potato genotyping array [29]. Custom genotyping was performed by the Department of Genomics, Life & Brain Center Bonn (Germany), on an Illumina iScan system using the Infinium assay. Genotypes *AAAA*, *AAAB*, *AABB*, *ABBB* or *BBBB* were called for 6286 SNPs and each individual using FitTetra software [133]. For association analysis genotypes were converted in the numerical values 0 (*BBBB*), 1 (*ABBB*), 2 (*AABB*), 3 (*AAAB*) and 4 (*AAAA*).

### SNP genotyping by amplicon sequencing

Plants selected for RNA-Seq analysis were genotyped for the SNP markers StAOS2-SNP691 and StAOS2-SNP692 by amplicon sequencing as described [5]. Amplicons for the candidate genes selected from the jasmonate pathway and the RNA-seq analysis were generated as follows: Primers were designed based on the potato (DM, *S*. *phureja*) genome sequence [33] using NCBI Primer BLAST (http://www.ncbi.nlm.nih.gov/tools/primer-blast/) in such a way that approximately 500–1000 base pair fragments were amplified by PCR (polymerase chain reaction). Primer and amplicon sequences, annealing temperatures, positions and alleles of SNPs are shown in S1 File. Standard PCR reactions were performed in 25 μl PCR buffer (Ampliqon A/S, Odense Denmark) containing 1.5–2.5 mM MgCl_2_, 0.2 mM dNTPs, 0.25–0.50 μM of each primer, 50 ng template DNA and 1 U Taq polymerase (Ampliqon). Cycling conditions were: 1min initial denaturation at 94°C, then 35 cycles of denaturation for 30sec at 93°C, annealing for 45sec at T_a_ (S1 File), elongation for 1min at 72°C, final elongation for 10min at 72°C. PCR products were examined for uniformity and band singularity on agarose gels and purified with illustra™ ExoStar™ (GE Healthcare Europe, Freiburg, Germany). Amplicons were sequenced at the Max-Planck-Genome-Center Cologne using the dideoxy chain-termination method, an ABI PRISM Dye Terminator Cycle Sequencing Ready Reaction Kit and an ABI PRISM 3730 automated DNA Sequencer (Applied Biosystems, Weiterstadt, Germany). SNP and insertion-deletion (indel) detection and scoring including SNP allele dosage was performed as described [5]. For association analysis SNP genotypes were converted in numerical values 0, 1, 2, 3 and 4 as above.

### RNA-Seq data analysis

A total of 133.2 million paired-end reads from the R8 genotype pool and 116.2 million paired-end reads from the S8 genotype pool were mapped to the *S phureja* reference genome sequence (version 4.03) using Tophat v2 with the parameter settings ‘max-insertion-length’ = 12, ‘max-deletion-length’ = 12, g = 1, m = 1, ‘read-gap-length’ = 12, ‘read-edit-dist’ = 12, ‘read-mismatches’ = 12, ‘read-realign-edit-dist’ = 0, ‘no-coverage-search’, ‘segment-mismatches’ = 3 [134]. From these, 64 and 49,9 million reads, respectively, were uniquely mapped to the reference genome and further analysed. In order to detect polymorphisms between the R8 and S8 genotype pools, we first used default settings in Picard (http://broadinstitute.github.io/picard/) to remove duplicated reads, and in GATK (Genome Analysis Toolkit) to realign indels and call variants between samples [135]. This analysis resulted in an initial set of 4 380 958 variants. For further analysis we considered a total of 566 806 bi-allelic SNPs located in annotated exons, which had a phred quality score greater than 30 and were covered by at least ten reads in each genotype pool. For each variant we determined significant differences in allele frequencies between the R8 and S8 genotype pools using Benjamini and Hochberg adjusted p values (q) from a Fisher’s exact test on the number of reads supporting each allele in each genotype pool.

### Association analysis

The adjusted entry means for MCR, rAUDPC and PM have been calculated previously [5] and were used for all association models. Kinship and population structure were analyzed based on the genotypes of 241 SolCAP SNPs that were selected for equal distribution on all chromosomes, a minor allele frequency (MAF) ≥ 10% and having no missing data (S2 File). Association analysis was performed with four different models. The simplest model without correction for population structure (model S) was a linear regression model that tests association of each marker with the trait, and assumes that genotypes are independent. This model can be written as phenotype = marker + error, where marker is a fixed effect. Model K_1_ is a model that corrects for kinship between cultivars. We used a linear mixed effects model on each SNP marker (random effects are underlined):

y=μ+Mα+Gβ+e,

where y is a vector with trait values, μ is the mean effect, M is a vector containing the SNP dosage of each cultivar, taking a value between 0 and 4, and α is the fixed additive effect of the SNP. G is a factor that identifies the cultivar corresponding to each observation in y. Vector β is a vector of random polygenic background effects, such that Var(β) = σ^2^_G_K, and K is a n x n kinship matrix calculated as a function of the accumulated euclidean distance between marker genotypes, the latter expressed on a scale from 0 to 4 [136]. Finally e is the vector of residuals, such that Var(e) = σ^2^_e_]. Models S and K_1_ were fitted using Genstat version 17 [137]. The third model K_2_ was similar to K_1_, except that kinship between pairs of genotypes was calculated according to [138], using SPAGeDi software [139]. Negative kinship values between genotypes were automatically set to zero. The fourth model K_2_Q included the kinship structuring matrix from K_2_ as well as an additional fixed correction for population structure [24]. Groups were identified by the Bayesian clustering approach implemented in the STRUCTURE software [140]. Burn-in time and iteration number were set to 100000 with ten repetitions, testing the probability of up to twenty subpopulations. An admixture model with correlated allele frequencies was used. The results were uploaded to the software STRUCTURE HARVESTER [141] at http://taylor0.biology.ucla.edu/structureHarvester/ and the most likely number of subpopulations K = 2 was determined with the Evanno method [142]. The K_2_Q model has the form: y = Xb + Qv + u + e, where y is the vector of the phenotypic values, X is the vector of SNP marker genotypes, b is the vector of marker fixed effects to be estimated, Q is population structure (derived from STRUCTURE analysis), v is a vector of fixed effects due to population structure, u is the vector of random effects due to the K matrix and e is the vector of residuals. Models K_2_ and K_2_Q were fitted using TASSEL version 2.1 [143], which can accommodate the five possible genotypes (AAAA, AAAB, AABB, ABBB and BBBB) for a bi-allelic marker of tetraploid potato. For SNPs in candidate genes that were selected based on biological function and/or differential expression, marker trait associations with p < 10^−2^ (- Log10(P) = 2) are reported. In the genome wide scan with SolCAP SNPs, a correction for multiple testing was used following the method of Li and Ji [144], implemented in procedure QTHRESHOLD in Genstat. This procedure produced a threshold of–log10(P) = 4.54 for testing a single marker with a genome wide error type I α = 0.05. We report marker-trait associations with p < 10^−4^ (–log10(P) = 4.00). Associations of SNPs with minor allele frequency (MAF) less than 1% and more than 30% missing data were not considered further.

Linkage disequilibrium (LD) and LD decay were analysed as described [32], based on 5600 SolCAP SNPs, except that LD and LD decay were calculated per chromosome using the physical positions of the SolCAP SNPs. P values were corrected for multiple testing using the R package ‘qvalue’ according to [145].

## Supporting Information

S1 File Amplicon sequences, SNP positions on the potato physical map (version 4.03), SNP alleles, primers and annealing temperatures of candidate genes. (DOCX)

S2 File SolCAP SNPs selected for kinship and population structure analysis. (XLSX)

S3 File Information on the candidate loci tested for association with rAUDPC, PM and MCR in the PIN184 population in this paper and in previous papers. (XLSX)

S4 File SNPs in candidate genes associated with MCR (p < 0.01) in any of the four association models K_1_, K_2_, K_2_Q and S. (XLSX)

S5 File SNPs in candidate genes associated with rAUDPC (p < 0.01) in any of the four association models K_1_, K_2_, K_2_Q and S. (XLSX)

S6 File SNPs in candidate genes associated with PM (p < 0.01) in any of the four association models K_1_, K_2_, K_2_Q and S. (XLSX)

S7 File Information on 42 688 SNPs with differential allele frequency (q < 0.01) between R8 and S8 genotype pools. (XLSX)

S8 File Positions in the potato genome and corresponding references of DNA markers linked with *P*. *infestans* QRL in potato and tomato. (XLSX)

S9 File SolCAP SNPs associated with MCR (p < 0.0001) in any of the four association models K_1_, K_2_, K_2_Q and S. (XLSX)

S10 File SolCAP SNPs associated with rAUDPC (p < 0.0001) in any of the four association models K_1_, K_2_, K_2_Q and S. (XLSX)

S11 File SolCAP SNPs associated with PM (p < 0.0001) in any of the four association models K_1_, K_2_, K_2_Q and S. (XLSX)

S12 File Q-Q plots of the p-values of SolCAP SNPs obtained with association models K_1_, K_2_, K_2_Q and S for MCR, rAUDPC and PM. (JPG)

S13 File Distribution of the SNPs with different allele frequencies in genotype pools R8 and S8 on the potato physical map (XLSX)
