# Supplementary material for: Targeted and Untargeted Approaches Unravel Novel Candidate Genes and Diagnostic SNPs for Quantitative Resistance of the Potato (Solanum tuberosum L.) to Phytophthora infestans Causing the Late Blight Disease
Source: PLoS One. 2016 Jun 9;11(6):e0156254. doi: 10.1371/journal.pone.0156254 (PMC4900573; doi:10.1371/journal.pone.0156254)
Supplement: S1 File — (DOCX) [file pone.0156254.s001.docx]

Supplemental file S1: Amplicon sequences, SNP positions on the potato pseudomolecules v4.03 (<http://potato.plantbiology.msu.edu/cgi-bin/gbrowse/potato/>), SNP alleles, primers and annealing temperatures of candidate genes. Primers are highlighted green; the primer used for amplicon sequencing is underlined; designation as ‘forward’ and ‘reverse’ primer is arbitrarily and does not necessarily correspond to the direction of transcription. The SNPs scored in the PIN184 population are highlighted blue, SNPs with differential allele frequency in RNA-Seq analysis are highlighted yellow. SNPs are numbered with the last three to five digits of their position (v4.03) in the reference genome.

*Ank*, ankyrin repeat-containing protein, PGSC0003DMG400019975

>Chr01:560094..559419

Tgtggtagcgattctcaaaggcgtatcagaagagcaagtgggagcaaatctttctcatggtctcactctgcaaat

559.993

atctctgatatggatgactacaactc[t/c]gatgacagaatttatgctttgtgagactgacattatgcaatgtt

.943 .938(indel) .911 .891

cgatc[t/c]aact[g/a]tgatatggatgactacaacacgagaa[g/a]actgaaagtatagtcatac[g/a]c

.874 .867 .866

tgttgaaccctgtgc[a/g]tgtcac[c/t][g/t]agaaagtgaaagtgatgttgagtcatgatacctacacag

.793

acatcaggtactcattttcaacaccatgcacga[a/g]aacttccctttttttcgctcatagtatgagttatgat

.689

gaattgacaaaatccagagatctgctagttagttagttgtataggtttcgcgttgcttcagtcctt[c/t]gaga

.660 .622

tgtccatttacacaagtagtttgt[t/c]actgaacactttttgtggcatgtacggtgtacccctt[c/t]gatgtataatcataatctgaggtttgtagtttgtacataatagtgttagggactatgaaatccctataaaaatcttgtaagttgttggatggaacattgcaaaagaaatctacataaaaagccttctgaatatgtactttcattatgtttatgttgtcccaggcctcggagcctcgttcgcctttggtacttcagttagacaa

MF18_Ank-forward: 5’tgtggtagcgattctcaaagg 3’

MF18_Ank-reverse: 5’ttgtctaactgaagtaccaaag 3’

Ta: 55°C

*arpP1a*, acidic ribosomal protein P1a, PGSC0003DMG400032190

>Chr01:1651576..1651929

Gctactggattgggatgatcggattgtcctcgttatgaaagtttaacagatcatccaaaccataaaacgacataa

651.673

Ttggagtcggtaataagtaaca[t/g]aactgatgttcataaaccataattatgtcaaggttacatttaaca

.719 .750 .781

[g/a]tgAcaagcagatcaaaaatctccttaacaa[g/c]ggtgctataaccaagtctcatcaaactttt[a/t]

.791

aaaaagaac[a/c]aaaaatcgtactgggagtagctcctaatcaaacagactgaatcccatatcgtcatcacttt

.900

cttccttgggctcttcctgttcgtcattacaacaaaagcaaacaaaa[a/g]ttaaagaggtttgcaatgacaatgaacta

MF19_arpP1a-forward: 5’gctactggattgggatgatc 3’

MF19_arpP1a-reverse: 5’tagttcattgtcattgcaaacc 3’

Ta: 55°C

*LoxH1*, 13-lipoxygenase (X96405), PGSC0003DMG400032155

>chr01:2143735..2144507

Gcatcctcatttcagatacacaatggagataaatgccttagctagagaagcacttattaatgctaatggtgttat

14.3831 .3841 .3846 .3861

tgagagttcatttttcccagg[c/g]aagtatgca[a/g]ttga[a/g]ttgagttctattgc[t/c]tatggtg

.3885 .3888

ctgaatggagatttga[c/t]ca[a/g]gaggcactcccacaaaaccttattagtaggtaataattcactaatct

.3937 .3952 .3999

t[t/c]tattattctaattt[g/a]attcggtgtttggtatctgttttgggaccctactaatctaattcat[g/a

.4000 .4011 .4026 .4029 .4046 .4048

][t/a]aaaaagatgc[c/t]actttggagtaaaa[c/t]gc[c/t]cccaattaaatgtgac[t/a]c[g/a]g

.4052 .4059 .4077

tt[a/c]aaaaaa[g/a]gggtagatctcgagaca[t/c]gtggggctcattttagggttagaaaatcctatattggaggtaaaatgctcccaaccaaaggtgcttccatttgcaaaggtcgaccttgagacctctaattgttaagttta

.4200 indel(G)

ttttttattggg|caatagttctgaaaagacaaataactaatcttttctttcatctcgggagaaatacgattgtgatgagctataatgtattcacaagtaagttctttaccctttagacaaccttgattttgtaaatcaagatttattggtttgtacattggtattacacaaacaggagattcaacatctactatatatacacataaaaaataactttgaccttatatatagtataatttttcgacgaaaaggactcagacgatcccctacttctactaacatgttattttgtgcaaaatttacaggggattggcagtgga

LoxH1-forward: 5’gcatcctcatttcagatacacaat 3‘

LoxH1-reverse: 5’tccactgccaatcccct 3‘

Ta: 59°C

*Pen1*, penetration 1 (AY616763), PGSC0003DMG400021331

>chr01:2809648..2808396

Atgggaga[c/t]accggtgg[a/t]gtcaacctagacaagtttttcgaagacgtagaagctattaaagacgagc

2809.532 .514

tgaaaaacctcgaaaaaatctatgctcagcttcagagctctaatgaaaa[g/a]agtaagacccttcacaa[c/t

.511 .493 .487 .465 .460

]gc[g/a]aaagctgtgaaggatct[t/c]aggtc[t/c]aaaatggatgatgatgtttcc[c/a]tggc[c/t]

.397 .394

ttgaaaaaggccaagtttattaaagttcgtttggaagcgttggatagatccaacgcttccaa[c/t]ag[a/g]a

.373

gcctccccgagtgtggccc[g/a]gggagctcctctgataggacgaggacgtctgttgtgaatggattgaggaag

.313 .283 .270

aaacttca[t/a]gaatctatgaatcagtttaatgagctgag[a/g]caaaagatggcg[t/a]cggaatataga

.207

gaaacggttcaaagaaggtattatactgttactggagaaaatcctgatgaa[a/g]cagttcttgatacactcat

.147 .141 .137 .136

atctacaggtaaattttactcccttgtctcattgtatatt[c/t]gaatt[c/a]aca[c/t][a/g]gaaaatt

.124 .111 .102

agat[t/a]gtctaaacctcg[a/c]aaagtaga[c/t]ggagggagtacaaatttaaagggcacgattgtggtg

.064 .063

g[g/c][g/t/c]tggtaagtattcctccatctcagacaagaaatttggttaagggtattcaagatttaagttttgatctatatacttaatattagtttttatatgcacagtctaattctccgacaagatgatcctccatcgtatgcagttccatccttaatcagaggtcttgaaatcaaacctaggggaatagagtagtctttagtatggagtggtttacctctaaatgggactttttgatgtaaattcggattaatcaagctccaaaacgaatatctgacattatatatatcaaaagagtaaaactcatccaaatttccaagtgacatatcaaacaatacttatttcatgtgtctagaccgaataggttgatggatcgttcatcgttacgagtcataacagaacaaacaacttgtttttgtgtcccacaggtcaaagtgagacattcttacaaaaggcaatacaagaacaaggaagaggacaagtgatggacacaataatggagattcaagaaaggcatgaagctgtaaaggaaatagagaggaatttgaaagaacttcatcaagttttcttggacatggctgttttagtggaatctcaaggtgaacaacttgatgatattgagagccaagtgaatagggctaattcatatgttagaggaggtgctcaacaattacaa

Pen1-forward: 5’atgggagataccggtggtgtc 3’

Pen1-reverse: 5’ttgtaattgttgagcacctcctcctc 3’

Ta: 61°C

*DIR1*, defective in induced resistance 1 protein, lipid binding protein, PGSC0003DMG400011323

>Chr01:58072131..58072704

Gtcctcagcttataaattcaacaatttaacttgcaaactccacatacactttacttgtaaacatgcctatgaaaggaagcaaagtgggagttttgggtatggtagttctaggactactacttatagtagaatttacaagtggattgagca

58072.288 .297 .308

tttgtaa[g/c]atgaatga[c/t]gatggtttga[c/g]atcatgtaagccatcagtaacacagccaaatccag

.346 .347 .392

tg[a/g][g/a]gccatctgcttcttgctgtgaagccttgtcagctgcagacttgc[g/a]gtgcttgtgttcttataggaattcattcgtattgccctctcttggaattgatcctgaacttgcattggctcttcctactaaatgcaatc

.484

tc[a/t]cttctcctcctaattgttaagaaaaactaaatagaggtcttccatttcttccctagtactacacccct

.597 .616

agctagcttcttaattaaactactttaattttgttgttgtaatt[c/t]aatttatgtttgattttg[g/a]gac

.636 .638 .664 .678

ctctatccatttgaaa[a/t]g[g/a]agtgttaaagacttcgtttgttttc[g/a]ttaagattaatat[g/a]tttgaatctatatgattaagatgtcg

MF22_DIR1-forward: 5’gtcctcagcttataaattcaac 3’

MF22_DIR1-reverse: 5’cgacatcttaatcatatagattc 3’

Ta: 55°C

*TMP14*, tylakoid membrane phosphoprotein 14 kDa, chloroplast, PGSC0003DMG400000204

>Chr01:73019320..73019803

Tgtaaactttcaagaatagacagaaataatacaaagaaaggatctttttagaaaaatactactaacccaaagcaccatatgtctaatttaattgaattgggttctcctttcccccctaattacaagtgtaacgttaataggtagtccccatcaataacattaatggtctaagaaacattataacaaggaccaataagaaagaggggtaccagcaaatttgagaga

73019.554 .561 .599

agatgggaa[g/t]gcagta[g/a]cagaaaggcggggtggaaggtaaggcaaggcggagca[g/c]cgagctgg

.609 .659

g[g/a]cggctttggtggtggaggaaagtgggaaacgagaggcaaagacggcagt[a/c]gccgccattgaagtagaagctgctgccatttggccaattcttggaatcttggactgagctgtgtatgcttggattcttggcctctctttctttcttgtgtatctcaaatatatcttttttagtttcttgatatccccaaaaagt

TM10_TMP14-forward: 5’gtaaactttcaagaatagacag 3’

TM10_TMP14-reverse: 5’gtactttttggggatatcaaga 3’

Ta: 55°C

*EIF3*, eukaryotic translation initiation factor 3, subunit D-like, PGSC0003DMG400029694

>Chr02:33876714..33877628 3387.6781

Cactcaaagaataagctgaattgatatcatccttcacatcaggcaatggttcttgcgaagtttcatg[c/a]aca

.6802 .6850

gagagcaaatccagctg[t/c]gacccatcacgcttgtcgaaaaacaacttattcccaacacgctgaac[a/c]a

.6877 .6916

caatatcccatgagtaaacagacct[g/a]ggagcacacattaaagtagacaagattgtatctgtggc[g/a]aa

.6931 .6964

aacagtagcttt[a/g]tcctcattagccaaacggcggataacaggatc[a/g]tcagtagtagtaattttgaag

.6988 .7015 .7027 .7042

aa[a/g]ttcctgttcttgaacctctccagacg[g/a]cgttcgttctt[t/c]ggagtaatacggtc[g/a]ta

.7047

gg[a/t]ccggtcgtaaaactcaaggccaccgcagattagcaagtcttcaggctctggaacggagaacgaaagctttgagaaggtagaaaacgggatctgatcaagcatgttccattcaggctgaatatcaactgatgacttgaacaccgcagattctcttctctggccagcattggtgcctgaacgattgagattatacaatctgtcccgacgggctctttctttctcttgctcacgttttttggcctcgacttcctcgtcgcgccgctgtggaagctgagaacggttattatgaggattgaaccgccacctagggttgaacttcggacgatgttgtccatggtgatgggatttagcggcagtatcaacgagacggaaagatgaatcttcgtcggcggtgagtgtagcgaaggagtcgtcaccggagaagtcgaaagcggaatcagtagggttttgcttggagttggatccaggtcggtttgagagagagcgagtccaatcagcgatccgaccgagcttgtcggagcgggagaaaggggcaaaaggggcgttagagatttggtttgcaacggagacggaagaatccg

TM11_EIF3-forward: 5’cactcaaagaataagctgaatt 3’

TM11_EIF3-reverse: 5’cggattcttccgtctccg 3’

Ta: 57°C

*AOC*, allene oxide cyclase (AY135641), PGSC0003DMG401012679

>chr02:40953060..40953477

Ctgcagacctaaagaagagaattggaataacagcaggactctgcattctgatcaagcacgaagaagagaagaaag

40953.152

gagatcgctatgaagct[g/a]tttacagcttctacttcggcgattacggtcaaatcgccgttcagggatcgtac

.222 .247

ttaacctatgaagaca[c/g]ttacctcgccgtcaccggcggatc[t/c]ggcatatttgctggggtttccggtc

.286 .295 .318

aagtaaaattgca[t/g]caactcat[t/c]ttccctttcaagctattctaca[t/c]tttttacttgaagggga

.391

tccccgatctgccatctgagttgttgtgtacggcggttcctccgtcgccgacggt[a/g]gagccaacacctgaa

.442

gctaaagcttgtgaggatggggccgcactgaaaaa[t/c]tacactaattaagtgggggtgttgatgtgcaaatc

AOC-forward: 5’ctgcagacctaaagaagagaattg 3’

AOC-reverse: 5’gatttgcacatcaacacccccact 3’

Ta: 59°C

*PQ-lr*, PQ-loop repeat family protein; lysosomal cystine transporter family protein, PGSC0003DMG400013431

>Chr03:254350..254840

Aagagcacaaggccaggcatggcatacaacattgtttgttcatatttacaaattcataaaatagaaagtttacta

Gccacagtttcttttctttaattatgtatctgtcttcacttcagaataactgtgtacactgtatatctatcta

254.498 .546 .551

[t/c]gcttctatcccaagcaagttggattccttatattgctccatttaggg[t/c]catc[c/t]attatagaa

.563 .600 .603

ta[t/c]tatgtgtacctaatcaaaatatacatttgctatctt[a/c]ca[g/c]tcgagacagattttccaatg

.625 .671

a[a/g]tgatgcattatacatcttccttgttttcgtgatcagaagttttaa[g/a]taatggcctcttgcctatc

.709 .727

acatcaacatctcgagaa[g/c]tcccaatcttccaagca[g/a]gatagatcacataatgttgcaaaatgaaga

.758 .768 .775

[g/a]aatgtcaaa[g/a]aatatc[g/a]acacctgcaacattcatccaccaaaccaaatagtagtcagagctattctcgtgatactagcaata

MF1_PQ-Ir-forward: 5’aagagcacaaggccaggcat 3’

MF1_PQ-Ir-reverse: 5’tattgctagtatcacgagaata 3’

Ta:55°C

*StTinI*, TMV-induced protein I, PGSC0003DMG400016749

>Chr03:34073899..34074546

Agcactataagctcaaattaaattacatcaacaacatgataggactgagaatcgtaaatagagaattgctcatct

3407.4009

gacttacggccacagttgttcttaatagccgttag[c/t]ccatctggaaaaacatcagcaccaagccaactctc

.4045 .4062 .4098

[g/t]acggtaaacaacttct[g/t]gttgcatcgtttgtgctctgctgtagaagtaactt[c/g]aacgtaatt

.4111 .4129 .4141 .4159

aca[a/g]taccatccagggtgttg[c/g]cctgtaccatc[a/t]gaagtcaagttcatttt[a/g]cagattg

.4210 .4230

gaccattcaaacatggaccacgaccagtaaacatatccaaatt[g/a]tccctttcaaagtagtcgt[g/a]acc

.4267 .4292

ttcgcccataagcccaccccacgcttgtaggtt[t/g]ttgattctaagtccatatccatcc[g/a]catcgtaa

.4306 .4315 .4334 .4345

agagt[t/c]aagataat[t/g]tttgagtcagttccagac[g/c]caaagaattg[c/t]ccagttcgaatgta

.4423

aactgagtatacacaatttaattcctgcattaatttcccgtcaattcaacaattgcaccaaaa[t/a]aataacacaacggtattttggtattgtaagtaagaattttggattatgaatgtatacatgacattagaattaggcgcataatttagtaattcaccaatagtgtagaagttgacttacagagct

TM14_StTinI-forward: 5’agcactataagctcaaattaaat 3’

TM14_StTinI-reverse: 5’agctctgtaagtcaacttctac 3’

Ta: 57°C

*Smp24*, 24 kDa seed maturation protein, PGSC0003DMG400019959

>Chr03:44501775..44501309

Tcaaagttgtgtttccattatctgacttataatttaaagcaacactcacttgcatattgatgttttacgtcacat

44501.674 .672 .670 .665

gatgactgccaaaatcttacagcagt[t/c]t[t/c]t[c/t]aaaa[t/a]ttaacaaaaaagaaaaccatatc

.633 .616

tggaactc[a/g]aaactaccacaaaaac[g/c]ccaagcctgtttagtaataatcaagcaaaacaagtaaacaaagggttttctaccataactgaatcacaacaaccagaggaacctaaagggtgccacctattcatgtctataagaac

.434

tttttgtctttcagtggacctcttggaattttgcttagaaccttgacgttaaaaacagcatactg[c/t]ttttt

.413

gatctctgcctcagc[g/c]ttctcagcaaaagcatcaacctggtcctcaaatttctcataaagtacagggacggtatggagcaatacaaagcctgtttttgcagatagaagagatcacacgca

MF4_smp24-forward: 5’tcaaagttgtgtttccattatct 3’

MF4_smp24-reverse: 5’tgcgtgtgatctcttctatct 3’

Ta: 57°C

*KT-InvInh*, Kunitz-type tuber invertase inhibitor, PGSC0003DMG400010146

>Chr03:49449411..49448248

TCAATAGAATTCCTTACCCGAATTTGATACTTTACCTGCATGTTATTCAGTGTAACACATATTATGTATATATTTTTGTTGATCGTTAAAAAAAAAAAAAAAAGTGTAAAGAGTGACTTAATATTGAAGCTTGATATTAGGTATTTAAAATGAATAAAACTTATTTGAGGTGTCTAAGGCGGTAGTAGGTATTATTTTTTGTTTGCCAAAAATGCCCTTAGCAGCTCTATAAATTGGACATAAACTCATAGCAAGCAAAACACACAAAGAAAGATGAAGTCGATTAATATTTTGAGTTTCCTTTTGCTTTCAAGTACCCTCTCTTTGGTTGCCTTTGCTCGATCTTTCACTTCTGAGAATCCAATTGTCCTCCCC

.9024Indel(+gatgat)(114).9018 .8985 .8981

ACAACTTGTCAT|GATAAT[C/A]TTGTACTCCCTGAAGTTTATGACCAAGATGGC[A/G]ATC[C/T]GCTGAG

.8943 .8934(206).8933(207)

GATTGGTGAGAGGTACATTATTAAGAATCCT[C/T]TCCTCGGG[C/G][C/G]CGGAGCCGTATACTTGAACAA

.8878(261) .8869 .8866(273).8864.8861

TATTGGAAACCTTCAATGCCCAAACGCCGTGTT[A/G]CAGCACAT[G/A]TC[G/A]A[T/C]TC[C/G]CCAA

.8853 .8847 .8836.8832(307).8831 .8826(313)

TTT[T/G]TGGGA[A/G]AAGGCACGCC[C/G]GTC[A/G][T/A]GTTC[G/A]TTCGTAAGTCGGAGTCGGAT

.8805(334) .8787(352) 8777(362) .8748(391)

[G/T]ATGGTGATGTGGTGCGT[C/G]TAATGACTG[C/G]TGTTTATATCAAGTTCTTTGTTAAAACA[A/T]C

.8744(395).8743(396) .8709(430)

AA[A/G][G/A]TTGTGTGTTGACGAAACTGTTTGGAAAGTTAAT[A/G/C]ATGAACAGTTGGTGGTAACTGGT

.8638(501)

GGTAACGTAGGAAATGAAAACGACATCTTCAAGATCAAGAAAACTGA[C/T/A]TTGGTGATACGAGGTATGAAAAATGTATACAAGTTACTGCATTGTCCCTCTCATCTTCAGTGCAAAAATATCGGCAGCAACTTTAAAAATGGATATCCTCGTCTGGTGACTGTCGATGACGATAAGGACTTTATTCCATTTGTGTTCATCAAGGCGTAGAATGCTAATTAGCTGGCTAGCTTGCAGCTTTTTTAAATAAAGTCGATATATCCTTCTATCGCTCCATGTAATTTAATGTATGCTTATCAATAAATAAACAAGCTAGCAATTATCCTATTACCTTACCTTACCTTCTTCATTCAAAAATACATAAATTTCTTTAGTTTGTGGTTTCTTTTTAAGTTAGGGTTACTTTTGTATTTGTTGTTAATCAGGTCTAGATATCGTTAC

For primers and PCR conditions see Fischer et al. (2013)BMC Plant Biology 13:113.Numbers in parenthesis are the SNP positions counted from the ATG codon with A at position 1 according to Fischer et al. (2013).

*SPI*, Miraculin, serine protease inhibitor, PGSC0003DMG400010170

>chr03:49840059..49839498

Ctcaaggatacattaaggtagcaatacttttacaacgatagaggctacacatcacacatgcgtagctttatttag

49839.983.981 .962 .951

a[g/c]a[a/g]tacaagtaggtaggtacg[t/c]acgtacaaac[a/g]tgatgaaaataatacagtgaaaagg

.923

a[t/c]cgatacatattatttggtcgaaattaaccaaattatacaatcgatttaattccaatgttagtatcctta

.804

atgaagacaagattgaaggtattgcctgttcctatggccaatcgccttcc[g/t]gcagtatagtcgatgccaat

.770 .768 .737 .736 .728

gtcagtgcagatg[g/t]g[c/t]tctccataaggacaaaacatcaacttatac[a/c][t/a]gtaactt[c/t

.724 .692 .685 .678 .677

]cag[t/c]tttcttaatttgaaaccatgaggccacattc[t/a]taacat[g/t]tccagc[c/t][t/a]ccc

.669 .666 .665 .647 .622

Catt[g/t]gt[g/t][t/g]ccaagtacattggcata[g/t]catgtcctgggaaaccttctactt[g/c]cca

.601 .599

Cactgtgttgttacaaa[a/t]c[c/t]cacttggattgtcaatatagaactcaatgttaacatcgctcgattca

Gttatttctacttgtttcgctgcctttggtttgaaaatcactggccaacctctg

TM12_SPI-forward: 5’ctcaaggatacattaaggtag 3’

TM12_SPI-reverse: 5’cagaggttggccagtgattt 3’

Ta: 57°C

| *DnaJ8*, Heat shock protein binding protein 8, PGSC0003DMG400014210 |
| --- |
| >chr03:57386377..57385945  57386351  Ctgctatgggaatgatgggaagtatt[g/a]ggggttgtggagcagcttctgcatctctgtttcgattgaggaac  57386.280 .277  tcagcgaagaagaagaccagaaacga[c/t]aa[a/c]aatggatttagggtttcatgtgtttattcatcttctg  .232 .172  ctgttgc[t/c]gatccgtataagaccttgaagattcaacctggtgcatctgaatctgaagttagaaaggc[c/a  .163 .136 .132 .121 indel  ]tttagaca[g/a]cttgctcttaaggtacgcacgccatt[g/c]tta[t/a]ctgctttaaa\|cctggaaattt  .101  tgaatttat[t/g]ttcattaaaaaaaaagaccgttttggtatttttatatgcgtttaattgaattttaagttattaatatgatgtattttattggattacagtatcacccggacgtatgcagaggaaacaattgtggtgttcaatttcaccaaatcaatgaagcttacg  DNAJ8-forward: 5’ctgctatgggaatgatggga 3‘  DNAJ8-reverse: 5’cgtaagcttcattgatttggtg 3‘  Ta: 59°C |

*LoxH3*, 13-lipoxygenase (X96406), PGSC0003DMG400022894

>chr03:61549581..61550570

Ggagagtcgtgtggagaagccaaatccgacgtatgttccgagagatgaacaatttgaggagtctaaaatgaatac

615.49682

attctcaacttccaggcttaaagcag[t/a]gctccataacttaattccatctctaatggccagcatttcttcta

.49763 .49791

acaatcacgatttcaaaggattttcagatatcgata[g/a]cctttatagcaaagggctacttttgaa[g/a]

.49792

[c/t]ttggtcttcaggacgaagtcttaaaaaagcttccattgcctaaggttgttagcagtatcaaagaaggagatctgctcaaatacgacacgccaaagatactatcaagtaattagtccctaacctgttgctaaataggattaatgac

.49956 .49962.49963 .49974

tgattaaaacctattgtg[c/a]aattc[t/g][t/a]ttaactagtg[g/a]tgaatgcttaattaacaaat

.49995 .50004

[g/a]tcaatatt[g/t]tgtacattgcagaggacaaatttgcctggttacgagatgatgaatttgctcgacaa

.50061 .50069 .50090 .50100 .50103.50105

[g/a]caatagc[a/t]ggagtgaacccggtatccat[c/t]gaaaaactt[c/g]ag[g/t]t[t/c]tttcca

.50129

ccagtaagcaagcttaa[t/a]cctgaaatctatggccctcaagaatccgccctcaaggaggcgcacattcttgg

.50189 .50227

tcatct[a/c]aatggcatgactgttcaagaggtaattaaacacaatt[t/g]gatttcacttatacgaacgatacaaattattttacattatcagtgtattttaacttactgtcttactaattaaaaatgttcatttcctaatgtgacaggctttggatgcaaataagcttttcattgtggatcatcatgatgtataccttccatttctcgatcggattaatgcacttgatggccgcaaagcatatgcaacacgcaccatttttttcttgtctgatgttggcacccttaagcccattgccattgaacttagcctcccccaaactggtccaagttcgcgatccaaacgtgttgtcacaccacctgtttgtgccactggtaactggacgtggcagat

LoxH3-forward: 5’ggagagtcgtgtggagaagc 3’

LoxH3-reverse: 5’atctgccacgtccagttacc 3’

Ta: 59°C

D4H, desacetoxyvindoline 4-hydroxylase, PGSC0003DMG400029517

>Chr04:2626378..2625525 .6355 .6328 .6320

Aacacaccgggatctgtatgtgg[c/t]gtcagccctttcgtcaactccggctg[t/a]ggacaat[a/g]agga

.6250

tagtaatgtgcagccataactcttccatccaaacaagacaattccttcaatctatccttttccac[t/c]cctaa

.6191

cccttcacacaataaacccatcaattcttccccaattttcaccacttccttat[c/t]ccactcaacaatcgcct

.6151 .6140 .6139 .6130 .6128

ccctacacacctccggcacata[c/t]tcccaatccg[a/g][c/g]ggagtagg[c/g]g[t/c]caacctcac

.6085

ctgcaacgtatccctccagcttgcagctttcga[g/a]ttgtacaaatcaaaattcgtagaatacgctgctcca

.6048 .5992 .5989

[c/t]gagtaatatccctactataatacttcatcttcaattcatcattctgttcgttaaa[c/t]ga[c/t]tta

.5977 .5958 .5926

atcgaacc[t/a]acaattctgtttatagcg[t/c]tgaccggaacagaatgattgataatctggaa[g/a]aac

.5893 .5884 .5860

cctaagctcgttgaagcacgatgaacttg[t/g]tcaacgac[c/t]tttcccgaggagcagagaagtc[g/a]

.5857 .5818 .5797

ac[g/t]acgggtatggagtgattgttaccgggtcgggttttggg[t/c]tccgggtttggttcttccga[a/g]tggatgaagaaactgggtatggtagtaagtcccttatcaacaagacctttaactccgattttcgactcgtcgaat

.5719

tg[t/c]ttcagttgttcgagacgatcggagttcggaactgggtttgggttcagaggaccggaattcgccatttt

.5635

tgtaacgagcttttt[t/g]agctacacagaaatggcggatttgagttgtgaagaagaaaatgaatacaacacgtaattaatagttagaaaattcatggaagttaaaaaagatgtctgtgtcgacaatgg

TM20_D4H-forward: 5’aacacaccgggatctgtatgt 3’

TM20_D4H-reverse: 5’ccattgtcgacacagacatctt 3’

Ta: 55°C

*PC*, plastocyanin, PGSC0003DMG400041620

>Chr04:71247823..71248324

Cagtaactttgccaaccattccagctccctggtgaggtgcacagtagaaagtgtaagttcctttctcactcaaag

7124.7917

tgacactgtatgtctctcc[t/a]gctgcattcagaagatcctcttcagccatggaaatcttacttgcatctaca

.8000.8001 .8031

ccagctgggatttcatcctcatcgaatacga[c/t][g/a]ttgtgtgggaaccctgcattgttcttgaa[t/c]gtaattttctcaccagcgctaacgctgaagtttccaggaataaaagcaagactcccatcatcaccaccaagcaac

.8109 .8115 .8139 .8142

ac[a/t]tcaag[t/a]gccatggcattgctagcaagcat[c/t]gc[g/a]ctaacagtggtggcagcaacaac

.8187 .8194 .8204 .8207.8208 .8210

agcaccgacatctttcaaaga[c/a/t]gccttc[a/c]cggtcaatc[t/g]gg[c/t][a/c]g[c/g]

.8211 .8217 .8224 .8238.8239

[t/c]ggggc[a/g]gctgcc[a/c]ccttggcggatga[g/a][c/g]cggtgctaactctggaagatgaggaa

.8268

gc[a/c]ccagccttgaggccagtgaaagatggaatagcaacagcagcagaggtaacagtggc

PC-forward: 5’cagtaactttgccaaccattccagct 3’

PC-reverse: 5’gccactgttacctctgctgctg 3’

Ta: 61°C

*DHN*, 25 kD dehydrin, PGSC0003DMG400009968

>chr04:71449922..71448545

Cgtaacgtccgtgtttgtaattgctataaaaagcgcaccaagtctcatatttctaccacatccaattcaaat

7144.9850.9848 .9817

[a/c]t[a/c]atttcaatattcaaactttcactttacctt[a/t]aagttgttgataatttctttgattgaaa

.9788(indelA).9784(indelB).9781.9780

[a/t]aaa[g/a]aa[g/a][a/g]agaaatatggctgatcagtacgaacagaacaagccatcagttgaagagac

.9706 .9695 .9680.9679 .9676

tgttggtgccaacgtggaggcta[g/c]tgatcgtggt[g/t]tgtttgatttcatt[g/a][t/g]ga[g/a]

.9675

[t/a]aaaagagaggaaaaaccaagtcatgctcatgaagaagaggcaatttcatctgagttttgtgagaaagttaaagtaagtgaagaagaagaacacaaggaggaaaagaaagaagagaagaaacttcatcgatcaagtagcagctctagtagctcggtaagtctctctttcatatttttacgtcacatattttttaaagtttacgtcaaatcaataaatataacacacattttttctcatgatcatatttatttcacatagattatattgtccgctgactattttcaatttaagtaattaagtcgatagcgttggatccttgatcatgttataatattccgatgattttattcagctataaataattagaaatttgtatacttgagaaatttgtaaatgtataaacaaaagtcaatagttgttacttatctttttttttttttttttatagcaacaccatattaggcttaaaacaaaaaatcacgatatgctattgtaggttacttttaacttgataatatagagattaatacttgacactatttgttttcacaagtaaatttactgtttactttttctagtaggtatgaataaattatacgtgatcattgatatattgtacatagattatatattcatacggactatttttagtttaatcagttgagtgaacgacatattaatgttgatcgatttgcagagtgacgaggaggaagaaattggagaggatggacagataaccaagaagaagaaaaagaagggattgaaggaaaagattaaggaaaaaatatctggtgatcacaaggaagaagtgaaaacagaggatacctcagttccagttgagaaatacgaggaaacagaggagaaaaaaggatttctagaaaaaattaaggagaaa

.8744 .8735

ttgccaggtggaggacataagaagacggaggaagt[g/a]gcggcgcc[a/g]ccaccaccaccaccggctgcgg

.8669 .8666

tggagcatgaggcggagggaaaggagaaaaagggatttttgga[c/t]aa[a/g]attaaggagaaattaccagg

.8583

ataccactcaaagactgaagaggaaaaggagaaggaaaaagactaaattaaatcaatgttta[t/a]ttgtt

.8577

[g/a]atgttttttacttttgggatgttatgattgtg

DHN-forward: 5’cacaatcataacatcccaaaagt 3’

DHN-reverse: 5’cgtaacgtccgtgtttgtaatt 3’

Ta:57°C

*HMGCR*, 3-hydroxy-3-methylglutaryl coenzyme A reductase, PGSC0003DMG400009924

>chr04:71954921..71953970

Gttgaaaaccacggctaaggtatcaaaattagcaggttcctccaagaagaagagtaattccgacgccctgctggccgtcgggaacttcacaacgggggccctggtcatgccgtccctgaaaagaacgctagtagctccgccagaggcatagatagctttacaacctctgttagtactggcaactaaacacccttctgtagttgccatggggacggaatattcaca

71954.648 .636(c2_10566) tccgttaagcaacaaaggtccagcaatcccgactggaatttgaacgta[c/g]cctatgggcat[c/t]tcacag

.581 .567 cactggcctaaaatggactcgtaatcaaaaccatcaagaggcaaacca[g/a]ctagagacctacc[c/g]gtta

.546 .528 .510 gcctctgaaccgcttc[g/a]cgccgaatggaagcagc[a/t]cggtgacaatccccgag[c/g]cgagattcga

.455 .438

gggaataagaaggaatatcaccagaaacaacagatttgacaagg[c/t]cttcatcgtcttgaga[g/a]ggaag

.430

ag[g/t]tgcgggaggatccatgaatctagatacagaagtggaaatggatgaattgagggcggcggaacaagtagcagggcggcgatcttggttaagaaggaagcgagtttcgtcttcgtcttcaatatcccaggcgtcgtgagatgcacgtgcaataaatgactgtacgaaatcaatgccaaaaaaaccgaggaggtaaatgaaagaggcaatgagagagagaatggcagcgagttcggagagagtgacgacgtggagaggggtggagctgcggatcttatcacgccagcgatggagaaggtaataagcaacggagaagaagagagtgaagaaaatggcgttggttaggtagagagggagaggaagagcatcggaagctttaggggcaggggaaggagatcgacgaatggcggcggaagaagatttttgaggttgttttagaagatggtgattccggtgagagg

HMGCR-forward: 5’cctctcaccggaatcaccat 3’

HMGCR-reverse: 5’gttgaaaaccacggctaaggtat 3’

Ta:57°C

*GT8*, glycosyltransferase, CAZy family GT8, PGSC0003DMG400000827

>Chr05:1981114..1981669

Aaggcaaataattgaagtaggtcacaaaagtgcatataaatcatccaaacaacaaaaataaaaatctatgaaa

1981.187

[g/a]caacacaaagaatcttcccataatccaactacttccttccaaaaatctattatcaaaatcccatccacca

.265 .266 .267 .279 .283 .290 .296

atcacca[t/a][t/c][c/t]tccacgtcacc[c/t]cca[c/t]gacccc[a/c]cactt[c/t]tggtaaat

.361 .363

ccaccatccacctcaatccaacggttaaaattctctttaaccccaccaataacata[g/a]a[t/g]gagctttttctcctcaaaatcccaaaactacccttcctcctacaccaccctatcctacacacttatgtcctctttcgtctttt

.465

tacaaactcagtttcact[g/a]tcaccagctcactcttaacaattattcacactaccatcaaacgtcgtaatca

.524 .558 .574

aagatc[c/t]ccagttcgattcatcatgtctaacaaagaagtg[c/t]ttaatcaaatatctc[g/a]agttcg

.585 .592 .597 .627

actc[c/t]ccttag[c/t]tatc[c/t]gaaaataacgattgatgtcggaacaaatc[g/a]tagggtgcccataaactatccaatggacatggcttctttgag

MF8_GT8-forward: 5’aaggcaaataattgaagtaggt 3’

MF8_GT8-reverse: 5’ctcaaagaagccatgtccatt 3’

Ta: 55°C

*TPARL*, transmembrane protein, PGSC0003DMG400000829

>Chr05:2134589..2135305

Aagaaatgcacagatactgaagaacgaaaaaccgtagtccataaaaatggtgaaatgcgctcaatgagaacaagc

213.4720 ttatcataagcaaatagagtctacatatttgcctttcagcgaaaaggttaaaaata[g/t]tggctgcctgcctt

.4736 .4744 .4754

g[g/a]accagac[g/a]cggagcctt[a/g]atctaaattaacggaacaaatatctaattggatagtgtttc

.4796 .4819 .4821 .4840 .4843 .4847

[t/c]ccgatttgcttgcttttatgta[g/t]t[c/t]gttgtaccctttcatacc[g/a]ag[g/a]act[t/c

.4849 .4852 .4856 .4862

]c[a/g]gc[a/c]aac[g/c]aaatg[t/g]gcatctagactatcattcacttcctgcatgaaagtagtgacccaatgtcaatccactcatcatcaaacacaggcaatttttttcgaaacgttttagctactaattgttctatgtgagcatgcagtaattcttctgccctgtttctttttaacgatggcagagataagccttgtatcttatccagcaaatgtgtaacatagcatttactttggtattctaacgtcaaatataaaactgtttcatgcatagaagaagggagaaccagtca

.5140 .5156.5157

ggactaacc[g/a]gagaacagggatatt[c/t][a/g]taagataaacgacatggttactcgactgttgaaagg

.5196 .5214

ta[a/g]gactgaattccgaaaac[g/a]tgaatagagatccaccagcaagtcccacctacatcaagaaataactctgctctcatgaaaatggacaaaaggaacataggggtaagcaag

MF7_TPARL-forward: 5’aagaaatgcacagatactgaag 3’

MF7_TPARL-reverse: 5’cttgcttacccctatgttcc 3’

Ta: 55°C

*ATPase*, AAA-type ATPase, PGSC0003DMG400031271

>Chr05:5067838..5068521

gatcgtaatgctaaaatttgagtgttccatgataaaaaaacttcttattaaccttcaccaactacatgaaaacc

506.7934 .7939 indel1,2 .7971 Acttgcagaattatgtgtattc[a/g]agca[t/c]cactaagacataaaataaacttattacaagt[t/a]cag

.7981 .7990 .8000 .8019

ctaata[a/g]acccaata[a/t]tgtagaaaa[c/t]ataaagctaaaacacccc[c/t]aagtacactttcat

.8034 .8042 .8060

[a/c]tatgtgt[g/a]ttcatcaatctttgcta[g/a]catcaccattttccttaacaccattatctttttcgt

.8102.8103 .8116

ttaca[c/t][t/c]gtcagcattctt[a/g]gcttcttcagtagctgttacttctttcttttcctcgcgatctt

.8176 .8218

tctcttccttctcctt[c/t]tcagccgcctttgctttctcttcttcctcagccttcagttt[c/t]gcttcctc

.8260 .8269

ttttgcagtttcaagagctttaatcaatctctc[c/t]aaacaagt[g/t]tctgcattttcctttgaagactt

.8293 .8323 .8326

[a/g]ggcatcaaattctcagcaatatcagcagg[a/t]gt[a/c]atattagtttcctccaataaacgacgaatctcaggaaagtgaacatgagattcaacgacgtcaagatagttatgtgcaagaactttgaatgactcaaagcaaca

.8431 .8491

[g/a]taggataggacaatatgtttatccatcctccccctcctaattagagcaggatcaagctt[t/c]tccac

.8497

[a/g]tagttggtagtgaagacgataagc

TM35_ATPase-forward: 5’gatcgtaatgctaaaatttgagt 3’

TM35_ATPase-reverse: 5’gcttatcgtcttcactaccaa 3’

Ta:57°C

*Coi1*, Coronatine insensitive 1 (AY423550), not annotated

>chr05:47900068..47900691 (amplicon A) Gctggatgctcggagactacagtttctgcattatttccatcgttggcaataactcgtcttgcaggaatcaattca

47900.154 .166

atattccagaa[t/a]gggcgagccat[c/t]gctaagagatcacgaccagctgaagatgccctgtatccttgta

.243 .262

cccacaggtaccttaacgattttagctgcaagg[t/c]agccaaagctaatgcacg[t/c]tcactgaaacagca

.300 .319 .327 .335

gcctctcacttctagcttttgca[g/a]gctcggacatcctttaga[g/a]aactcca[g/c]aaggcca[t/c]

.340 .352 .390 .394

gatc[a/g]gattccccaac[a/g]tatcccagaagcatccatctcacgtttgggctgtatt[t/g]ccc[g/a]

.442

acataactgagacctacatcagtaaggcccccaggccggacatagag[g/a]gcaaatcttctaagattatggca

.470 .478 .517 .518 .519

acct[c/a]ttagtaa[t/a]gcacggacaccattatcaagtggcagatctgttattct[a/t][a/t][t/c]t

.555 .579

tctcggtcaagcaaaaccagccgaaaatcactca[a/g]atttttcagatatgtaccaataa[c/t]ttccaaag

.636 .640 .646

cttcattagtaatatctgacacataaacagccatgtattctagttcaa[g/c]aca[t/g]ccctt[t/g]gccaaatcaatcaatcctctgtgtgtaaccgcaccttcttcatcc

Coi1_A-forward: 5’gctggatgctcggagactac 3’

Coi1_A-reverse: 5’ggatgaagaaggtgcggtta 3’

Ta: 59°C

>chr05:47907866..47907399 (amplicon B)

Ggaggaacggaactcaacgagattgagtagctcaacaaacgatacagtatgggagtgtgtgattccgtatataca

4790.7767

ggaatcgcgtgatagagacgcggt[g/a]tcgttggtatgcaagaggtggtggcagatcgatgcgattactagaa

.7713 .7689 .7687.7686

agcatat[a/t]actatggcgttgtgttatacagc[g/c]a[a/g][g/a]ccggagcagctatctagaaggtttccacatcttgaatcggttaaactgaaagggaaaccaagagctgctatgtttaatttgataccggaagattgggga

.7557 .7543

ggatatgttacaccttgggttgtggagat[t/c]actaagtcgttta[a/g]taaattgaaagcgcttcattttc

.7509 .75048

gtagaatgat[t/c]gtta[g/a]agattcggatctcgaattgcttgcgaatcgtcgtgggaaagttcttcaagttttgaagctggataagtgttctggattttctactgatggtcttctgcatatttc

Coi1_B-forward: 5’ggaggaacggaactcaacgagatt 3’

Coi1_B-reverse: 5’gaaatatgcagaagaccatcagt 3’

Ta: 57°C

>chr05: 47902013..47901508 (amplicon C)

Ctgcaggaacttaagaactttgttaatggaagagagttatataattgagaaagatggagaatgggcacatgaactagcatcgaacaacactgttcttgagaatttgaacttttacatgacggatcttctgcaagttagggctgaagatct

4790.1833

tgaattgatagcaagaaattgtaaatctct[t/c]gtctctatgaaaattagcgagtgtgaaattacgaatcttc

.1773 .1725

ttggcttctttagagctgc[g/a]gctgcattggaggagtttggtggtggcgcatttaatgaccaaccaga[a/g

.1696 .1690

]cttgttgaaaatggctataatgagcaat[c/t]gggaa[a/g]atatgccgcactagttttccctccaagattatgccaattgggcttgacatacttagggaaaaatgagatgtccattctctttcctattgcgtctcgtctgaggaaattggatcttctttatgcacttcttgacacagcagcccactgtttcttactgcaaaggtgtcccaacttggaaattc

Coi1_C-forward: 5’ctgcaggaacttaagaactttgt 3’

Coi1_C-reverse: 5’gaatttccaagttgggacacct 3‘

Ta: 59°C

*StGP28*, Stem 28 kDa glycoprotein, PGSC0003DMG402016495

>Chr06:49101573..49102152

49101.609

Cagagacaatttcaaacaagtgaggattgctaatct[c/a]aagaaagctggttataccaactgggcagcgctcatactaaagtaaccaaatttcttattttcactttatttgttcttattaccacatagtactatatgttattaattta

.740 .745 .748 .752 .758 .763

tgtgattacaggggagaaaat[g/t]attc[g/a]gg[a/g]tca[g/a]cagtg[g/c/a]aatt[t/c]aaat

.769 .772 .794 .804 .811

c[g/a]ag[a/c]aagagaacagcattggtgaag[g/a]ctggatata[g/t]aattgt[t/g]ggtaatattgg

.834 .886

agatcaatgga[g/a]tgatcttattggaaaaaacgttggagcTcgtacttttaaactccctgaccc[a/t]atg

.896 .898 .909 .916 .927 .937

tactac[a/g]t[a/t]ggttgactct[a/c]accaaa[t/c]tttaatcaac[a/t]aaggtgggg[t/a]a

.939 .953 .957 .989

[c/g]cttttttattttg[a/g]cat[t/g]tgagatattataagctcactgttcCtaatcc[t/a]catcactt

.1059.1060

tgctataaataacgacttccttcaatattttaattatgttccttttttttattaattttaa[a/g][t/c]taaaagacgcacatctctacttttttcctatttcagtttagactctagaggatcacatcaccacgcattaattaaattgagcaacgaagtac

MF12_StGP28-forward: 5’cagagacaatttcaaacaagtg 3’

MF12_StGP28-reverse: 5’gtacttcgttgctcaatttaat 3’

Ta: 55°C

*RPS27*, ribosomal protein S27, PGSC0003DMG401028933

>Chr06:50409859..50410345

# Ctagaattgtcatttctctaccatggtgaggaagtattgggttccacaactccactatccatacatatagtcttc

504.09952 .09987 .09997

# ttgagcccaaaagaaacc[a/g]tcatctcaaactaagacacacaaaaaagatgtgg[c/a]gaaagaaga[g/a

# .10004

# ]aaagaa[g/a]aatatgacaaaatcaaggatgacaatgaaaacaagtacccctttaatgcatctaatctaagtg

# .10076 .10097.10098 .10118

# gatgtcaa[c/g]gactcacaggcaagtcattg[a/t][a/g]gcaaagggaaggtt[a/g]tgccattcttcca

# .10158 .10185 gatcttttttttctttgttgtgttttgggtt[t/a]atgtttttcatatacattcaaaagtt[g/c]ggctggac

# .10208 .10217 .10252

# tttgaagtttcact[g/t]tatatgta[g/a]tgtatgcttgttttgggttgaagttctcttaata[g/c]agtgattcttgttaccttatcgtgtcgatgtatgctgtcatcctgttctccccaattccactactttcttggcacactttaccaataaacaag

# TM41_RPS27-forward: 5’ctagaattgtcatttctctacc 3’

# TM41_RPS27-reverse: 5’cttgtttattggtaaagtgtgc 3’

# Ta:57°C

*ICE2*, inducer of CBF expression 2 protein (ICE), transcription factor, PGSC0003DMG401028788

>Chr06:50575325..50574351

5057.5297 .5270

Aagaaggtcaagactttcatcctgatca[g/a]atcaaagcagtactgctggattccgc[c/t]ggttgccatgg

.5216 .5191 gatgatataagcttctatgattgacaaagggaaacaatgtag[t/c]cacaactttcaactttagactcta[c/g]ttgttgttcttagccaatttcctatgctccttttgtatttctatatttcatccagtaggctgttgttttgtgtt

.5107 .5061

acaactcac[a/t]atttgctattccatgaacctttgctttcatttactagttacttag[t/c]gaaccgattaactagcagttttctcaattgttctgcaattttagtgtataaaaagcatatgcaaggaaaatttgaatgtgtcaatgaaatacactcattaattctgatattatcttgatttattttccaccttgggatgtcgtgaaatagttgtactgcattcaccttcaactatacatccaggttcgagttctggaaatggaaagttttccgtccaactactggatggttaaagc

.4807 .4789 .4783

aaaaaagggaaaaaatt[t/g]aagggtaatatctaggc[c/t]tgttt[g/t]tctacatatacagtgctcggccatctttattgggggatttaagatggtctaagtgcttctgcattttttttcaccaggccaacaaaccatagaa

.4688 .4686

[t/g]g[a/g]ctgcatgcataaacttctgatctgctaagacatggaatcattctcttcaacatcattattatta

.4570

ttataagtttattgttctgtcaaagaggttttcattccattacaaaaagga[t/c]aacacatcttctaacaaga

.4516 .4504 .4487 gactcaatgctgcatcctcaaggtgatatcaaca[t/a]aggcaaatagg[g/t]cccaagtaggtttcaa[a/g

.4484 .4468 .4461.4460.4459 .4451 .4449

]ct[g/a]ctcatacccttttga[c/t]gtcttg[t/c][c/t][a/g]attcgga[t/c]t[g/t]gactgctatcatagggacatatgtgacttccaactgcaaacaatgcaacaaaatcagagtgtgatgaagttggcaagaataaagccattattacagtg

MF10_ICE2-forward: 5’aagaaggtcaagactttcatc 3’

MF10_ICE2-reverse: 5’cactgtaataatggctttattct 3’

Ta: 57°C

*EXO*, endo-alpha-1,4-glucanase, beta-D-glucan exo-hydrolase, PGSC0003DMG402005942

>Chr06:54347092..54346464

Cccataactaacttctggaaaagatttaatagttccttttgtaccttatcttctgttgacaagtattctgaaacc

54346.999 .966

agctcttaacatgataaa[a/g]ctttatccaaaagctgaatgtcatacacacta[t/c]ggtatcaatgctgt

.951 .939 .934 .931 .924

[c/g]ttttaattcat[c/t]ttta[c/g]tg[c/g]ctgagt[a/t]gtgagtccaaatccaaagggaaagagg

.886 .858 .855

ggattgtagt[t/g]gcgatcgccgacattcataggaagctg[g/a]tc[c/a]actgacttgaaccaagtccta

.815 .771

gcaagtttaccagtgaaa[c/a]catagtcaccaaataaaacatctgcaacaccttggccttcagt[t/c]cc

.768 .751 .750 .738 .735

[t/c]ggaagccaagcagcaa[c/t][a/g]actgcatctat[c/t]tt[t/a]tcaacataaggctctagcaca

.697 .696 .691 .678

actggacgaccagaga[c/t][a/g]atga[c/t]tacaacacattt[c/t]acagccccacagacattgttaat

.611

agtgctaggaccaggttctgttattgtaagatttgagctgtcg[c/a]ccatcatctctgcatatgggacttcac

.577 .546 .528

ctacta[c/a]aacgatggcatagtcaaattcgtttgactt[g/c]acaaagtttgcatcagg[a/t]ttctgct

.504 .501 .496 .495

ggtacactacttgtgt[a/g]ga[a/c]ggat[c/t][c/t]acagttttcttgatagcacttaaaatagtgg

MF11_EXO-forward: 5’cccataactaacttctggaaaaga 3’

MF11_EXO-reverse: 5’ ccactattttaagtgctatcaag 3’

Ta: 57°C

*StTL15A*, thylakoid lumenal 15 kDa protein A, chloroplastic, PGSC0003DMG400034939

>Chr06:56859698..56860110

Cagatagatcagcaccagttaaatcggcatcaaagaagctagctccgagtaacttcgcacctttaaaattagctt

568.59810 .59831

Gtctgagtattgaagttttaaaatcttgtttgattaa[c/a]gttttcccactaaaatcctt[c/t]cctgttag

.59849 .59873 .59879

atcttggcc[c/t]cttgttactccagcaccgtaagg[g/a]cctcc[g/a]cctttaaaggctaaagcaggat

.59902

[c/t]tgaagcaaagaagagtgaagcagagagaagggccagaaaacttgctcttgagattgattcgcttaatga

.59972 .59991 .59997 .60015

[t/g]atgaaaccatttctcgaa[g/a]attca[g/t]ctgattttagagaaacc[t/a]]gtgatgtagcttga

.60031 .60078

t[g/c]agtttgtggggaaagatggagattgtgaagttttggatttgagggg[a/g]gttttgaacaacagaagcagaagctgagaatc

TM18_StTL15A-forward: 5’cagatagatcagcaccagtta 3’

TM18_StTL15A-reverse: 5’ gattctcagcttctgcttctg 3’

Ta:55°C

# *OPR3*, 12-oxophytodienoate reductase 3 (JN241968), PGSC0003DMG400030890

>chr07:4058602..4058988

Tccaactccattctggttccaagcttgcctatcttcatgtaacacagccacgatacgtagcatatgggcaaaccg

4058.701 .704 .707 .713 .720

aagcaggcaggcttggcagtgaag[a/t]gg[a/t]gg[a/t]ggcgc[a/g]tttaat[g/a]aggactttgag

.738 .741

gaacgc[g/a]ta[t/c]caggggacattcatttgcagtggtggatacactagggagctaggaattgaggctgtg

.834 .855

gcacaaggtgatgctgatctcgtgtcatatggacg[t/c]cttttcatctctaatcctga[t/c]ttggttatga

.928

gaatcaagctaaatgcacctctaaataagtataacaggaagacattctatactcaagatcca[g/a]ttgtggga

.958

tacacagattaccctttcctt[c/a]aaggaaatggaagtaatggaccgttatc

OPR3-forward: 5’Tccaactccattctggttccaag 3’

OPR3-reverse: 5’gataacggtccattacttccat 3’

Ta: 63°C – 60°C (touch down)

*CAB13*, chlorophyll a-b binding protein 13, chloroplastic, PGSC0003DMG400019248

>Chr07:53735139..53734538

53735069

Cttagtgaaaattgtgaggtccaattaattaagagagaaattaagaaagagaagaaatggcagcaacagg[c/t]

agctcagccacagttgttagagcaactccatttttgggccagaccaaatatgctaacccccttagggatat

53734.991 .985 .981 .980 .977 .974 .973 .970

agttcc[t/a]atggg[c/a]tct[g/t][c/a]ca[g/a]at[t/a][c/t]ac[c/t]atggtaatttttatt

.903

tttattttgtgattttataattgagttcgaaaattaatatttattgaaatt[c/a]gaataaatttatgattcgt

.880(6) .841 .836

gta[c/a]aaagatactgaattaagttgaatctgtttttaatgttt[c/t]gtta[g/a]agtaatgatttgtgg

.754

tatggacctgaccgtgtcaagtacttgggaccattttctgctcaaactccttcatacttgactgga[g/a]aatt

.749 .737 .710

[c/t]cctggtgatta[c/t]ggatgggatactgctggtttatctgc[t/g]gatcccgaggcctttgccaa

.689 .685 .642

[g/a]aac[c/a]gagctcttgaggtactatattttttcttttactagtagtaac[a/t]gatatttatgtgactaaaaatcattttcgtaaaattgtttctaaatattaaaggtatcatttttattaggatagaacaaaaaagtgaagcagactcatataatt

MF14_CAB13-forward: 5’cttagtgaaaattgtgaggtc 3’

MF14_CAB13-reverse: 5’ aattatatgagtctgcttcactt 3’

Ta: 57°C

*THI*, chloroplast thiazole biosynthetic protein, PGSC0003DMG400019257

>Chr07:54329580..54329033

54329.537

Gtgctccatcaatttcagcaacttccattcctgtaacaatcat[a/t]ccaggtacaacttctctggtaagtcta

.504 .453

acaat[c/t]gcgtcctcagcagcgttcatgtccaaagctttcattccaggaacactgtt[a/g]atcatgccaa

.435 .426 .414 .396 .393

tgctcct[g/a]agcctctt[a/c]acaccggtggc[g/a]cccatggggccgtcgtg[g/a]cc[g/a]cagga

.357 .345

actgaccacaaccttagcctccataacatt[a/g]gggtccatgca[t/g]gattgtgtgtcatggttctgggaa

.303

accaaagaccagttagt[g/a]acaacaccaccgactcttccgttcttcacgataaggtcctctgttgcaacagcattgaagagcttcacatttggcctggccaaaagcttgctcatgatggttgaggtgaacaaggcagcgtgtttgatgaccacgtagtggtcttgctcgtcgtagtcaatgcctagctcgttcaggaaaagatgtgctggcttacgcacaaccatagcagagaagagttgtccgcctagccaagcacctccaccagggctcacagattgctcaagaatg

TM46_THI-forward: 5’gtgctccatcaatttcagcaa 3’

TM46_THI-reverse: 5’ cattcttgagcaatctgtgag 3’

Ta: 57°C

PSBR, photosystem II 10 kDa polypeptide, chloroplastic, PGSC0003DMG400022241

>Chr07:56365818..56364862

Cgactgaacttagaagattaagattataagaacctaaaacagcaacattaggtgtaatattatccacagtaaaa

5636.5744 .5714.5713

[t/g]cacagcaattacatattaatcaagatgcc[t/a][g/a]gaaatattttgaagtatttttgaaaagatta

.5677 .5675.5674 .5671

catg[g/a]a[a/g][t/c]ac[a/g]acttaaaatagtacaatacaaggataacatctactgtgccaaagcact

.5621 .5586

t[g/c]tgttgtagacaaggagagcacctcctgcaagaat[g/a]ccaaccaaggtcacggcccatatggctaa

.5556 .5553 .5550 .5518 .5514

[g/t]cc[a/c]gt[g/a]gtacctgcaattatattacatgtgaatcagg[a/t]tct[a/t]gagtcagtagat

.5492 .5470

Tcaaaatga[g/c]tacgacttaattataaacgtt[c/g]ttgtgtatatacatagttcgagttaaaaaaaggaa

.5404 .5390 .5383

Gttcaagtaaattgtcatacctccaacata[g/c]acatcaccacttg[g/c]agacca[c/a]tcatctgtgttgtagatgggactgcattttcaaaatcacacaaagtattagattatttgtttcattttctattagtgaagaattacattaaacaaattaagaagtttttaactaacctgtatccatcaacattagctccatatttgtcaacatattggtacacaccctttccctaaacatcaccatgttttgctcatatattagaaaagtaataaattaaaatatacacacttataaactacagaaaagcaattgctttaatttacattcttttattttgaaaaaaaatggaaatattattttactactaa

.5000

ttaatgatattattatatatatcaaaggtagaagtaaacacaaaccttgggcttcctgcctgaggcatcaa[g/c

.4999

][c/g]ccatctctcaaggccatgcttccattaattcctagtaacaaagcccacttcataatcagcagatagtattaattttgcattacaatattttttctctggtttttttttttttcaactacacatatatagacatagtt

TM25_PSBR-forward: 5’ cgactgaacttagaagattaag 3’

TM25_PSBR-reverse: 5’ aactatgtctatatatgtgtagtt 3’

Ta: 57°C

*psaD*, photosystem I reaction center subunit, PGSC0003DMG400005805

>chr08:5483147..5482300

tctctgagtttgaagaattgttagcacacagatatcatttagtgtaagatggacaaattaaagtaatcaatacga

548.3054 .3030 .3022 .3019

aagattaattaattaaaa[a/g]gataatggatagtaaattacttc[t/c]cactgtc[a/g]ca[a/g]tagat

.2979 .2975.2974

acatacattttgggaaatgagtaaatacataagt[c/a]ttc[a/t][a/c]gctaagcttcaccatttccagaa

.2932 .2907 .2905

cacatttgaaccacactt[g/a]tatttgtccttgccttcgcactcc[a/c]a[c/t]gccaaatcgtcacttat

.2859 .2829 .2825

aaatggcactttctgaatttcatttgta[a/c]aaacacaaatattagcatatcaattcatt[t/a]gtt[c/t]

.2820 .2817 .2772

aaca[a/c]aa[a/c]aaaaattaatgaaaaaggttgagattaccttttgcttggcaagg[t/g]cttggcatcc

.2759 .2739

tg[t/g]gaagttctcagggaacttg[c/g]aagttccaaactgaacagtatatgcccttgcgaaattcgcaccacttgtagccaacctcttcttgtcattcaattcctgtagattgactgaatcacgttaaatgtttgtgtttttttggatatttctcgataataaataagcatacatatataccttgttggctaggcttttctcaagatattcgtcaataacaccagcattagcagaagaagcagtggtgaaaagggcagcagcaaggccaaggagagcaactcttcttccagctgct

.2445

atttggtccttgtccatgttggag[t/c]tggagctggattgttgttgagccttaatgattaatggcaatttatg

.2392

aagtga[t/g]ggggaagccatagtaacagtatattttgatgtgagttcagatgagcccatgcctgatacagcatagctgcaagccaatacacttgagttcat

TM47_psaD-forward: 5’tctctgagtttgaagaattgtta 3’

TM47_psaD-reverse: 5’atgaactcaagtgtattggctt 3’

Ta: 57°C

| *Plox1*, potato 9-lipoxygenase (AF019613 ), PGSC0003DMG400020999 |
| --- |
|  |

>chr08:6048430..6047473

Cccctgagcatgtaaacaaaggcaaatgttcaaactcaaaagttttagcaatgtcttgattgtttatgcagccat

604.8344 .8321

atctcccaagt[a/g]aaacaccagagcttttgcgaaa[a/g]tacagagaaaatgaattgctaacattaagagg

.8264 .8231

agatggaactggaaagcgcgaggc[g/a]tgggataggatttatgactatgatatctacaa[t/c]gacttggg

.8222 .8218 .8216 .8213.8212 .8206

[c/t]aat[c/t]c[g/t]ga[t/c][c/g]aaggt[c/a]aagaaaatgttagaactaccttaggaggttctgc

.8168 .8140

tga[a/g]taccCgtatcctcggagaggaagaact[g/a]gtagaccaccaacacgaacaggttagttaggc

.8107 .8096 .8089 .8083 .8062

[g/a]ttcacaaata[a/t]cagtcc[a/c]ttcta[c/t]gtacttactcaacgcgtata[g/t]cttcattga

.8050

aa[g/c]caactttttgatcagttacattgttgctttgtatagatcctaaaagtgaaagcaggattcctctt

.7984

[a/c]ttctgagcttagacatctatgtaccgagagacgagcgttttggtcacttgaagatgtcagacttcctaacatatgctttgaaatccattgttcaattcatcctccctgaattacatgccctgtttgatggtacccctaacgagttcgatagttttgaggatgtacttagactatatgaaggagggatcaaacttcctcaaggacctttatttaaggctct

.7736 .7734

cactgctgctatacctctggagatgat[a/c]a[a/g]agaactccttcgaacagacggtgaaggaatattgagatttccaactcctctagtgattaaaggtacaatttgttacatcataagaagtaatctagctaatgatcactcactt

.7618 .7572

tta[t/c]gtgtttgtatgatttaagtattgattgtgcagatagtaaaaccgc[g/a]tggaggactgatgaagaattcgcaagagaaatgctagctggagttaatcctatcataattagtagacttcaagtaagttggtttcttcgcgattaggaa

Plox1-forward: 5’cccctgagcatgtaaacaaa 3’

Plox1-reverse: 5’ttcctaatcgcgaagaaacc 3’

Ta: 57.4°C

Potlx3, potato 9-lipoxygenase (U60202), PGSC0003DMG400010859

>chr08: 17830114..17831043 (amplicon A)

cacacgcagccattgagccttttgtgattgctacaaacaggcaactaagtgtgcttcacccaattcataagcttt

17830.205

tacatcctcattttcg[t/a]gacacgatgaacataaatgctttggcaagacagatcttaatcaatgctggtggagttcttgagatgacagtttttcctgccaaatatgcgatggaaatgtcagctgtagtttacaaaagttgggtttttcctgaacaagcacttccagctgatcttataaagaggtatacattataaatttagaacgaactttactagaaaaat

.417

atggttg[t/c]tgatgaagtttaatttccgggtattttgattttgctaatatgaactataatatcatttga

.478

[t/a]tttaggggagtggccgtagaggactctagttccccacatggtgttcgcttgctaattcaagactatccatatgctgttgatggcttagaaatatggtcagcaatcaaaagttgggtaaccgaatattgcaacttctattataaat

.673

cagatgaattagttttgaaagataatgaactccaagcgtggtggaagga[a/g]cttcgagaagaaggacatggt

.711 .747

gacaagaaagatgaac[c/t]ttggtggcctaaaatgcaaactcgccaagagctaa[a/t]agattcttgcactattattatttggatagcatcagcacttcatgcagcagtcaattttgggcaatacccttatgcaggttacctcccaaatcgcccaacgttaagtcgaagattcatgcctgagccaggaactcctgagtatgaagaactcaagacaaatcctgacaaggcatacttgaaaacaatcactcctcaactgcagacattactaggaatttctctcatagagatattatcaaggcatgcatcagatgagatttaccttggacaacgggactcatccgaatggacaaagg

Potlx3_A-forward: 5’cacacgcagccattgagcctttt 3’

Potlx3_A-reverse: 5’cctttgtccattcggatgagtcc 3’

Ta: 63°C

>chr08:17827331..17828296 (amplicon B)

Agtggaggctatctctgggagaagtgaggacaatggaaaaaaggtgaaaggaacagttgtgttgatgaagaagaa

1782.7410 .7470

tgta[c/t]tggactttaatgatgtcaatgcctctcttcttgatggagtacttgagttccttgggaaa[a/c]ga

.7495 .7517.7518

gtctctttgcagttgatcagcg[t/c]tgttcatgctgatcctggtaa[t/a][t/c/g]acttctttagcttcc

.7549.7550 .7589

cttgttttaaactat[a/t][t/c]atagataggtccatcaatatTaattcctaaaatgttgt[g/t]aaagtgg

.7616 .7618 .7657

agtactaaaatagaatcat[c/g]t[c/t]aaacattttaaaatatctcaaaataaaacgactattat[g/a]ta

.7706 .7714

aattgtgagtcaaattcgatcttgtcactacgccacaaaaatgatc[t/c]ttgcaat[t/g]atttaattgcgg

.7738

tttttctagag[g/a]caattgacattctttgaaaatgtctctaaaacctatagtgacattaatccaaatttagccatcaaaagatagggttgaccacaaaacgaagaacgaaactttacgactaactttttttctcgtagacgttcacttgtactgacaatttactttccttctttctgaaacgattaaactgattgtcaatagatgttgaatttatttataaaaggttaaaggtttgaagggtgttttatcctaaattgatagttgaatgagtttgctatctagttgattaaccttgtgagatattcatattaatggaactaactatagatggaaatttaatatttagtgaatctgatcgatttaacaggaaacagtttacaagggaaacgtagcaatccagcttacttggagaagtggctcactacaggaacctcattagtagcaggtgaatcagcctttgacgtcacgtttgattgggacgaagatattggcgtaccaggggcatttattatcaataatttccacttcaatgagttttatctcaagtcactcacccttgaagatgttcct

Potlx3_B-forward: 5’agtggaggctatctctgg 3’

Potlx3_B-reverse: 5’aggaacatcttcaagggt 3’

Ta: 59°C

*CYP71D11*, cytochrome P450 71D11, PGSC0003DMG400020809

>Chr08:46879159..46880058

46879.180 .183 .196

Ttcttctcactgtcacaccaa[a/g]at[a/t]cacctcactcat[g/a]tccaattcttcatgtttcatcccat

.244 .282 tgggtagcttccaatcaaaatg[g/a]tatagaaaaagagccaatggatgctctacatttgcta[g/t]accaaa

.316 .328 .332 .346 tgacatgccaggacacatccttcttcc[t/a]gcaccaaatgg[t/a]atg[t/a]actcaaaattatt[c/t]c

.351 .385

cat[t/a]gaaatcaattgaactatcaagaaatctctctgc[t/c]ttaaagcactctgcatcctcccaatatct

.415 .432 .437 .457 .460 .462

[t/c]gagtctcttccaattg[t/c]ccaa[a/g]cattcaccaacacttgagt[c/t]tt[g/t]t[g/c]t

.464 .469 .502 .504 .517

[g/t]gtat[t/c]tcataaccatcaatgttacatttttctctact[t/c]a[g/t]ccttgggagtaa[c/t]a

.524 .541 .543 .544

aaggg[g/c]caggtggatgcaacct[t/g]a[g/a][c/t]gtctctttgatgactgcttttaagtatttcaat

.629 .636

tgatcaaagtttgattcatccacataacctttttcaccaaaaactctcctg[a/g]cttctt[c/g]ttgtgctt

.655 .657 .688

Tctctaagac[a/t]c[t/c]tgggttcttgagcatttctgccattgtcca[a/t]tcaacagttgttgctga

.706 .707 .751 .752

[a/g][g/c]tatctgttcccgctgcaaacatgtcctgttacatgtgtacatc[g/a][t/c]atgaatatatgaacatgtcataaattgagaagttcatatagcttctacatataaaagtatgtcattctttttggaactgactaaaatgagaagagtatcggagtagagtgaactaaccaagatcactgctttaatgttttttcgagttatttcataatttcc

.945 .982

atcattttgtactcttaaaaggacatccac[a/c]aggtcctcatgcataattgtggtgttgttgtcaaca[t/a]caatatgatcatctataatctcatcaagtagcctatctacttcatgatgaagcttctccaattttgacctaagacc

TM27_CYP71D11-forward: 5’ttcttctcactgtcacaccaa 3’

TM27_CYP71D11-reverse: 5’ggtcttaggtcaaaattggag 3’

Ta: 57°C

*HSP70*, heat shock protein 70 kDa, PGSC0003DMG400008917

>chr09:1959581..1958865

gaactctatccatgatgttgtacttgttggtggctccaccaggattcccaaggttcagcagctattgcaagactttttcaatgggaaggagctctgcaagagcatcaaccctgatgaagctgttgcttacggtgctgctgtacaagctgc

195.9410 .9365 aattttgagtggtgaaggtaa[c/t]gagaaggtgcaagacctgttgttgttggatgttacccctctttc[c/t]

.9329 .9320

cttggactggaaactgctggaggtgtcatgactgt[a/g]ttgatccc[t/c]agaaacaccactatcccaacca

.9290 .9260 .9257 agaaaga[a/g]caggtcttctcaacctactcagacaatca[g/a]cc[a/t]ggtgtgttgatccaggtctatg

.9226 .9218 .9182

aaggtgag[a/c]gaactag[g/a]accagggacaacaacttgcttggcaaatttgagct[c/a]tctggtattcctcctgctcccaggggagttcctcagatcacagtgtgctttgacattgatgccaatggtatcttgaatgtttctg

.9083 .9038

ctgaggacaagac[t/c]actggacaaaagaacaagatcaccatcaccaacgacaagggcag[g/a]ctctccaa

.8990

ggaggagattgagaagatggttcaagaagcagagaagta[c/t]aagtctgaggatgaagaccacaagaagaagg

.8927

tagaggcaaagaatgctttggagaactatgc[a/g]tacaacatgaggaacaccatcaaggatgagaagattgcatccaagctgcctgaggcagacag

HSP70-forward: 5’gaactctatccatgatgttgtacttg 3’

HSP70-reverse: 5’ctgtctgcctcaggcagc 3’

Ta: 60°C

*Lox1St2*, 9-lipoxygenase (U24232), PGSC0003DMG400031809

|  |
| --- |

>chr09:53386326..53387163

Gtggaagtggttcaggcaagctcaaggagtgggacagagtgtatgattatgcattctacaatgatcttggatttc

5338.6411

cagataaggg[g/a]ccagactatgttcgacctgtgcttggtgggtcgaaggaatatccatatcctcgtagagg

.6471 .6503 .6505 .6510

[c/t]aggacaagccgtcgagcaactaaaacaggtt[a/c]g[a/t]tcac[a/t]tataaaattgaagtc

.6526 .6544 .6566 .6571

[g/a]tcatattacaaggttat[g/c]acttatgatttgcattgctgt[a/t]gaaa[c/t]ttcattagaatga

.6615

aatgaattcaagaccaatgaagtactatgg[a/g]taataagcagctgttattcctattggctttgcagatccta

.6667

tgtcagagagc[c/a]ggttgccacctttaggtttggacatttatgttccacgagatgagcgtttcactcctgtg

.6744 .6762 .6771 .6778

aagctttcagatttcct[a/t]gcatatgctgttaaatc[a/t]ttgggtca[g/a]gtcctc[a/g]ttcctga

.6791 .6827

gattg[t/c]tgctttatttgacaaaactatcaacgagtttgata[g/a]ctttgaggacgtactcaagctttat

.6870

gaaggtgggattaagct[g/a]cctgaccatcatttaaaaaaactcaggcaatgcatcccatgggagatgct

.6921 .6939 .6981

[c/t]aaagaactagtccgctc[c/t]gatggtgaaccatttctcaaatttccgatgcctgatgtaat[c/t]aaaggtaccaaagggctcgtttaatagatacttcctgagatgttagatcttgcatcataaattgataactaattata

.7073

agcatctttttaaa[a/c]ttactgcagttgataggtctgcctggaggacagatgaagagtttggacgggaaatgctagctggagtcaaccctgttatcattcgacgtc

Lox1St2-forward: 5’gtggaagtggttcaggcaag 3’

Lox1St2-reverse: 5’gacgtcgaatgataacagggtt 3’

Ta: 63°C

*Rpi-vnt1*, gene for resistance to *P. infestans* from S. *venturii*, PGSC0003DMG400020587

>chr09:59560383..59560773

59560.433 .440(c2_47952)

Gcctacatgatcatatcgagaagtgataattatcctactgccaatttttg[a/t]atcaca[t/c]tcaggacga

.461 .465 .473

aggacacggtt[c/t]aga[g/t]catccca[a/g]attttagtatcccaaatgtcatctaagagaataacatac

.527 .535 .539 .544 .552 .565

ctttttgtttccaa[a/g]agtgatc[g/t]tag[a/g]ttgt[c/t]ctccaag[t/c]gttctttccttt[c/

(c2_47950).569 .573 .592 .618indel

g/t]ctc[c/g]tcc[g/a]tcagtccaacttgcttgg[c/g]tatgtcaagtaagatttctcctgct|cttggc

.632 .662

tgttgtga[a/g/c]acgtagaccagtgcagaacactcaaattg[t/a]tgacggacatgcctataaagtttcttggcaagagttgtttttcctagaccgggcatgccaactattgaaacaactccattacacaaatcttgaacaagcaatttgtcttgg

Rpi-vnt1-forward: 5’gcctacatgatcatatcgagaagt 3’

Rpi-vnt1-reverse: 5’ccaagacaaattgcttgttcaagatt 3’

Ta: 65°C

*VAMP*, vesicle associated membrane protein SEC22, PGSC0003DMG400028151

>Chr10:56090969..56091509

Acttccagtggcaataaactcctctgcttcatcatcttctccaaattactaaacactcacatcacagctccgggc

609.1077 .1079 .1093.1094

aagttgatatttcacggcgttatttccgacgaa[c/a]a[g/t]tccgccgtcgccg[g/a][g/c]atgatttc

.1116 .1120 .1137 .1152

cgaacggtacata[a/t]gcc[g/t]tacacgtaacatgtaa[a/g]tgataatgcccagt[c/a]gtaatcgtt

.1182 .1219

ccaactctccatttcacacg[a/g]ccgtcaattttgaaatcgagccaaacgccgccgttt[t/a]gctggtcttctttcagatctggtccattaagaggtgaaatcggaacattgttactgaaaacgaacggcgaccatatgttaacgt

.1326 .1341 .1347 .1356

ctttatgaccttgataaactga[t/a]ggaatttgggtata[g/a]taagt[a/t]atttgttg[c/a]ttgtg

.1362 .1376 .1397

[a/g]tagttcgcgtatg[t/a]tttcatcttatcgtagtaaa[c/t]gccgattttgtcgttagggttccggg

.1424

[a/c]atatatagtgatttgaattgaagtggagaagatattgggagcggaaacgttgaaattgaatattgtagcgtcttggagaatgaat

TM30_VAMP-forward: 5’acttccagtggcaataaactc 3’

TM30_VAMP-reverse: 5’ attcattctccaagacgctac 3’

Ta: 57°C

*CaM-10*, calmodulin, PGSC0003DMG400007205

>Chr10:59617455..59616944

Agctccaggacatgataaatgaagtggatgctgatggtaatggaaccatcgacttcccagagtttttgaacctca

59617.376 .323

tggc[c/g]aggaagatgaaggatacagactcagaggaggagctgaaggaggcattcagag[t/a]gtttgacaa

.304 .259

ggaccagaa[t/a]gggttcatctctgctgctgagctccgtcatgtgatgactaacct[t/a]ggtgagaagct

.247 .205

[g/t]actgatgaagaagttgatgaaatgatcagggaggctgatgt[c/t]gatggcgatggacaaattaacta

.181 .131 .129 .125

[t/c]gatgagtttgttaaggtcatgatggccaagtgatttccctcttctgaag[t/c]t[t/g]ttt[t/g]tt

.104 .096 .059

taactgtgaaaaaagacc[t/a]aacattc[g/a]tcagactgggtcagctttgggataatggtttctttt[a/t

.046 .030

]acaaatttatcc[a/g]gttaggttgtacctt[c/t]ggatgaatgtaatgctctatcgtttggtggttgaatcactttctttctcttatttggttgtcttgtacctcggctgataaagttgt

TM29_CaM-10-forward: 5’agctccaggacatgataaatg 3’

TM29_CaM-10-reverse: 5’ acaactttatcagccgaggta 3’

Ta: 57°C

*BSDR4*, bacterial spot disease resistance protein 4, PGSC0003DMG400031476

>chr11:1495193..1495678

Ctttggaagagttgaaactctttggaaataattttgagcatttgcctcaaagcatatccgaacttggtgctcttc

1495.308

gattgttgaatttgtcagattgcaagaggcttacacagct[a/g]ccagaatttccacagcaattagacacaata

.339 .374

[c/t]atgcagattggagcaatgattcgatttgtaattc[g/a/t]ttgtttcagaatatatcgtcattgcagca

.416 .426 .437 .444 .453 .454

tgacatatgtac[t/a]tcagattcc[t/a]tatcattaag[a/c]gtgttc[a/t]ccactggg[g/a][a/g]

.469 .478 .483 .484

ggataatatcccaa[g/t]atggttcc[a/c]ccat[c/t][t/g]ggaagatggtacaagtatatttgtcaaat

.532 .549

tgcctaaaaattggtatg[t/c]aagtgagaacttcttg[t/g]gatttgctgtatgtttctctggtcaatttattgacggcatagctcacttgttttgttatcatgggaggccggtgacgtgtatcgcccagaaacttgccttatccagccgttcaaaatatcgttttatac

BSDR4-forward: 5’ctttggaagagttgaaactcttt 3’

BSDR4-reverse: 5’gtataaaacgatattttgaacgg 3’

Ta: 58°C

*KiTH-2*, kiwellin, PGSC0003DMG400008101 (TC1970259)

>chr11:41323549..41324501

4132.3615

Attgttggacgaagggagtatgagcgaatataatgatatactcgatctgttctagagatattgtat[g/a]tgta

.3622 .3638 .3674

cg[g/t]atacattattatgtg[c/t]aaggatacaatatatttcacttttattatacgagc[g/a]aatacaat

.3689 .3712 .3722 .3728 .3731

gtattt[c/t]gctacgttttaaaaccaaaata[t/c]atacttcta[t/g]gtgtc[g/a]tt[a/t]ttaagc

.3750 .3764 .3778 .3785

aaaatctaagtg[g/c]aaataacactatt[c/t]agtaatttttttc[t/c]taaaat[a/t]ttataattcat

.3805.3806 .3819 .3828 .3841

aaactaaa[a/t][t/a]ttttgactccga[t/c]cgaataaa[t/g]cctatgttttgg[c/t]aaaatg

.3848

[a/g]aaattgtacaccactagccgaaaatcacgactgaaaaataaattaatatgtggcctacttcttaattatt

.3943(Indel atat) .3987

ttattaatttattgcctatatatat|ctgcctatttgcaatatctttattactcatataaccacattct[c/g]g

atcaattcttacaaaaatagccactatgacaaaactagcctcaattctttctctttgcatcattttcacaacttt

.4072(indel) .4134

catctctt|gggcaattaatatgcattagaggaaaatgcaacgatgaccctgacgttggaaccagtatttg[c/t

.4176

]ggaggaggaggaggaagttctccttctcctccgtctactgg[t/c]tcaacgccaggtatgctaactcttaa

.4203 .4269

[c/t]gactttagtgaaggtggagacgggggtggtccatcagaatgtgacgagaaatatcatgataacaa[c/t]

.4322 .4333

gatagagtagtcgcgttgactactagatggtacgataatggttcaagatgtg[g/a]taaaatgata[c/a]gtattcgggccgataaaaatgggaagagtgtaacggctaaggttgtggatgaatgtgacataaaagatggttgtaaaaataatgtagtggatggttcaattgctgtgtggagagctttgggattgaatactgatgagggaagagttcctattacttggtccatggcct

KiTH-forward: 5’attgttggacgaagggagtatga 3‘

KiTH-reverse: 5’aggccatggaccaagtaatagga 3’

Ta: 67°C

*RuBisCo_bp*, RuBisCo large subunit-binding protein alpha subunit, PGSC0003DMG400001148

>Chr11:41842860..41843325

Aggtgacaattaccaaggactcaacaaccatcatcgctgatgcagcatcaaaggatgagatacaatctaggattg

4184.2963 .2969 .2972 .2981

ctcagcttaaaaaggagctgtttgagac[g/a]gactc[g/a]gt[g/a]tatgactc[c/t]gagaaacttgct

.3027 .3053 .3056

gagagaattgccaagctttctgggggtgttgcc[g/a]tcataaaggttggagctgcaacaga[g/a]gc[c/t]

.3059

ga[g/a]cttgaagaccgcaagcttcgtattgaggatgcaaagaatgcaacttttgctgcaattgaagagggaat

.3173 .3185

agtacctggtggtggtgctgcttttgttcatttatcaacttatgt[c/t]cctgccattaa[g/a]gcgaagatt

.3200.3201 .3218 .3221 .3242

Gaaga[c/t][c/g]cagatgaaagattggg[a/c]gc[t/c]gacatcattcaaaaggtaaa[g/a]aatgctc

.3261 .3268 .3275 .3296.3297

aaatatcaata[t/a]ctaaat[t/g]tatttc[g/a]ttttattttatatgatattg[t/a][t/g]tgattggacaaaccaaaagtaccattac

TM38_RuBisCo_bp-forward: 5’aggtgacaattaccaaggact 3’

TM38_RuBisCo_bp-reverse: 5’ gtaatggtacttttggtttgtc 3’

Ta: 57°C

*CaM-11*, calmodulin, PGSC0003DMG400027384

>Chr11:43823730..43824564

4382.3770 .3788

Gaaattactcaatcaaaaggtttcaaaaaaggacattcaa[g/a]gtccctaattgtagaaa[g/a]gtttgaat

.3814.3815

accaaatgctgaagagt[t/a][a/g]ctaacaaccaaactactaaaatgggaatagagcagaaaagcatataccaaagaacagcactacaaaacaatgcaaataggagaacaggcaagcaccaaaacaaagctaataatgtcaaagaac

.3949 .3981.3986.3994(indel+T).3995

acatcctaac[c/t]ggaaacagaaacctcatcaaacttatctgcc[g/a]ttcc[t/a]tttttttt|[c/g]c

.4005 .4030

cttttatg[t/a]tgtacttccaattttaaagattaa[g/a]atgagttgatcaattctcacttggccatcatga

.4071 .4098 .4116 .4118

ccttgac[g/a]aactcgtcatagttgatctgtccgtc[a/t]ccatccacgtcagcttc[g/a]c[g/a]aatc

.4125

at[t/c]tcatcaacctcttcgtctgtaagcttctcacctaggttagtcatgacatggcgaagctcagcagcaga

.4200 .4215

gataaa[t/g]ccattctgatcctt[g/a]tcaaacactctaaaagcttccttgagctcctcctcggaatcagtgtccttcatcttgcgagccatcaggttaaggaactcagggaagtcaatggtcccattcccatcagcatcaacttca

.4359 .4392

ttgatcatgtcttgaagctcagc[c/t]tcagttgggttctgccccaatgaccgcatcac[g/a]gttccaagctccttagttgtgatgcaacctgcagcacagttgcacatagttattggacatgttaagttttaagagcttacaagttacgaacacttacaactagcaattcacatcctccccccccccctcccccctctctttggttcttctttatgtatagatgggaaaaggc

MF17_CaM-11-forward: 5’gaaattactcaatcaaaaggttt 3’

MF17_CaM-11-reverse: 5’ gccttttcccatctatacataa 3’

Ta: 57°C

| *LapN,* leucine aminopeptidase N*,* PGSC0003DMG400007831  >chr12**:**2330749..2331596 233.0798  GCTTCCTGGTCTTGGCTCAAAAAGGATTGCTCTAGTTGGGCTTGGCTCA[C/T]CAACATCATCAACTGCTGCTTA  .0825 .0878(2704).0881(2707)  TCGC[T/A]GTTTAGGGGAGGCTGCTGCTGCAGCTGCCAAGTCTGCTCAGGCTAGTAATAT[C/T]GC[C/T]ATT  .0905(2731) .0920(2746)  GCTCTTGCTTCTACGGATGG[A/G]CTCTCTGCAGAATC[G/A]AAGCTTAGCTCTGCCTCTGCCATAACAACTGG  .0957(2783).0964(2790).0967(2793).0973(2799).0984(2810).0987(2813).0990(2816  TATC[C/G]ATTTTC[A/G]TG[G/A]TCTTC[G/A]CTTAATTCAT[T/C]GA[A/G]CC[A/G]TATTGAGAAA  .1005indel(+A)(2831) .1070  CTAA\|CTTGGTTGATTTTCGTGAACATTGTAGGAGCTGTGCTGGGGACATTTGAAGATAATAGGTTTAA[A/G]TC  .1091  TGAGTCAAAGAAACCAAC[A/G]TTGAAATCTTTGGATATTCTTGGACTGGGGACTGGACCTGAGATAGAGAAGAA  .1157 .1184 .1187  AATCAAGTATGC[A/T]GCAGATGTCTGTGCAGGTGTTATACT[C/T]GG[A/C]AGAGAGCTCGTCAATGCACCC  .1223  GCCAATGTACTTAC[G/C]CCTGGTTAGTGTTTTTCAATGCATTTCCTTGTTGTCCCTTTTATTAGTATGCCTATC  .1352  ACCACACTATTAAAATGCCGACTTTTCGCTGCAGCGGTACTTGCTGAAGAGGCCAAAAAGATTGCGTCCAC[T/C]  .1358 .1403  TATAG[C/T]GATGTCTTTTCTGCAAACATCTTGGATGTTGAGCAGTGCAAAGA[A/G]TTGAAAATGGGATCCTATTTAGCAGTTGCTGCAGCTTCTGCAAATCCTGCTCATTTCATCCATTTGTCTTATAAGCCTAGTAGTGGAGAAATAAAAAAGAAGATAGCCTTGGTTGGAAAGGGATTAACTTTTGACAGGTAATTCTATCTTCTATAAGTTGGAAAAATAGAAATTTGATTTCTGACCTGGCTGC  For primers and PCR conditions see Fischer et al. (2013)BMC Plant Biology 13:113.Numbers in parenthesis are the SNP positions counted from the ATG codon with A at position 1 according to Fischer et al. (2013).  *Jaz1*, jasmonate-zim domain protein 1 (TC208021), PGSC0003DMG400002930 |
| --- |
| >chr12:3178511..3177961  317.8441  cccatgaatcttttccctcaagaatttgatttctccaaagaacaatccacaaaaaagactgaatcttgga[a/t]  .8438 .8404  at[t/c]tgatcaaccagaaaaggcacaaatgaccatttt[t/c]tatggtggacaagtcattgtttttgatgat  .8318 .8315 .8313  tttccagctgataaagcaaatgagatcatgaaattagccaacaaacaaaacccca[c/t]aa[a/g]c[a/t]ac  .8306 .8277  ttca[c/t]ttatactatgatgaagaatcaaaaaaca[t/g]ctgatcaatctggtgcaaattttggtaacaaat  .8225 indel .8204  tgattcaagaactcccaaa\|gtgtcaagtgtcaatgccac[a/t]gccttctgttgctgatttaccaatcgc  .8176 .8122  [g/a]aggcggaattcacttacaagattcttggagaaaaggaaagatagagtaacatc[a/c]attgcaccatac  .8099  caaatctcca[g/a]caacaagaaatccaagaatgaggaaaacaaggcatggttgggattaggtgctcaatttgt  .8003.8002  Taaaactgagcaatacttttagttactattcttgg[a/t][t/a]tttcattttttgatgattgaagaaggggagtcttgacgtga  Jaz1-forward: 5’cccatgaatcttttccctca 3’  Jaz1-reverse: 5’tcacgtcaagactccccttc 3’  Ta: 57.4°C  *ATPD*, ATP synthase delta chain, chloroplastic, PGSC0003DMG400016959  >Chr12:54981348..54981943  54981.376 .381  Caaactccgatccaaacgcacctcccgc[c/t]gcgg[a/c]cgcggcggcggcgctctcggtgcaaaaatggcag  .423 .453  attccgc[g/c]gccggtagctacgcgaacgctctcgccga[t/c]gtagctaaatccaacgaaacactagaacaa  .492 .527  accaccgc[a/c]gatctcgaaaagatcgagaaaatcttcgacgacg[a/c]tgcggtttacgatttcttcgtaag  .585 .591 .597  ccctatcgtaagcgaagagaagaaacgtgaact[t/c]gtaga[t/c]gagat[g/c]gtttcatctacaggcatt  .651  caaccacacgttgcgaattttctcaacatccttgt[c/g]gatatgaagagaattgaattaatcaaagatattgtt  .715 .716 .720  aaagagtttgagaaagtttacaatacc[g/t][c/t]gac[t/g/a]gatactgaacttgctgtggtgacttcagt  .755 .757 .804 .807  ggtga[a/c]a[c/t]tggaatcgcaacatttagcacagatcgcaaaaggagttcaacgatt[a/g]ac[g/c]gg  .825  agcgaaaaatgtgag[g/a]atcaaaacagtgattgatgaatctcttgttgctggatttacaataaggtatggaaa  .903 .909  ttcaggatccaaattgattga[t/c]atgag[t/c]gtgaagaaacaacttgaagatattgctgcacagc  TM31_ATPD-forward: 5’caaactccgatccaaacgca 3’  TM31_ATPD-reverse: 5’ gctgtgcagcaatatcttcaa 3’  Ta: 57°C |
