# Supplementary figures and images for: Targeted and Untargeted Approaches Unravel Novel Candidate Genes and Diagnostic SNPs for Quantitative Resistance of the Potato (Solanum tuberosum L.) to Phytophthora infestans Causing the Late Blight Disease
Source: PLoS One. 2016 Jun 9;11(6):e0156254. doi: 10.1371/journal.pone.0156254 (PMC4900573; doi:10.1371/journal.pone.0156254)

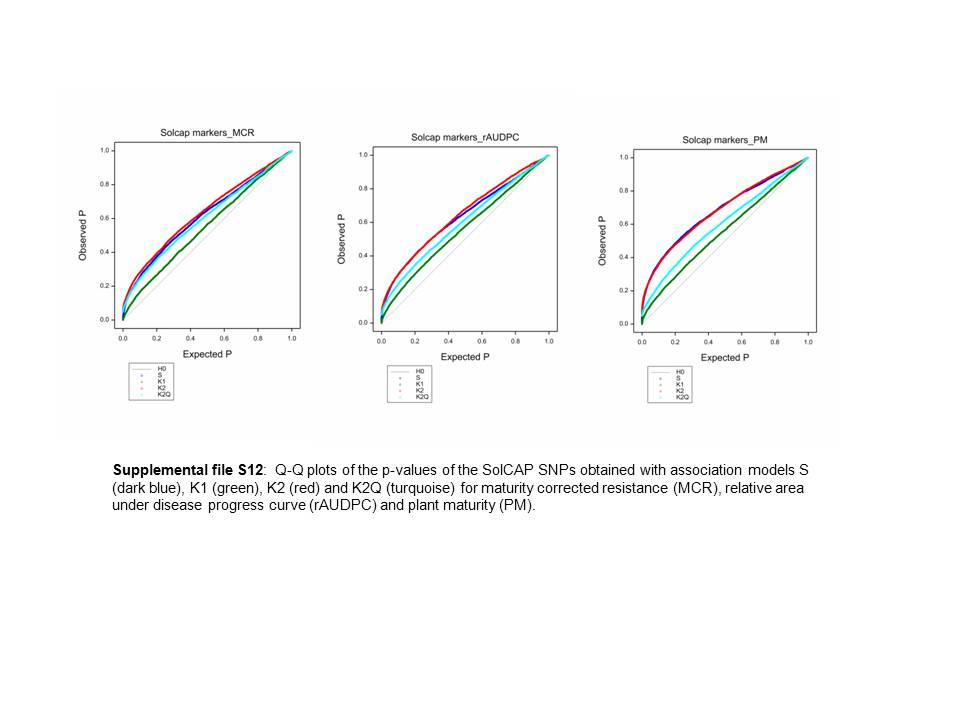

Supplement: S12 File — (JPG) [file pone.0156254.s012.jpg]
